# Deducing the conformational space for an octa-proline helix†‡

**DOI:** 10.1039/d3sc05287g

**Published:** 2023-12-21

**Authors:** Sara M. A. Waly, Andrew C. Benniston, Anthony Harriman

**Affiliations:** a Molecular Photonics Laboratory, Bedson Building, School of Natural and Environmental Sciences, Newcastle University Newcastle upon Tyne NE1 7RU UK anthony.harriman@ncl.ac.uk

## Abstract

A molecular dyad, PY-P_8_-PER, comprising a proline octamer sandwiched between pyrene and perylene terminals has been synthesized in order to address the dynamics of electronic energy transfer (EET) along the oligo-proline chain. A simple pyrene-based control compound equipped with a bis-proline attachment serves as a reference for spectroscopic studies. The N–H NMR signal at the terminal pyrene allows distinction between *cis* and *trans* amides and, although the crystal structure for the control has the *trans* conformation, temperature-dependent NMR studies provide clear evidence for *trans*/*cis* isomerisation in D_6_-DMSO. Polar solvents tend to stabilise the *trans* structure for the pyrene amide group, even for longer oligo-proline units. Circular dichroism shows that the proline spacer for PY-P_8_-PER exists mainly in the all-*trans* geometry in methanol. Preferential excitation of the pyrene chromophore is possible at wavelengths in the 320–350 nm range and, for the dyad, is followed by efficacious EET to the perylene emitter. The probability for intramolecular EET, obtained from analysis of steady-state spectroscopic data, is *ca.* 80–90% in solvents of disparate polarity. Comparison with the Förster critical distance suggests the terminals are *ca.* 18 Å apart. Time-resolved fluorescence spectroscopy, in conjunction with DFT calculations, indicates the dyad exists as a handful of conformers displaying a narrow range of EET rates. Optimisation of a distributive model allows accurate simulation of the EET dynamics in terms of reasonable structures based on isomerisation of certain amide groups.

## Abstract 2

The all-*trans* dyad can function as a molecular ruler but complications arise from the *cis*-configuration.

## Graphical abstract

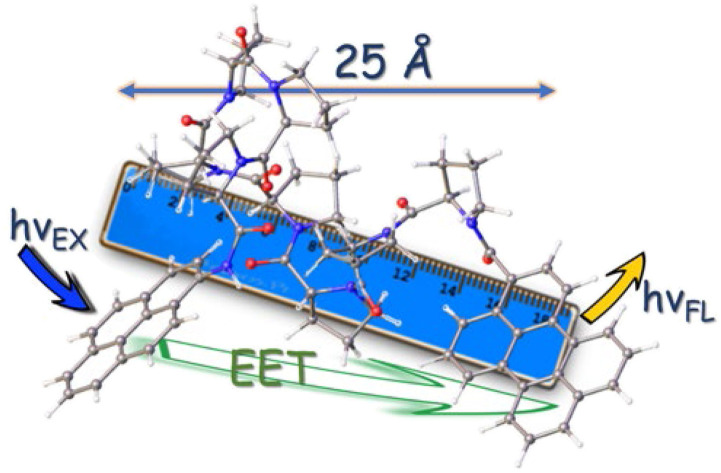

## Introduction

The concept of a covalently-linked molecular dyad, whereby a well-defined connecting group spatially isolates specific donor and acceptor units, has been instrumental in the development of molecular photophysics. The most informative dyads are those where the spacer is a rigid connector maintaining close control of the geometry of the entire system by imposing a narrow distribution of separation distances and mutual orientations.^1–5^ In most, but not all, cases the spacer should not be electronically coupled to either donor or acceptor, nor should it operate as a relay for electron or exciton transfer between the terminals. Such dyads have led to the observation of the Marcus inverted region,^6,7^ and its subsequent quantum mechanical modification,^8^ and to recognising the limitations of Förster theory for dipole–dipole interactions at short separations.^9–12^ Related dyads have been designed to explore the structural demands for excitonic coupling,^13^ proton-coupled electron transfer,^14^ photochromism^15^ and E-type delayed fluorescence.^16,17^ A particular success for rigid molecular dyads has been the study of electron and exciton transfers across orthogonal spacer units.^18–20^ Strategies are now in place that permit photophysical measurements to be made with donor and acceptor groups at predetermined distances from *ca.* 120 Å to *ca.* 5 Å.^21–24^ However, intramolecular torsional perturbations are difficult to eliminate with long spacers constructed by accretion of several small units such that actual distances and orientations might not be as expected from simple molecular models.^25,26^ With few exceptions, these dyads are expensive to prepare and require skillful synthesis that realistically cannot be scaled-up. It is worth noting that the humble dyad has been used to construct a plethora of more exotic molecular architectures, ranging from linear oligomers to cyclic superstructures.^27–31^

Considerable interest has been shown in the development of molecular dyads having a long, linear spacer that facilitates examination of how separation distance affects the rate of a particular chemical process.^32–36^ This is usually achieved by linking together several identical small spacer units, such as phenyl rings, but this does not guarantee that the spacer will adopt the fully extended conformation. An alternative route to elongated spacers is to adapt biological media to host the donor and acceptor units. This can be done, for example, using polynucleotides with intercalating reagents^37^ or by attaching the active chromophores to discrete sites on peptides.^38^ The latter procedure is particularly attractive since it allows the use of automated synthesis to assemble dyads with pre-programmed composition and length.^39^ One concern with this approach is that the amide connections can adopt *cis* and *trans* conformations,^40^ such that a unique geometry might not emerge. In the case of peptide-based spacers, rigidity is realised if the oligomer forms a helical assemblage.^41^

In this respect, we have considered using polyproline residues as inert spacers since proline is unlikely to perturb the electronic properties of the terminal, has good solubility in a range of solvents and is readily functionalised. Polyprolines form both right-handed and left-handed helices according to the geometry around the peptide bonds; *trans* conformations give rise to a left-handed helix (PPII) whereas the corresponding *cis* conformation favours a right-handed helix (PPI).^42^ Internal hydrogen bonding is insignificant in either structure. The PPII helix is relatively open and more stable than PPI,^43^ with interconversion between the two forms being slow due to the high activation energy for *cis*–*trans* isomerisation.^44^ Previous research^45^ has proposed that the PPII structure is sufficiently rigid for use as a “molecular ruler” in structural biology. Specifically, PPII residues have been labelled with terminal fluorophores chosen for their capacity to undergo unidirectional electronic energy transfer (EET) along the helix.^46^ More recent work,^47^ however, has raised questions about the rigidity of short polyproline residues which might invalidate their use for the determination of *in situ* separation distances. This uncertainty has prompted us to examine the dynamics of EET along a proline-based octamer decorated with emissive terminal chromophores. For the emitters, we have opted to use the classical pair of pyrene and perylene since these polycycles are of similar dimensions, possess well-defined transition dipole moment vectors and exhibit complimentary photophysical properties. There have been several studies of EET from pyrene to perylene, starting in 1967 (ref. 48) and including the use of a polynucleotide scaffold to provide incremental separation distances.^49^ The Förster critical distance^50^ for this pair is *ca.* 23 Å while the spectral overlap integral^51^ is reported to be *ca.* 2.4 × 10^−14^ cm^6^ mmol^−1^. Since pyrene possesses a relatively long-lived excited singlet state, these parameters should enable effective EET over considerable distances.

Our primary interest in this area is to establish if polyproline can function as a suitable spacer for directed EET. This requires that the proline-based spacer is sufficiently long to form a tight helix and that there is a unique arrangement of *cis*- or *trans*-amide linkages. A further point of concern relates to quantifying any electronic interaction between chromophore and spacer, this being a particular issue for pyrene where the photophysical properties can be manipulated by substitution.^52^ The minimum accretion number for formation of an oligo-proline helix^53^ is *ca.* 6 and we have opted to prepare an octamer as the basic spacer unit. For this system to be effective, it is essential that there is a narrow distribution of molecular lengths and orientations. The latter should become apparent from time-resolved fluorescence spectroscopic measurements^54^ made to monitor through-space EET between the terminals. Quantum chemical calculations have allowed identification of key structures in order to compare simulated rates of EET with the experimental measurements.

## Results and discussion

### Compound synthesis and characterisation

**Scheme 1:**
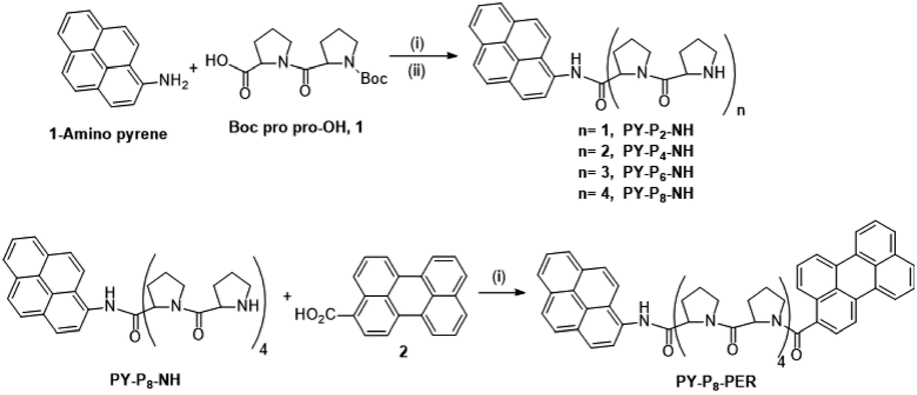
Reagents and conditions: (i) COMU, DIPEA, RT, DMF, overnight, (ii) 4 M HCl, dioxane, RT, overnight. A detailed synthetic route is given in Scheme S1.‡

The linear preparation of PY-P_8_-PER (Scheme 1) has the advantage, relative to automated syntheses,^55^ that at each stage the compounds can be purified and fully characterised. Our approach started from the pyrene segment of the compound, in the form of 1-aminopyrene, reacting with the commercially available Boc protected carboxylic acid 1 using the coupling agent COMU^56^ and DIPEA as base^57^ to produce PY-P_2_ (ESI Pages S2–S23‡). The successful synthesis of PY-P_2_ was confirmed from its ^1^H NMR spectrum (Fig. S1‡) by the appearance of the signal for the Boc (*i.e.*, *tert*-butyloxycarbonyl) methyl protons at 1.47–1.52 ppm, as well as the broad signals in the range between 1.80–5.13 ppm, which are assigned to the proline aliphatic CH_2_/CH protons. Confirmation of the molecular composition of PY-P_2_ was obtained by positive ESI mass spectrometry (CH_2_Cl_2_/CH_3_OH + NH_4_OAc) which displayed a peak at *m*/*z* = 534.2363 au corresponding to the [M + Na]^+^ ion and a peak at *m*/*z* = 529.2809 au corresponding to the [M + NH_4_]^+^ ion (Fig. S25‡). Deprotection of PY-P_2_ using 4 M HCl in 1,4 dioxane afforded PY-P_2_-NH in near quantitative yield. A diagnostic signal is provided by the NH proton, which was identified as a singlet at 10.99 ppm and confirmed its successful preparation (Fig. S9‡). Coupling of the carboxylic acid 1 with deprotected amino proline PY-P_2_-NH afforded PY-P_4_ in good yield (67%), before deprotection to give PY-P_4_-NH in excellent yield.

By consecutive coupling of the carboxylic acid 1 and deprotected amino-proline derivatives, the octamer PY-P_8_-NH was prepared. At each coupling stage, the new compounds were purified and fully characterised (see ESI‡). Confirmation of the molecular composition of PY-P_8_-NH was obtained by positive ESI mass spectrometry (CH_2_Cl_2_/CH_3_OH + NH_4_OAc) which displayed a peak at *m*/*z* = 994.5185 au, corresponding to the [M + H]^+^ ion, and a peak at *m*/*z* = 1016.5005 au, corresponding to the [M + Na]^+^ ion (Fig. S32‡).

In the final step, the perylene carboxylic acid 2 was coupled to PY-P_8_-NH to produce the dyad PY-P_8_-PER in reasonable yield (42%). The broad signals of the ^1^H NMR spectrum in the region between 1.99–4.76 ppm (56 protons) are consistent with the number of proline aliphatic CH_2_/CH protons (Fig. S21‡). The mass spectrum displayed a peak at *m*/*z* = 1272.5894 au, consistent with the [M + H]^+^ ion (rmm = 1272.58), a peak at *m*/*z* = 1289.6183 au, corresponding to the [M + NH_4_]^+^ ion, (rmm 1289.62) and a peak at *m*/*z* = 1295.5768 au, corresponding to the [M + Na]^+^ ion (rmm 1294.58) (Fig. S33‡).

### Stereochemistry at the pyrene terminal

**Fig. 1:**
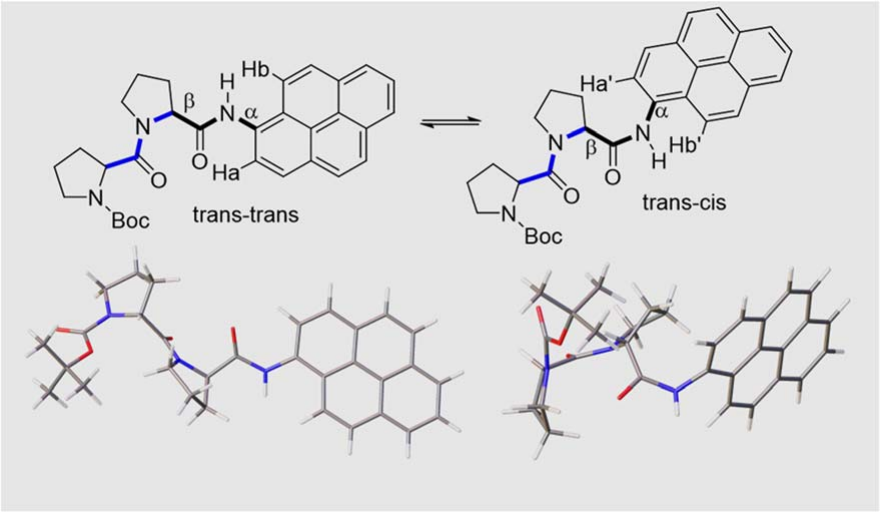
The upper panel shows two possible isomers of PY-P_2_ originating from amide bond rotation and highlighting the *trans*/*cis* assignments at the proline (blue) and pyrene site (black). Note: bond rotation at bond α also interconverts the positioning of protons H_a_ and H_b_ and modifies their chemical environment. The bond rotation β brings the bis-proline and pyrene groups within close proximity. The lower panel shows energy-minimised structures for the *trans*–*trans* and *trans*–*cis* isomers in CHCl_3_, emphasising the geometry at the pyrene amide group.

Close inspection of the ^1^H NMR spectrum for PY-P_2_ in CDCl_3_ indicates two peaks, with near equivalent intensities, at 10.20 ppm (53%) and 10.27 ppm (47%) (Fig. S1‡). Both peaks disappear upon a D_2_O shake, confirming these are associated with the amide NH-unit (Fig. S3‡). The pattern is consistent with two slowly interconverting conformers in solution. From inspection of the molecular structure, two isomers are feasible based solely on bond rotation at the pyrene-amide bond (Fig. 1). It is noted that *trans* to *cis* isomerisation modifies the chemical environment of H_a_, moving it away from the CO group. Even though proton H_b_ is always proximal to the NH group, there is a change in the NH magnetic environment since it is within the deshielding zone of the CO subunit. Therefore, the chemical shifts for H_a_/H_a′_ and H_b_/H_b′_ are expected to differ for the two isomers. The NMR signals due to the *t*-butyl groups overlap and it was not possible to separate them.

**Fig. 2:**
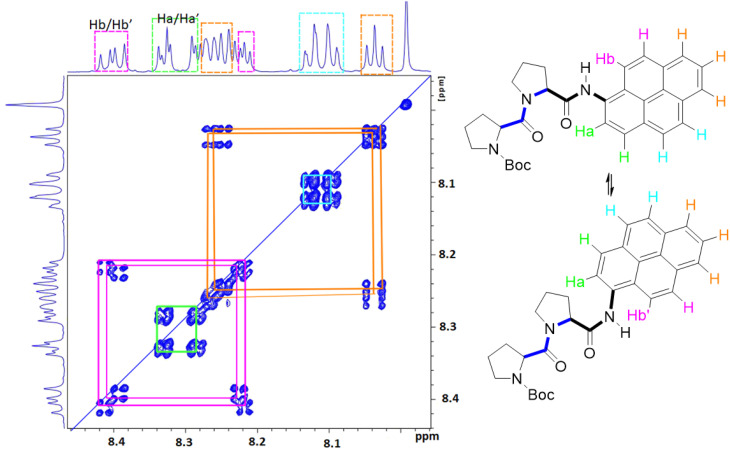
Selected COSY NMR spectrum (700 MHz, D_6_-DMSO) recorded for compound PY-P_2_ and the colour coded interpretation for the two isomers.

Due to the dynamic properties of peptide bond isomerisation,^58^ many nuclei offer potential as probes in NMR spectroscopy for determining both interconversion kinetics and the relative concentrations.^59^ Thus, selected aromatic proton resonances for PY-P_2_ were assigned by a combination of COSY and ROESY NMR spectra (Fig. S5–S7‡). The COSY data could be interpreted as a four-spin system, as identified in the coloured plot shown in Fig. 2. The spin system comprising two protons (blue) is clearly correlated and did not show NOE interactions to the proline protons. The three protons coloured brown are also readily identified. The two most downfield doublets (pink) are assigned to protons H_b_/H_b′_ since they show a strong NOE to the N–H proton as well as to several proline protons; the corresponding coupled proton is readily identified by the cross-correlation contour. The final set of protons is assigned to H_a_/H_a′_ (green) and as expected displays only a weak NOE to the N–H proton. The most deshielded proton is assigned as H_a_ (*trans*–*trans* isomer) due to the carbonyl anisotropy and hydrogen bonding effect, this being consistent with the X-ray structure discussed below.

**Fig. 3:**
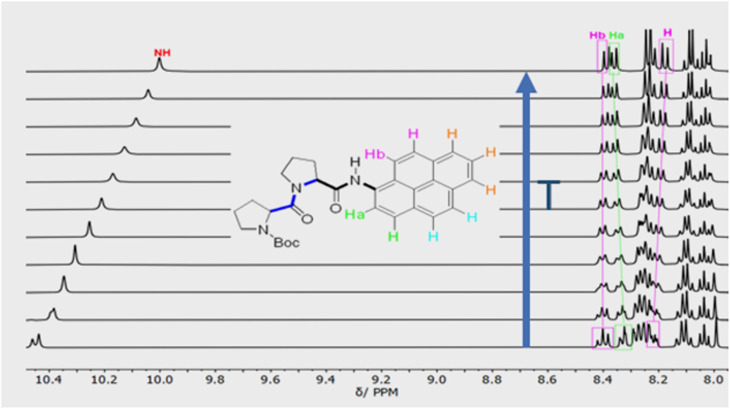
Variable temperature ^1^H NMR spectra of PY-P_2_ in D_6_-DMSO showing the aromatic region and the most downfield proton resonances. The blue arrow indicates the direction of increasing temperature. The first spectrum was recorded at 298 K, the second at 313 K and subsequent spectra were recorded at intervals of 10 K until 403 K.

Confirmation of *cis*–*trans* isomerisation was obtained by collecting ^1^H NMR spectra for PY-P_2_ in D_6_-DMSO from 298 to 403 K (Fig. 3), the solvent being chosen for its low volatility. The two distinct N–H peaks at room temperature collapse into one resonance at around 323 K, which broadens and shifts upfield by around 0.47 ppm as the temperature is raised further. An additional feature is observed with increasing temperature; namely, the H_a_/H_a′_ protons shift upfield by *ca.* 0.5 ppm while the H_b_/H_b′_ protons shift downfield by *ca.* 0.4 ppm. These resonances converge and finally collapse to two doublets as expected for fast *trans*/*cis* exchange. At high temperature, the proton coupled to H_b_ is clearly visible as a doublet.

**Fig. 4:**
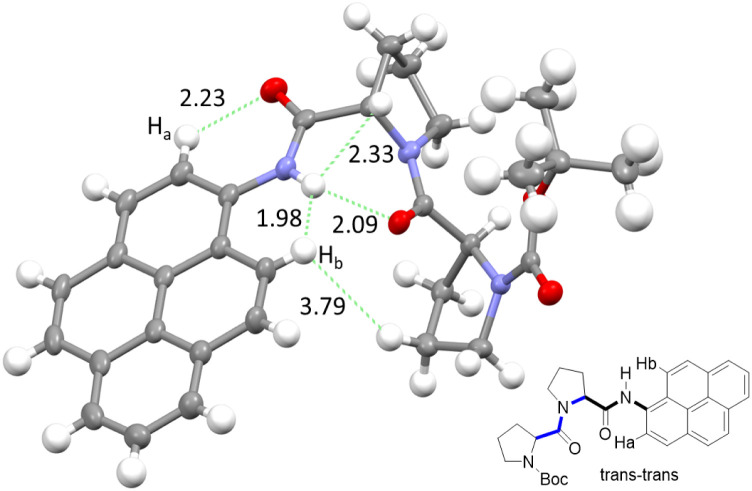
X-ray crystal structure of PY-P_2_ shown as thermal ellipsoids (30%) and with selected intramolecular distances between atoms (in Å) with carbon (grey), hydrogen (white), nitrogen (blue) and oxygen (red). Insert shows chemical structure, highlighting important protons (H_a_ and H_b_) and their relation to the crystal structure.

The NMR spectroscopic interpretation was validated by the collection of X-ray crystallographic data for a single-crystal of PY-P_2_ (Fig. 4). The solid-state structure corresponds to the *trans*–*trans* isomer. The bis-proline subunit appears to be “wrapped” around the pyrene group rather than extended as drawn in the simple chemical formula shown in Fig. 1. This feature is driven by the intramolecular hydrogen bond between the N–H amide of the pyrene and the central carbonyl group of the bis-proline. The proton assigned to H_a_ is evidently within the sphere of influence of the carbonyl group, as inferred from the NMR experiments. Several close contacts are identified for proton H_b_, especially noting its short distance to the N–H proton of the pyrene amide. One of the proline protons resides well within the range (*ca.* 5 Å) needed to effect an NOE effect. Consequently, the solid-state structure appears to correlate satisfyingly well with that inferred from the NMR spectra.

By close inspection of the ^1^H NMR spectra for PY-P_4_, PY-P_6_, PY-P_8_ and PY-P_8_-PER in deuterated chloroform (Fig. S11, S15, S19 & S21‡), a pair of resonances at *ca.* 10.0 and 10.7 ppm was noted and assigned to the N–H proton at the pyrene unit for the PPI and PPII conformational structures, respectively. In the shorter oligomers, each resonance has two closely spaced peaks, consistent with *cis*–*trans* isomers at the pyrene-amide bond, as described for PY-P_2_. The downfield resonance corresponds to *cis*, while the upfield resonance relates to *trans*. As illustrated in Fig. S21,‡ the ratio of *trans* : *cis* is around 3 : 2 (*K*_eq_ = 1.5) for PY-P_8_-PER. When dissolved in the polar solvent D_6_-DMSO the spectrum is simplified with the downfield resonance reduced in intensity and a more intense signal observed at 10.35 ppm (Fig. S23‡). The *trans* : *cis* ratio is *ca.* 47 : 3 (*K*_eq_ ∼ 16). This observation is consistent with literature reports that in a polar solvent the *trans* structure (*i.e.*, PPII) is dominant. It is noted that chemical shifts for the N–H resonance of the *cis* structure are comparable in CDCl_3_ and D_6_-DMSO. In contrast the N–H resonance for the *trans* structure is shifted upfield by around 0.3 ppm with the increase in solvent polarity. The N–H environment for the pyrene-amide would appear to not alter significantly in the *cis* structure. The nature of the solvent might influence any internal hydrogen bonding.

Variable temperature ^1^H NMR spectra recorded for PY-P_8_-PER in D_6_-DMSO (Fig. S23‡) over a temperature range from 298 to 403 K were equally informative. With increasing temperature, the two N–H resonances shift upfield, which is consistent with the shift seen for PY-P_2_. The ratio PSII : PSI decreased to around 22 : 3 (*K*_eq_ ∼ 7) at 353 K. The N–H peak assigned to the *trans* structure is broadened, and the resonance for the *cis* structure is indiscernible from the background noise at around 373 K. It is inferred that the two structures are in fast exchange on the NMR timescale at this temperature.

Molecular structures calculated by DFT (B3LYP/6-311G(d,p)/PCM) methods^60^ for *cis* and *trans* geometries of PY-P_2_ in CHCl_3_ are shown in Fig. 1 and key structural information is given in Table 1. Further details are given in the ESI.‡ For the energy-minimised structures, the amide C–N bond is slightly shorter for the *cis*-isomer while the C=O bond is longer. This suggests that the amide bond possesses somewhat more double-bond character for the *cis*-geometry.^61^ This might arise because the connecting C_aryl_–N bond is slightly shorter for the *cis*-isomer while the dihedral angle between the polycycle and the amide C–N bond is lower than for the *trans*-isomer (Table 2). These structural facets, especially the improved planarity, can be used to argue that the *cis*-isomer interacts more strongly with the polycycle. No internal hydrogen bonds could be detected for either structure. Interestingly, the second amide has the same configuration as the terminal amide, giving rise to *trans*,*trans* and *cis*,*cis* structures as the energy-minimised species (see ESI, Section S6,‡ for details). Of these, *trans*,*trans* is the lowest-energy conformer. The energy difference between *trans*,*trans* and *trans*,*cis* in CHCl_3_ solution is only *ca.* 1.3 kcal mol^−1^ while that between *cis*,*cis* and *cis*,*trans* is slightly smaller. The activation barriers for these isomerisation steps in CHCl_3_ are calculated to lie in the range of 12–15 kcal mol^−1^.

**Table 1:** Structural parameters calculated by DFT (B3LYP/6-311G(d,p)/PCM) methods^a^ for PY-P_2_ in CHCl_3_

Parameter	*Cis*-isomer	*Trans*-isomer
N–H/Å	1.0132	1.0076
C–N/Å	1.3618	1.3906
C=O/Å	1.2394	1.2284
C_aryl_–C/Å	1.4185	1.4204
C–C–N–C(O)^b^/°	5.24	7.83

a See ESI for details and Fig. 1 for 3D representations of the two structures.

b Refers to the dihedral angle between the pyrene ring and the amide C–N bond.

**Table 2:** Compilation of the photophysical properties recorded for the control compound and the perylene terminal in dilute CHCl_3_ solution at room temperature and comparison with pure pyrene and perylene

Parameter	PY-P_2_	Pyrene	PER	Perylene
*λ*_ABS_ a/nm	383	372	443	436
*λ*_MAX_ b/nm	344 (32 300)	343 (34 700)^c^	NA	NA
*ε*_MAX_/M^−1^ cm^−1^	3400	330^d^	39 500	38 500
*λ*_FLU_/nm	392	373	460	436
Δ_SS_/cm^−1^	130	70	830	<30
*ϕ*_F_	0.60	0.58^e^	0.91	0.87^e^
*τ*_S_	14.5 (11.2 : 16.0)^f^	450^f^	6.2 (4.3 : 6.6)^f^	6.0^e^
*τ*_T_ g/μs	450	510	690	4000^h^

a Absorption band corresponding to the 0,0 transition.

b Absorption maximum used to determine the solute concentration with the molar absorption coefficient (M^−1^ cm^−1^) given in parenthesis.

c Taken from ref. 68.

d Taken from ref. 69.

e Taken from ref. 79.

f Excited-singlet state lifetime extracted from a mono-exponential fit of the decay data in N_2_-saturated solution. Values in parenthesis refer to a fit to dual-exponential kinetics.

g Triplet-excited state lifetime determined for deoxygenated solutions at low concentrations (<10 μM).

h Taken from ref. 81 and 82.

### Stereochemistry of the polyproline spacer

Polyproline can adopt two disparate structural conformations, commonly referred to as PPI and PPII in solution and the solid state.^60^ The left-handed PPII structure has a helical pitch of *ca.* 9.0 Å with 3.0 proline residues per turn and contains all *trans* amide bonds; this structure is known to dominate in a polar solvent.^42,43,45,62^ The right-handed PPI structure, comprising all *cis* amide bonds, is found in less polar solvents and generates a more densely packed helix with a pitch of *ca.* 5.4 Å, with 3.3 proline residues per turn. A mixture of PPI and PPII structures may exist in weakly polar solvents.^63^

In prior studies it was demonstrated^64^ that a PPI helix displays a negative CD band at 199 nm, with a stronger positive band at 205–215 nm. In contrast, the PPII helix has a strong negative CD band at 205–210 nm and a weak positive band at 226 nm.^64^ To probe the helicity of the oligo-proline spacer in PY-P_8_-PER, CD spectra were recorded in methanol. An example is illustrated in Fig. S50‡ and shows a strong negative band centred at around 212 nm, which is consistent with dominance of the all-*trans* helix. This finding is in agreement with previous studies demonstrating the stabilisation of the PPII helix in a polar protic solvent.^42,65^

**Fig. 5:**
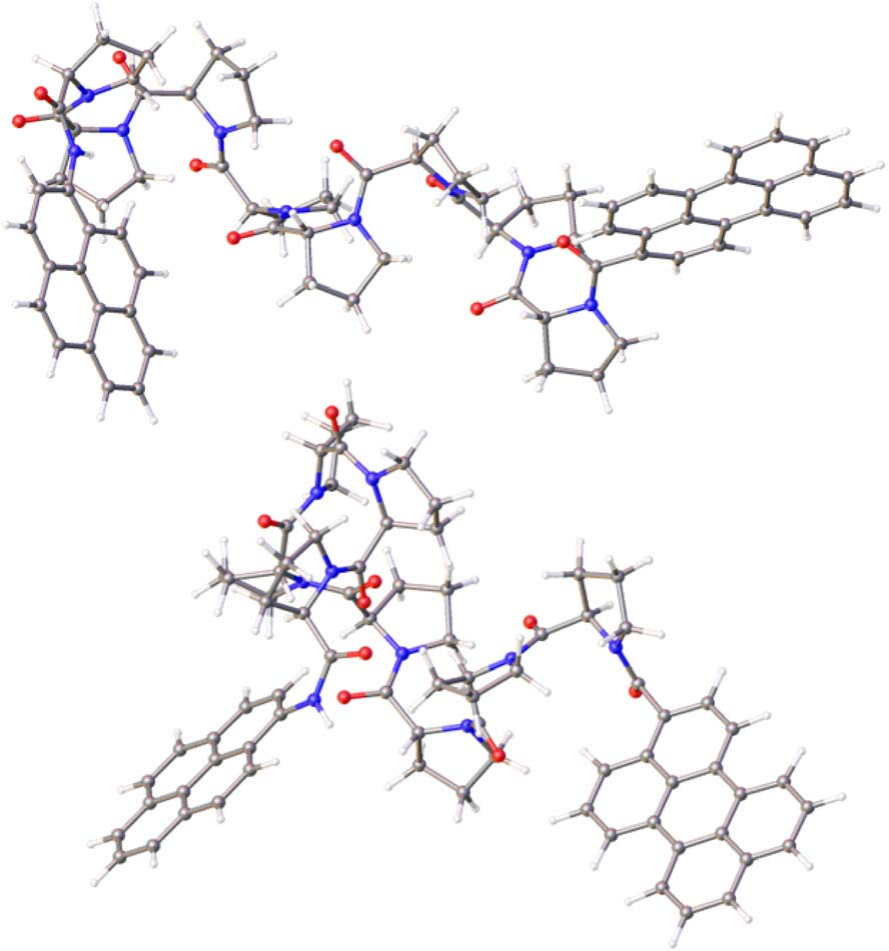
Energy-minimised structures computed for the *trans*-(upper panel) and *cis*-amides (lower panel) focussing on the mutual orientation of the aryl polycycles. The centre-to-centre separations are 19.1 Å and 14.6 Å, respectively, for *trans* and *cis* structures, while the twist and fold angles are 23.5° and 45.6° for the *trans*-amide and 68.7° and 101.6° for the *cis*-amide. The angles between the planes defining the polycycles are 74.3° and −8.5°, respectively, for *trans*- and *cis*-amides.

Calculations made at the DFT (B3LYP/6-311G(d,p)) level for PY-P_8_-PER in CHCl_3_ using the PCM treatment indicate that the lowest-energy species has an all-*trans* alignment of the amide bonds, including the terminal pyrene-amide unit (Fig. 5). This corresponds in a crude sense to the PPII structure with some of the central proline units forming a helix, in accord with the experimental results. This arrangement places the polycycles at a centre-to-centre separation of 19 Å. The lowest energy species with the terminal amide in the *cis* geometry also has *cis*-amides at the 3- and 7-sites, with all the other amides in the *trans*-structure (Fig. 5). This species is less stable than the all-*trans* arrangement by *ca.* 2 kcal mol^−1^ and has the polycycles at a separation distance of only 15 Å (Fig. 5). A further stable structure starts with the *trans*,*cis* species, followed by an all-*trans* chain (Fig. S65‡). Random conformer searches, using the Monte Carlo random searching algorithm and the MMFF94 force field implemented in Spartan™ indicated that the all-*cis*-isomer was not to be found among the 25 lowest-energy structures. Indeed, most *cis*-amides occur at the 1-(*i.e.*, the pyrene amide) or 3-sites (Fig. S63 & S64‡).

### Photophysical properties of the pyrene-based terminal

The photophysical properties of pyrene depend on the polarity of the solvent,^52,66^ as well as solute concentration,^67^ and the nature of any substituents.^68^ This sensitivity arises because the energy of the ^1^L_b_ state, which is below that of the ^1^L_a_ state, can be perturbed by structural and/or environmental changes. Most literature reports refer to the absorption band centred at around 344 nm and avoid the lower-energy transitions occurring across the wavelength range from 350 to 380 nm, where absorption is very weak (Fig. S34‡). Hara and Ware^69^ located the 0,0 transition as being at 372 nm with a molar absorption coefficient of 330 M^−1^ cm^−1^. To explore the effect of the amide connection on the optical properties of the pyrene chromophore, we synthesized the control compound PY-P_2_, which has an amide connection *via* the nitrogen atom at the pyrene nucleus, together with two proline residues (Scheme 1).

**Fig. 6:**
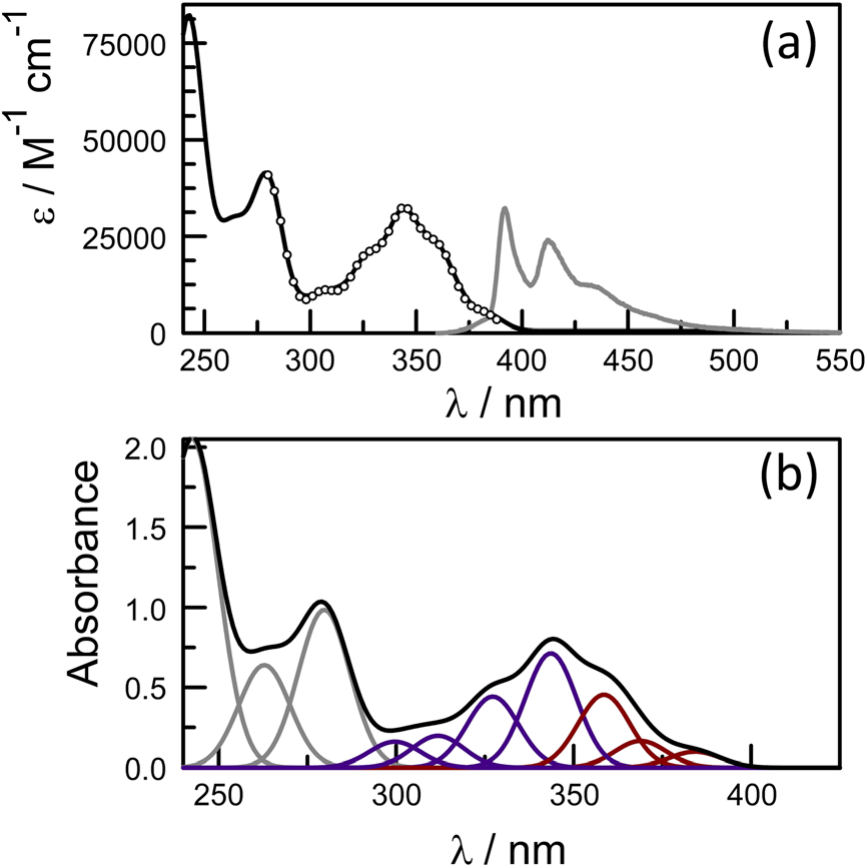
(a) Absorption (black curve) and normalised fluorescence (grey curve) spectra recorded for PY-P_2_ in CHCl_3_ solution (5 μM). The excitation spectrum is superimposed over the absorption spectrum as open circles. (b) Deconstruction of the absorption spectrum into Gaussian components in order to isolate the ^1^L_b_ (shown as brown curves) and ^1^L_a_ (shown as blue curves) states.

Absorption and emission spectra were recorded for dilute CHCl_3_ solutions (Fig. 6) and the respective maxima (*λ*_ABS_ and *λ*_FLU_) are listed in Table 2. The lowest energy absorption transition for PY-P_2_ is weak but significantly stronger than that reported for pure pyrene. The 0,0 transition is located at 383 nm, corresponding to a red shift of *ca.* 770 cm^−1^ relative to pyrene under the same conditions, where the molar absorption coefficient (*ε*) is 3400 M^−1^ cm^−1^. At higher energy, there is a more intense absorption transition, centred at 344 nm (*ε*_MAX_ = 32 300 M^−1^ cm^−1^) which remains comparable to that found for pure pyrene. The fluorescence spectrum is resolved into vibronic components but there is little mirror symmetry with the corresponding absorption transition. The Stokes shift (*Δ*_SS_) is small and signifies that only minor geometry changes accompany evolution of the relaxed excited-singlet state while excellent agreement was observed between excitation and absorption spectra (Fig. 6). The fluorescence quantum yield (*ϕ*_F_) and excited-singlet state lifetime (*τ*_S_) were recorded for dilute, deoxygenated CHCl_3_ solutions and are also given in Table 2. Relative to pure pyrene in dilute solution, the quantum yield and lifetime recorded for PY-P_2_ are decreased somewhat. It was noted that the reduced chi-squared parameter (*χ*^2^ = 1.33), used as a primary indicator of the quality of the statistical fit for the lifetime,^70^ is too high for a satisfactory analysis (Fig. S39‡). A much improved fit (*χ*^2^ = 1.16) was obtained using a dual-exponential model^71^ with the longer-lived species accounting for 63% of the initial population (Table 2 and Fig. S40‡). The two derived lifetimes, however, are too similar for accurate analysis and should be taken cautiously. The mean excited-state lifetime (〈*τ*〉) for the pyrene donor is 14.2 ± 0.6 ns.

At high concentrations (>5 mM) in nonpolar solvents, pyrene forms an emissive excimer,^72^ which in CHCl_3_ solution emits at 485 nm (Fig. S37‡). This latter species, which also forms in crystals^73^ and condensed films,^74^ has been exploited as the basis of numerous disparate types of fluorescent sensor.^75^ In thin films, the excimer transfers excitation energy to perylene present as a dopant.^74^ Under similar conditions, PY-P_2_ shows strong self-absorption, most notably by the extinction of the emission band centred at 392 nm, but does not form an emissive excimer in observable yield (Fig. S37‡). This situation is most likely a consequence of steric blocking by the bis-proline residue preventing π-stacking of two polycycles.

Excitation of deoxygenated ethanol solutions of PY-P_2_ with a 4 ns laser pulse at 340 nm results in formation of the triplet-excited state, which shows a prominent differential absorption peak at *ca.* 425 nm.^76^ Less significant absorption transitions are seen at longer and shorter wavelengths (Fig. S38‡). The triplet state is quenched by molecular oxygen and undergoes bimolecular annihilation at high laser intensity (Fig. S40‡).^77^ At low intensity, in the absence of oxygen, the triplet state decays *via* first-order kinetics with a lifetime of 450 μs in dilute solution (Fig. S39‡). The fit to a first-order process is satisfactory but the derived lifetime is comparable to the interconversion time estimated from the Eyring expression so that the exponential kinetics do not rule out there being an equilibrium mixture of *cis* and *trans* isomers. Phosphorescence spectra recorded^78^ in an ethanol glass at 77 K allow estimation of the triplet energy as being 48 kcal mol^−1^ (*i.e.*, 595 nm), which is slightly lower than that observed for pure pyrene under the same conditions.

The net conclusion is that the proline residue, and in particular the amide group, impacts somewhat on the photophysical properties of the pyrene chromophore, especially affecting the radiative rate constant. The low energy region of the absorption spectrum is intensified by way of increased mixing with the higher-lying state. The main consequence of such perturbations is that pure pyrene cannot be used as a reference compound for the EET donor. Furthermore, the apparent co-existence of two isomers is likely to complicate the dynamics of the EET event.

### The perylene terminal in the dyad

**Fig. 7:**
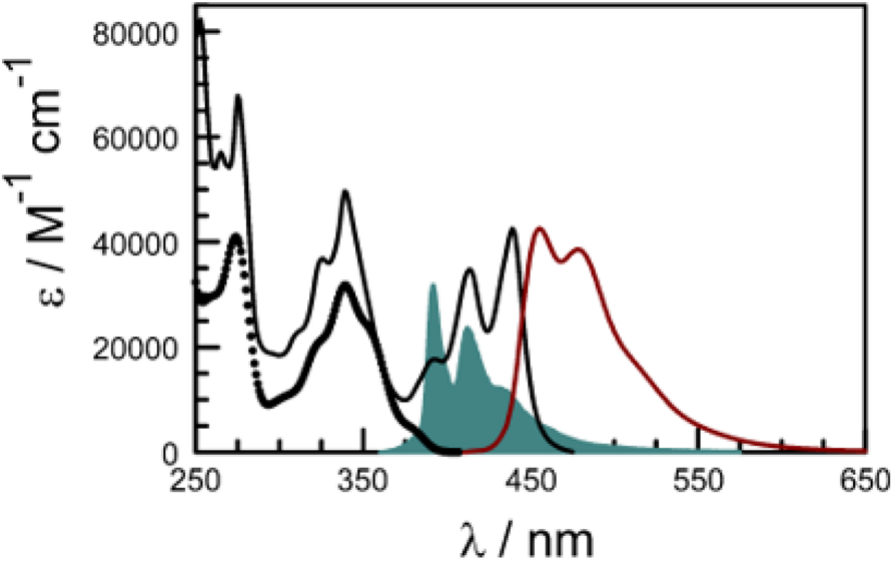
Absorption (black curve) and fluorescence (brown curve) spectra recorded for PY-P_8_-PER in CHCl_3_ at room temperature. The excitation wavelength was 420 nm. The open circles indicate the absorption spectrum for the pyrene component extracted by global curve fitting routines while the solid blue curve shows the normalised fluorescence spectrum for the pyrene emitter to indicate the good spectral overlap.

Rather than use a further control compound to mimic the behaviour of the perylene terminal, it is possible to directly excite the latter and thereby bypass any EET events. Indeed, the absorption spectrum recorded for the dyad in dilute CHCl_3_ solution shows well-resolved transitions at *λ* > 400 nm which can be attributed entirely to the perylene-based chromophore (Fig. 7). There is a modest red shift of *ca.* 360 cm^−1^ for the 0,0 transition relative to pure perylene (Table 2), which can be explained in terms of increased conjugation with the appended carbonyl group. The molar absorption coefficient measured at the maximum amounts to 39 500 M^−1^ cm^−1^, which is similar to that reported^79^ for pure perylene. Fluorescence is readily detected following excitation at 420 nm and shows fine structure (Fig. 7). There is reasonable mirror symmetry with the corresponding absorption transition and a small Stokes shift (Table 2). The fluorescence quantum yield was determined to be 0.91 ± 0.05 in deoxygenated CHCl_3_, compared to a value of 0.87 for pure perylene under the same experimental conditions.

Time-resolved fluorescence studies with excitation at 440 nm allowed determination of the lifetime for the perylene-based excited-singlet state as being 6.2 ± 0.3 ns. Here, the decay profile gave a poor fit to a single-exponential process (*χ*^2^ = 1.45), as judged^70,71,80^ by the quality of the weighted residuals and their autocorrelation (Fig. S47‡). Again, a much improved fit (*χ*^2^ = 1.27) was obtained using a dual-exponential model, although the fit is still unsatisfactory (Fig. S48‡). An interesting result from this analysis is that there is a significant increase in the Stokes shift for the dyad relative to pure perylene (Table 2). This informs us that excitation of the perylene unit is followed by a some kind of geometrical relaxation in solution.

It is well known^81^ that intersystem crossing is quite inefficient for pure perylene, although the triplet state has been observed with an absorption maximum at *ca.* 515 nm and with a lifetime of *ca.* 4 ms.^81^ Laser excitation of the dyad at 420 nm, where only perylene absorbs, gave a weak transient absorption signal, centred at 520 nm (Fig. S41‡), which decayed *via* first-order kinetics with a lifetime of 690 μs in deoxygenated solution (Fig. S42‡). This signal is attributed to the perylene triplet-excited state. At higher laser intensities, the decay profile shows an increasing contribution from a second-order process occurring on short timescales which can be assigned to bimolecular triplet–triplet annihilation (Fig. S42‡). Indeed, both pure perylene and the perylene component of the dyad display P-type delayed fluorescence^76,81^ in deoxygenated fluid solution.1
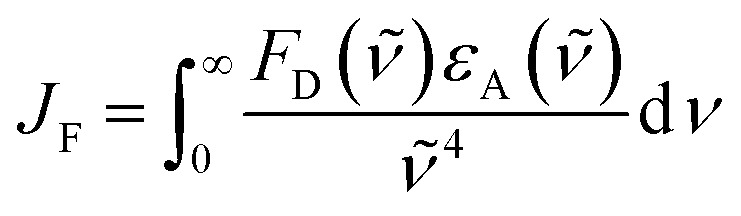
2
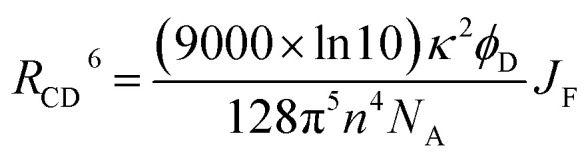


### Electronic energy transfer

There is a thermodynamic gradient amounting to *ca.* 3350 cm^−1^ that favours EET from the singlet-excited state of pyrene to perylene in PY-P_8_-PER. The spectral overlap integral (*J*_F_) accompanying EET was calculated^82^ from eqn (1) where *F*_D_ refers to the fluorescence spectrum for the pyrene donor recorded in wavenumbers and with the total intensity normalised to unity. The term *ε*_A_ refers to the molar absorption coefficient for the perylene acceptor with the integral being compiled between 350 and 550 nm (Fig. S44‡). The derived value, *J*_F_ = 3.0 × 10^−14^ mmol^−1^ cm^6^, is somewhat higher than found^50^ for related compounds, reflecting the minor spectral shifts and broadening. With this value, eqn (2) can be used to calculate the Förster critical distance^51^ (*R*_CD_) for random orientations (*κ*^2^ = 0.67) of the two chromophores. Here *ϕ*_D_ refers to the fluorescence quantum yield for the isolated donor, *n* is the refractive index of the surrounding medium and *N*_A_ is Avogadro's constant. The derived value is 25.4 ± 1.0 Å.

Comparison of absorption spectra recorded for PY-P_2_ and PY-P_8_-PER indicates that the pyrene unit can be preferentially, but not exclusively, excited at wavelengths between 310 and 360 nm. For example, pyrene accounts for *ca.* 70% of the excitation intensity at 350 nm. Emission spectra recorded for PY-P_8_-PER in dilute methanol solution, with excitation at 350 nm, show contributions from both fluorophores, although the relative yield for perylene emission far outweighs that for pyrene. This situation is fully consistent with EET along the molecular axis, as has been demonstrated^48–50^ for other molecular dyads bearing the same terminals. Several methods can be used to determine the probability (*P*_EET_) for intramolecular EET in this system, while working with dilute (<2 μM) solutions ensures the absence of complications from bimolecular EET.

**Fig. 8:**
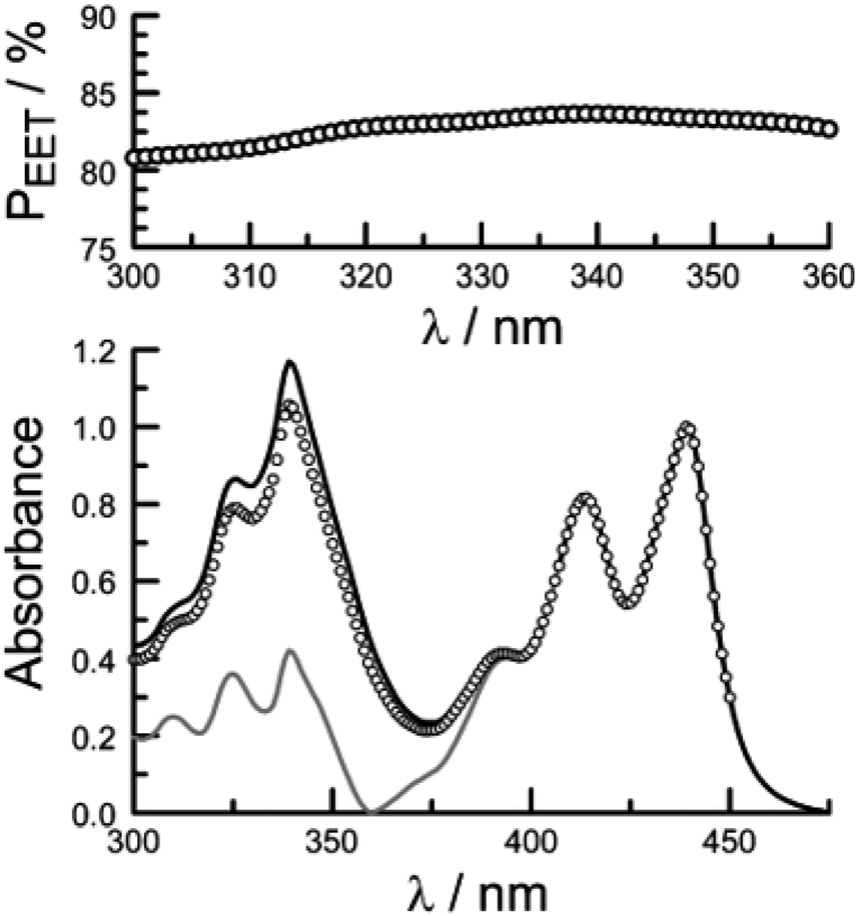
The lower panel shows a comparison of normalised absorption (black curve) and excitation (open circles) spectra recorded for PY-P_8_-PER in dilute chloroform solution. The fluorescence wavelength was 525 nm. The grey curve represents the absorption spectrum derived for the perylene component of the dyad. The upper panel shows the wavelength dependence for *P*_EET_ as measured by this procedure, the median value being 83%.

Firstly, excitation spectra are compared with absorption spectra recorded for PY-P_8_-PER in dilute solution.^83^ The excitation spectra were recorded by monitoring emission at 525 nm with a 1 nm slit. The absorption spectrum was recorded at the same resolution but it was still necessary to allow for a minor spectral shift to obtain accurate alignment of the peaks. The two spectra were normalised at the peak of the perylene absorption. At a qualitative level, it is apparent that photons absorbed by the pyrene chromophore lead to emission from the perylene unit since the excitation spectrum shows a clear peak centred around 340 nm. A quantitative comparison requires knowledge of the absorption spectrum for the perylene chromophore across the near-UV region. This was obtained by subtracting the spectrum for PY-P_2_ from that of PY-P_8_-PER recorded under identical conditions and adjusting to give a smooth profile. The perylene spectrum corresponds to the case where there is no EET while the absorption spectrum of the dyad corresponds to quantitative EET (Fig. 8). The excitation spectrum closely resembles the absorption spectrum of the dyad. At any given wavelength, *P*_EET_ can be obtained from eqn (3) where *A*_EX_(*λ*), *A*_DY_(*λ*) and *A*_PE_(*λ*) refer, respectively to the intensity of the excitation spectrum, the absorption spectrum measured for the dyad and the derived absorption spectrum for the perylene chromophore (Fig. 7). The mean *P*_EET_ obtained in his way for PY-P_8_-PER in CHCl_3_ is 83 ± 2%. Repeating the procedure for methanol (*P*_EET_ = 90 ± 4%), propan-1-ol (*P*_EET_ = 85 ± 4%) and diethylether (*P*_EET_ = 90 ± 6%) shows that the nature of the solvent has only a modest effect on the overall efficacy of the EET process.3
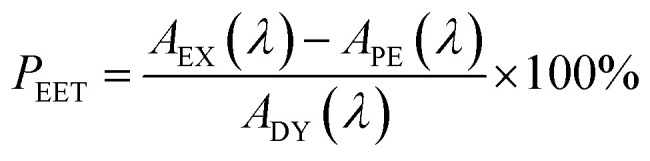


**Fig. 9:**
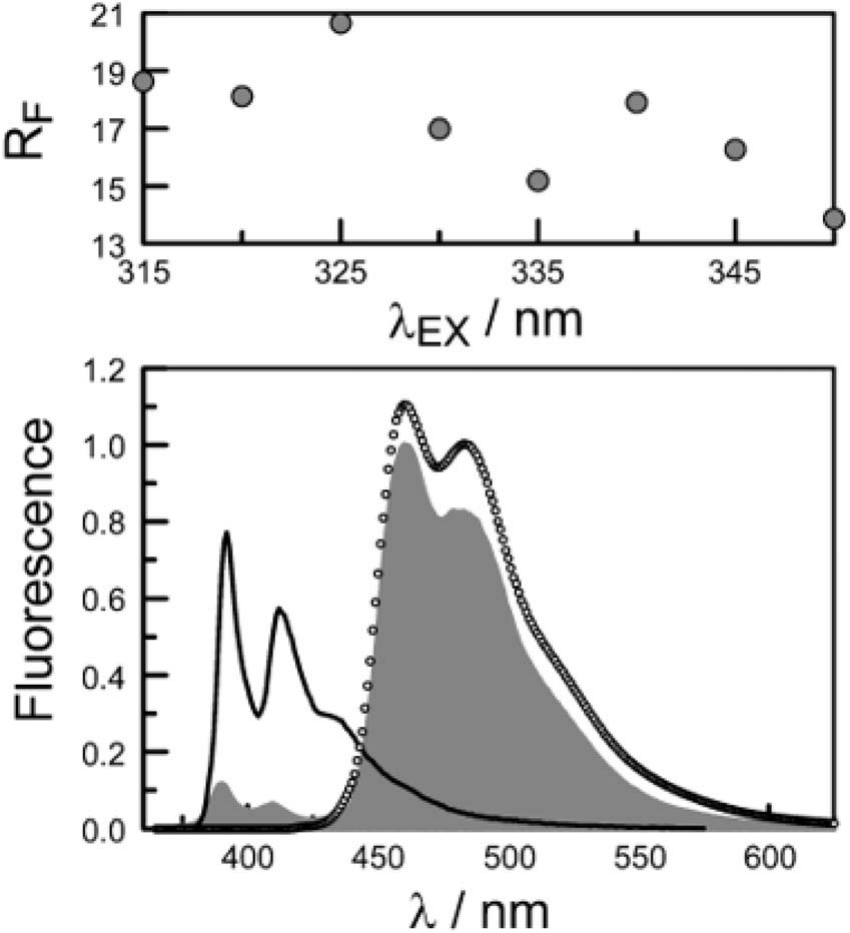
The lower panel compares the fluorescence spectrum recorded for PY-P_8_-PER in chloroform (shaded profile) following excitation at 350 nm with spectra projected for *P*_EET_ values of 0% (black curve) and 100% (open circles). The upper panel illustrates how the ratio of integrated emission profiles depends on excitation wavelength.

The second protocol used to determine *P*_EET_ involves measuring the ratio (*R*_F_) of fluorescence signals attributable to each of the terminals following preferential excitation into the pyrene chromophore. In this case, it is necessary to know the fraction (*α*) of excitation light absorbed by the pyrene chromophore. The respective emission signals are obtained by integration between 350 and 440 nm for pyrene and between 450 and 550 nm for perylene (Fig. 9). The required probability is obtained from eqn (4) where *ϕ*_D_ and *ϕ*_A_ refer, respectively, to the fluorescence quantum yields for the isolated pyrene donor and the isolated perylene acceptor. Using a few different excitation wavelengths, the mean *P*_EET_ derived for PY-P_8_-PER in chloroform was found to be 85 ± 4%. This is in excellent agreement with that obtained from the excitation spectrum. Similar values were obtained for methanol (*P*_EET_ = 89 ± 3%), propan-1-ol (*P*_EET_ = 84 ± 4%) and diethylether (*P*_EET_ = 88 ± 6%) solutions; note, in the latter case, the dyad shows limited solubility.4
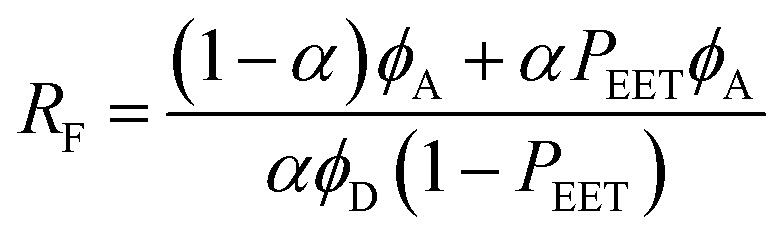


The derived *P*_EET_ values are insensitive to the polarity and/or proticity of the solvent. At first sight, this might appear to be unexpected since the global structure of polyproline residues is known to be dependent on the nature of the solvent. However, there are certain conditions associated^62–65^ with such effects, most notably the need for a sufficient number of unsubstituted proline residues in the chain,^53^ while the presence of bulky terminals might add a further barrier to internal rotation. The rate of intramolecular EET for a through-space mechanism is known^82^ to be highly sensitive to the separation distance and to the mutual orientation of the chromophores. Comparison of the derived *P*_EET_ values with the Förster critical distance indicates that the mean separation distance (*r*) between the terminals is *ca.* 19 Å (eqn (5)), assuming random orientation of the transition dipole moment vectors. Furthermore, the CD results (Fig. S50‡) indicate that an important fraction of the oligo-proline spacer adopts the PPII structure, which is believed^64,65^ to be relatively rigid in solution. This latter result is in line with the DFT calculations, which predict a major contribution from the all-*trans* chain. The calculations have not identified a significant fraction of the all-*cis* species and instead indicate that the *cis*-amide occurs only at a few sites along the chain. The net effect seems to be a restricted range of geometries. To examine this conclusion in more detail, time-resolved fluorescence studies were made with PY-P_8_-PER in solution.5
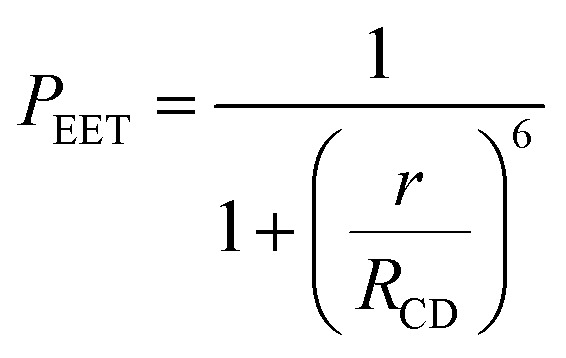


These latter measurements were made by time-correlated, single photon counting (TCSPC) with excitation at 330 nm. The temporal resolution of the set-up was *ca.* 300 ps after deconvolution of the instrument response function. Emission from pyrene could be isolated in the region of 410 nm while perylene fluorescence dominates the signal at longer (*λ* > 450 nm) wavelengths. Initial studies were made for PY-P_8_-PER in CH_3_OH solution, where the NMR studies predict a dominant role (*i.e.*, *ca.* 90%) for the *trans*-isomer; we emphasise here that reference to *cis* or *trans* isomers concerns only the amide bond connected directly to the pyrene chromophore unless stated otherwise.

Focussing on the pyrene-based donor, the decay profile was clearly non-exponential and therefore was treated initially as the sum of exponentials (eqn (6)). Here, *I*_F_ refers to the fluorescence intensity and *t* is the time delay from the start pulse. Analysis considers a series of *j* exponential decays, each with a fractional amplitude *A*_*j*_ and a lifetime *τ*_*j*_. The amplitude weighted lifetime (〈*τ*〉) can be obtained from eqn (7). Fitting the data to dual-exponential kinetics allowed estimation of 〈*τ*〉 as being 2.1 ± 0.2 ns, but the quality of the fit was poor (*χ*^2^ = 1.48; Fig. S69‡). The same analysis applied to emission from the perylene unit gave 〈*τ*〉 as being 7.40 ± 0.09 ns (*χ*^2^ = 1.44; Fig. S49‡). Apart from the limited statistical fit, a second issue with this analysis is that the two derived lifetimes are too similar for comfort (Table 3) and, in fact, the derived parameters were found to be sensitive to the total number of counts in the peak channel (CPC) (Fig. S69–S71‡).6
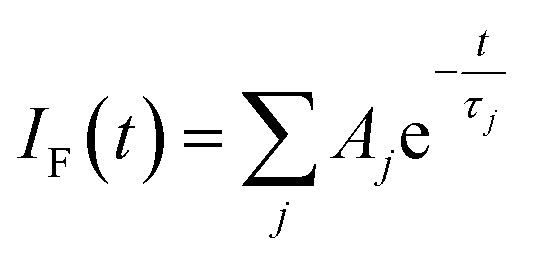
7
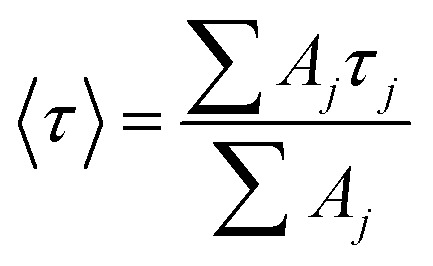


**Table 3:** Compilation of the excited-state lifetimes derived for the pyrene-based donor in PY-P_8_-PER and assigned to individual conformers in polar and weakly-polar solvents^a^

Species	Solvent	DE^b^	Restricted^c^	Initial^d^	Refined^e^
T1	CH_3_OH	1.6 (94%)	1.7 (90%)	1.7 (85%)	1.6 (81%)
T2		NA	NA	0.83 (5%)	0.8 (9%)
C1		9.8 (6%)	2.6 (10%)	2.6 (8%)	3.1 (2%)
C2/3		NA	NA	11.4 (2%)	9.0 (8%)
〈*τ*〉^f^		2.1	1.8	1.9	2.0
T1	CHCl_3_	2.0 (79%)	1.7 (60%)	1.7 (52%)	1.6 (51%)
T2		NA	NA	0.83 (8%)	0.8 (9%)
C1		11.5 (21%)	2.6 (40%)	2.6 (33%)	3.4 (5%)
C2/3		NA	NA	11.4 (7%)	7.0 (35%)
〈*τ*〉^f^		4.0	2.1	2.6	3.5

a All values are given in ns. The value given in parenthesis refers to the fractional contribution of that species.

b Fit to dual-exponential kinetics.

c Predicted values using only T1 and C1 to represent the molecular structure.

d Distributive model using four lifetimes but with fixed parameters as listed in Table S6.

e Distributive model using four lifetimes but allowing for stepwise optimisation.

f Mean lifetime calculated from eqn (7).

The same analysis made for PY-P_8_-PER in dilute CHCl_3_ solution, where the NMR studies indicate a mixture of *trans* and *cis* species with a slight preference for the *trans*-isomer, gave an unacceptable fit to dual-exponential kinetics (Fig. S72‡) and the derived lifetimes are again sensitive to the CPC (Fig. S72 & S73‡). In this case, the mean lifetime (〈*τ*〉 = 4.0 ± 0.18 ns) is longer than found for CH_3_OH, seemingly in agreement with the derived *P*_EET_ values. Adding a further exponential component serves no useful purpose other than to improve the quality of the statistical fit, especially since it would need to be of similar magnitude to those already used. This situation has been encountered before by other researchers, starting with Ware *et al.*^84^ in the 1970's, and has led to the development of system-specific models.^85^ In our case, it was considered that the system might be better treated in terms of the computed molecular structure, assuming the *cis*- and *trans*-isomers are non-interconverting species on this timescale. The NMR studies made in CH_3_OH indicate a preference for the all-*trans* conformation but there is more structural diversity in CHCl_3_. Consequently, the quantum chemical studies (DFT/B3LYP/6-311G(d,p)/PCM) were applied to CHCl_3_ solutions (see ESI‡).

In the first instance, we considered only the lowest-energy *cis*- and *trans*-isomers and used the computed structures to calculate expected lifetimes. Thus, the centre-to-centre separation distances (*r*) are 19.06 Å and 14.61 Å, respectively, for *trans*- and *cis*-structures while the FRET orientation factors (*κ*^2^) for the static structures are 1.15 ± 0.05 and 0.08 ± 0.03, respectively (Fig. S57, S61 & S62‡). Using this information, the rate constant (*k*_F_) for intramolecular EET can be calculated from eqn (8) for each of the two conformers (Table S6‡).8
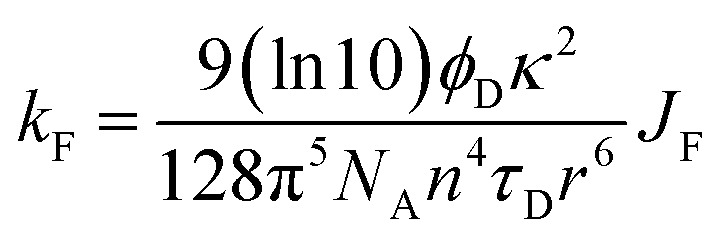


This simple analysis indicates that EET is less efficacious for the *cis*-isomer, despite the shorter *r*, because of the unfavourable orientation factor, but the actual disparity in *k*_F_ is modest. Now, we can use the NMR results to establish the ratio of isomers present in each solvent at equilibrium and thereby predict 〈*τ*〉 values. For methanol, where the TCSPC data give a 〈*τ*〉 value of 2.1 ± 0.2 ns and NMR estimates *K* ≈ 9, we predict 〈*τ*〉 as being 1.8 ± 0.2 ns (Table 3). For CHCl_3_ (*K* ≈ 1.5) we obtain a 〈*τ*〉 value of 2.1 ± 0.2 ns compared to a value of 4.0 ± 0.2 ns from the dual-exponential fits (Table 3). Overall, this level of agreement is reasonable, implying that the computed structures are realistic. However, attempts to simulate the experimental decay curves using only these two structures were far from convincing, especially at longer delay times. Clearly, more species are involved!

The structural calculations project a significant contribution from a conformer having a *cis*-amide at the second site and with all other amides in the *trans* geometry (Fig. S65‡). This species has a total energy only 1–2 kcal mol^−1^ higher than that of the lowest energy all-*trans* species (T1): we refer to this new conformer as T2. Interconversion between T1 and T2 requires *cis*–*trans* isomerisation and is expected to be relatively slow but, on energetic grounds, it seems likely that T2 could contribute up to 20% of the total *trans*-family. The main consequence of the *cis*-amide at the 2-site is to fold back the pyrene unit such that *r* is shortened to 14.7 Å but there is a corresponding decrease in *κ*^2^ to 0.33 (Fig. S65–S68‡). The net result is that EET for T2 is expected to be somewhat faster (*k*_F_ = 12 × 10^8^ s^−1^) than for either T1 or C1.

The DFT calculations project the possible involvement of two additional conformers having a *cis*-amide at the pyrene terminal (Table 3). Both species possess an all-*trans* oligo-proline chain but differ in the mutual arrangement of the polycycles. The first such structure (C2; Fig. S63‡), which is *ca.* 2 kcal mol^−1^ less favourable than C1, has *r* ≈ 25.6 Å and returns a *k*_F_ value of 0.88 × 10^8^ s^−1^ (Table 3). The second species (C3; Fig. S64‡) has *r* ≈ 23.1 Å and gives a *k*_F_ value of 0.55 × 10^8^ s^−1^. Since these latter *cis*-species give similar *k*_F_ values and are expected to be minor contributors in methanol, averaged values were used for the simulations.

**Scheme 2:**
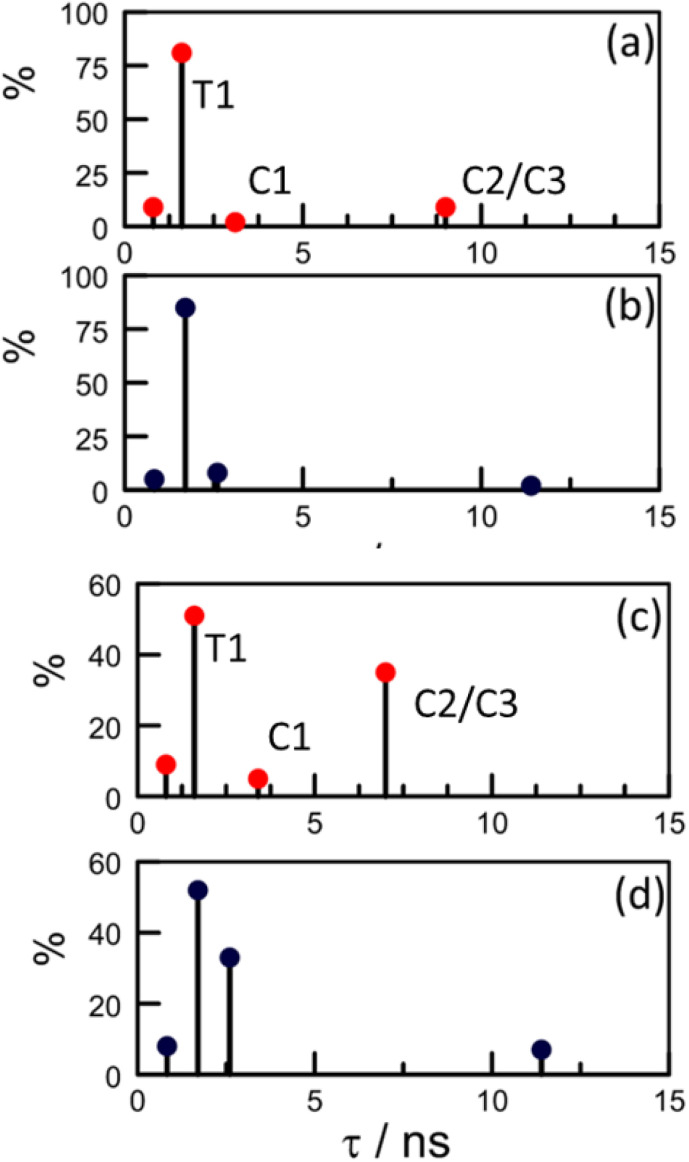
Stick diagrams illustrating how optimisation affects the global distribution of conformers as determined by the distributive model. The upper panels refer to (a) initial and (b) refined parameters for PY-P_8_-PER in methanol. The lower panels refer to (c) initial and (d) refined parameters in CHCl_3_. The labels on panels (a) and (c) identify the actual conformer.

Effectively, this approach doubles the number of parameters by which to simulate the experimental decay curves (Table S6‡), meaning that constraints have to be imposed. Thus, the structural landscape for PY-P_8_-PER in methanol is dominated by *trans* species. This is significant because the oligo-proline chain adopts the PPII structure, which is relatively stiff and might be expected to minimise structural fluctuations. The starting parameters for simulation of the pyrene emission decay kinetics retained the *k*_F_ values calculated from the structural information (Table 3) and imposed a total *trans* composition of 90%. The fractional contribution of T1 was held at 85% while C2/C3 was assigned a minor role (Table S6‡). The simulated profile was obtained by convolution of these four lifetimes with an experimental instrument response function.^84^ After the initial minimisation, optimisation allowed the sets of parameters to vary but retaining the ratio of *trans*- to *cis*-species (Fig. S74–S76‡). The net result is an excellent fit to the experimental data with the revised parameters shown in Scheme 2. The derived 〈*τ*〉 is 2.0 ± 0.2 ns, which can be compared to that extracted from dual-exponential fits, where 〈*τ*〉 was found to be 2.3 ± 0.24 ns (Table 3).

Our NMR data obtained for CHCl_3_ solutions infer a significantly increased population of *cis*-amides and this is reflected in the starting parameters for the kinetic simulations (Table S6‡). Unlike with CH_3_OH, optimisation leads to a marked change in the composition and, in particular, serves to minimise the contribution of C1. Now, the *cis*-family is dominated by the longer-lived C2/C3 species at the expense of C1 (Scheme 2). Interestingly, the impact of T2 remains similar to that deduced for CH_3_OH solution but the fractional contribution of T1 falls precipitously (Table 3 and Scheme 2). After optimisation, 〈*τ*〉 adopts a value of 3.5 ± 0.2 ns, compared to that derived from dual-exponential fits, where 〈*τ*〉 is 4.1 ± 0.18 ns.

In methanol, optimisation has little effect on the lifetimes of individual species and the most significant change is the increased contribution from C2/C3 at the expense of C1 (Scheme 2). The fit is somewhat insensitive to the ratio of the *trans*-species such that the final composition is not particularly well defined. In CHCl_3_, the lifetime of C2/C3 is roughly half that predicted from the DFT structures while its relative concentration is amplified by a factor of 5-fold. The effect of moving from methanol to chloroform can be rationalised in terms of T1 being transformed into C2/C3, which would only require isomerisation at the pyrene terminal. This seems to be the only viable isomerisation available to PY-P_8_-PER in solution at ambient temperature.

There is a considerable gradient favouring triplet–triplet EET from pyrene to perylene, although such processes usually occur *via* an electron exchange mechanism^86^ requiring either orbital contact between the reactants or short-range super-exchange interactions.^87^ To eliminate the possibility of closely interacting pairs of reactants, we carried out a series of transient absorption spectral studies using 4 ns laser excitation at 340 nm. Under such conditions, with PY-P_8_-PER dissolved in deaerated methanol, the pyrene triplet-excited state could be detected through the appearance of the characteristic absorption band centred at 425 nm. This signal decayed *via* first-order kinetics with a lifetime of 415 ± 25 μs to restore the prepulse baseline (Fig. S43‡). No other transient species could be detected on the μs timescale and there is no corresponding signal in the region of 520 nm that could safely be attributed to the perylene triplet. Thus, triplet–triplet energy transfer does not occur within this system. This finding is fully consistent with a situation where the terminals are kept spatially isolated by the oligo-proline connector.

## Conclusions

Our long-term ambition is to construct artificial light-harvesting antennae^88^ based on polyproline spacer units. These spacers need to provide structural integrity without affecting the opto-electronic properties of the light-active components or interfering with the EET events. Both the latter requisites are met by the proline octamer studied here. Furthermore, the mean *P*_EET_ and *R*_CD_ values can be tuned over a modest range by changing the nature of the chromophores. The main challenge, however, is controlling the rigidity of the spacer, especially in the vicinity of the chromophores. This situation is exacerbated by the observed stabilisation of the *cis*-isomer with weakly polar solvents such as CHCl_3_. On the other hand, the all-*trans* geometry (T1) abounds in methanol solution and the structure can be accurately modelled by DFT calculations. Here, the level of agreement between experimental and calculated EET parameters is extremely good for separation distances in the region of 20 Å. We have observed that the all-*trans* chain can be perturbed by inclusion of a *cis*-amide at either the 1 (*i.e.*, terminal pyrene connection; C2 and C3) or 2-sites (T2). These latter conformers make a relatively minor contribution to the total population in polar solvents but provide for disparate rates of EET. Their involvement is problematic in terms of establishing a molecular ruler. However, by way of an entirely fortuitous situation, they tend to cancel out each other by returning an average lifetime not too dissimilar to that of the all-*trans* species. Their presence is noted only in terms of the quality of the statistical fit of the kinetic data.

Our calculations do not assign a role for the corresponding all-*cis* species and instead the maximum contribution for *cis*-geometries amounts to three out of nine amides. This arrangement (C1) provides a favourable architecture for fast EET by bringing the terminals into relatively close proximity. During optimisation of the distributive model, the composition of the *cis*-family shifts in favour of those conformers having a single *cis*-amide at the pyrene connection. This allows the “tail” of the decay function to be modelled more accurately since these mono-*cis* structures are less amenable for intramolecular EET. In fact, the geometry of the pyrene-based amide has a significant effect on the rate of ETT and, as such, the use of polyproline spacers^45,47^ to validate “molecular rulers” is predicated on the availability of the all-*trans* chain.

This investigation introduces a new type of system-specific model by which to interpret the EET parameters. The strength of the model is the integration of NMR structural information with quantum chemical calculations. Rather than attempt to compute molecular structures in different solvents, we have relied on calculations made in CHCl_3_ where earlier studies have obtained good agreement between crystal structures and computed geometries for bis-proline residues.^88^ Refinement of the data analysis allows the composition to vary but retains the same structures. For this situation to hold, the overall geometry must be quite stiff and this is likely to be true, at least for methanol solution where the *trans* geometry abounds and the CD spectrum indicates helicity.

## Data availability

Full details concerning synthesis and characterisation of new materials, including X-ray crystallography, are provided as part of the ESI‡ package. Steady-state and time-resolved fluorescence data are also given in the ESI,‡ together with examples of the transient absorption spectral studies. Details on the computational studies, including cartesian coordinates for key structures, are contained within the ESI.‡ Further information regarding data analysis is available from the corresponding author upon reasonable request.

## Author contributions

This work forms part of the Doctoral Thesis for SMAW, who performed most of the experimental work under the joint supervision of ACB and AH. ACB designed the NMR studies and was instrumental in subsequent data analysis. SMAW designed the optical spectroscopic studies and performed the relevant experiments. AH developed the distributive model and carried out the simulations. AH wrote the first version of the manuscript. All authors contributed towards preparation of subsequent drafts of the manuscript. All authors have given approval to the final version of the manuscript.

## Conflicts of interest

There are no conflicts to declare.

## Supplementary Material

SC-015-D3SC05287G-s001

SC-015-D3SC05287G-s002
