# Supplementary material for: Deducing the conformational space for an octa-proline helix
Source: Chem Sci. 2023 Dec 21;15(5):1657–71. doi: 10.1039/d3sc05287g (PMC10829019; doi:10.1039/d3sc05287g)
Supplement: SC-015-D3SC05287G-s001 [file SC-015-D3SC05287G-s001.pdf]

Electronic Supplementary Material (ESI) for Chemical Science.
This journal is © The Royal Society of Chemistry 2023

Deducing the Conformational Space for an Octa-Proline Helix

Sara M. A. Waly, Andrew C. Benniston and Anthony Harriman

Molecular Photonics Laboratory, Bedson Building, School of Natural and Environmental Sciences, Newcastle University, Newcastle upon Tyne, NE1 7RU, U.K.

SUPPORTING INFORMATION

Total number of pages: 81

CONTENTS

S1	Synthesis and compound characterisation	S2
S2	Crystal structure determination of **Py-P$_2$**	S25
S3	Further spectroscopic information	S27
S4	Oligoproline helicity probed by circular dichroism	S42
S5	FRET orientation factor	S44
S6	Computational studies	S46
S7	Time-resolved fluorescence studies with the dyad	S68
S8	Simulation of TCSPC decays using a distributive model	S73
S9	References	S80

S1. Synthesis and compound characterisation

Materials and methods

All chemicals and reagents were purchased from commercial suppliers and were used as received without further purification. 1-Aminopyrene, Boc Pro Pro-OH, perylene-3-carboxylic acid, DIPEA, 4 M HCl/1,4 dioxane, COMU and dry DMF were purchased from Sigma Aldrich. All reactions were performed in dried glassware under an atmosphere of nitrogen using Schlenk techniques. Dichloromethane was distilled over calcium hydride. ^{1}H, ^{13}C{^{1}H}, ^{19}F{^{1}H}, ^{11}B{^{1}H} NMR spectra and 2D NMR spectra were recorded on JEOL ECS 400, Bruker Avance 300, Jeol Lambda 500, 300, 400 or Bruker Avance 700 MHz instruments and referenced to the residual proton signals of the solvent. Mass spectrometry analysis was performed using Electrospray Ionization and obtained from the EPSRC-sponsored Mass Spectrometry Service at Swansea Medical School. UV-Vis spectra were obtained using a UV-1800 Shimadzu UV spectrophotometer scanning from 300 –700 nm. Circular dichroism spectra were measured in a high precision quartz SUPRASIL (Hellma Analytics®) cell with a light path of 0.1 mm. Data was acquired using a JASCO J-810 spectropolarimeter under nitrogen (5 - 10 ml/min) connected to a PTC-423S temperature controller maintaining 25°C using a Julabo water bath.

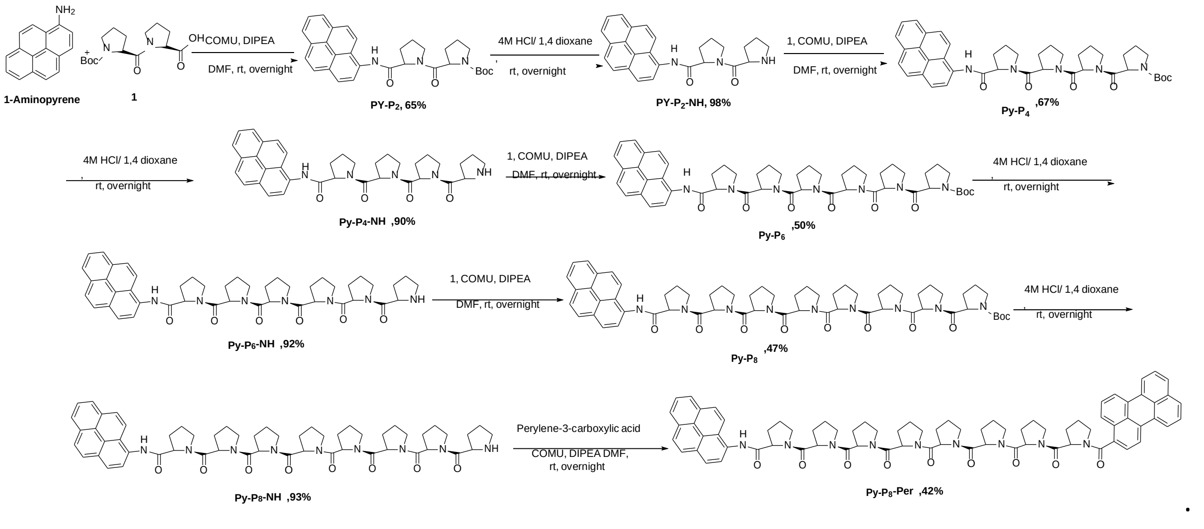

Scheme S1. Full synthetic procedures used in the preparation of the octamer **PY-P$_8$-PER**.

Preparation of the pyrene control PY-P₂

To a 50 mL Schlenk flask, under a nitrogen atmosphere, compound **1** (0.1 g, 0.32 mmol, 1 eq) was dissolved in dry DMF (20 mL). Keep the reaction stirring for 10 mins under nitrogen, then add *N,N*-diisopropylethylamine (9.6 μl, 0.64 mmol, 2eq) followed by COMU (180 mg, 0.64 mmol, 2eq). The reaction mixture was stirred for 5 mins then 1-aminopyrene (69 mg, 0.32 mmol, 1eq) was added. The reaction mixture stirred at room temperature for 12 h. After the reaction complete, the solvent was distilled and the crude was dissolved in dichloromethane and washed with 0.1M HCl, $NaHCO_3$, brine and water and the solvent was evaporated under vacuo. The resulting residue was purified by silica gel column chromatography using DCM: MeOH (8:2, V/V). Compound **PY-P$_2$** was isolated as white ppt (50 mg, 65%). **R$_f$**: 0.82 (CH_2Cl_2: MeOH, 8:2). **Mp**: 122-124°C. **^{1}H NMR** (700 MHz, $CDCl_3$) δ = 1.47-1.52 (2s, 9H, CH_3 (Boc), rotamers), 1.80-1.88 (m, 2H, H-cyclopentane), 2.06-2.22 (m, 5H, H-cyclopentane), 2.57-2.71 (m, 1H, H-cyclopentane), 3.46-3.57 (m, 2H, H-cyclopentane), 3.66-3.84 (m, 2H, H-cyclopentane), 4.40-4.60 (m, 1H, H-cyclopentane), 5.05- 5.13 (m, 1H, H-cyclopentane), 7.89-7.92 (d, 2H, *J* =7.1 Hz, ArH), 7.95-8.00 (d, 2H, *J* = 8.1 Hz, ArH), 8.03 -8.10 (d, 3H, *J* = 20.2 Hz, ArH), 8.18-8.23 (d, 1H, *J* = 16.5 Hz, ArH), 8.49-8.62 (d, 1H, *J* =36.9 Hz, ArH), 10.20-10.27 (br., 1H, NH). **^{13}C-NMR** (75.5 MHz, $CDCl_3$) δ = 23.7, 24.3, 25.5, 26.7, 28.5, 29.6, 31.5, 46.8, 47, 36.6, 47.4, 50.6, 57.9, 60.8, 62.9, 80.1, 120.5, 120.9, 121.1, 122.7, 124.7, 125, 125.1, 126, 126.5, 127.2, 127.5, 127.7, 128.6, 130.8, 131.2, 131.3, 154.7, 170.2, 173.9. **HRMS**: (ASAP$^+$) calcd. for $C_{31}H_{33}N_3O_4$ $[M]^+$: 511.25, found $[M]^+$ 511.25, $[M+Na]^+$ 534.23, $[M+NH_4]^+$529.28.

Preparation of PY-P₂-NH

Compound **PY-P$_2$** (100 mg, 0.2 mmol, 1 eq.) was treated with 4M HCl in 1,4-dioxane (6 ml) and the reaction was stirred overnight at room temperature. After the reaction finish, the volatiles were evaporated under reduced pressure to give a yellow solid. Saturated $NaHCO_3$ was added to neutralize the solution. Then CH_2Cl_2 was added and the organic layer was separated and concentrated to afford the desired product **PY-P$_2$-NH** as a yellow solid (98 mg, 98%). **R$_f$**: 0.25 (CH_2Cl_2/CH_3OH = 1:1). **Mp**. 129-130°C. **^{1}H NMR** (400 MHz, $CDCl_3$) δ = 1.67-2.20 (m, 8H, H-cyclopentane), 3.27 (m, 4H, H-cyclopentane), 4.35-4.46 (m, 1H, H-cyclopentane), 5.21-5.23 (m, 1H, H-cyclopentane), 7.87-7.90 (d, 3H, *J* = 4.5 Hz, ArH), 7.94-8.06 (m, 4H, *J* = 4.5 Hz, ArH), 8.18-8.20 (d, 1H, *J* = 8.8 Hz, ArH), 8.31-8.34 (d, 1H, *J* = 9.2 Hz, ArH), 10.31 (br., 1H, NH), 10.99 (br., 1H, NH). **^{13}C-NMR** (75.5 MHz, $CDCl_3$) δ = 24.5, 29.3, 31.5, 36.4, 46.9, 58.4, 61.4,

65.8, 67, 122, 122.4, 124.1, 124.3, 124.9, 125.1, 126, 126.8, 127, 127.4, 129, 130.5, 130.7, 131, 167.22, 170.97. **IR** (neat): v_{max}/cm^{-1} 3300 (N-H, w), 1527 (N-O, s). **HRMS**: (ASAP$^+$) calcd.for $C_{26}H_{25}N_3O_2$ $[M]^+$: 411.19, found $[M]^+$ 411.20, $[M+H]^+$ 412.20.

Preparation of PY-P$_4$

To a 50 mL Schlenk flask, under a nitrogen atmosphere, compound **1** (0.1 g, 0.32 mmol, 1 eq.) was dissolved in dry DMF (20 mL). Keep the reaction stirring for 10 mins under nitrogen, after that add *N,N*-diisopropylethylamine (9.6 *µl*, 0.64 mmol, 2eq) followed by COMU (180 mg, 0.64 mmol, 2eq). The reaction mixture was stirred for 5 mins then **PY-P$_2$-NH** (131mg, 0.32 mmol, 1eq) was added. The reaction mixture stirred at room temperature for 12 h. After the reaction finish, the solvent was distilled and the crude was dissolved in dichloromethane and washed with 0.1M HCl, $NaHCO_3$, brine and water and the solvent was evaporated under vacuo. The resulting residue was purified by silica gel column chromatography using DCM: MeOH (7:3, V/V). Compound **PY-P$_4$** was isolated as yellow ppt (67 mg, 67%). **R$_f$**: 0.72 (CH_2Cl_2: MeOH, 8:2). **Mp**: 126-128°C. **^{1}H NMR** (700 MHz, $CDCl_3$) δ = 1.36-1.42 (2s, 9H, CH_3 (Boc), rotamers), 1.97-2.10 (m, 16H, H-cyclopentane), 3.47-3.73 (m, 8H, H-cyclopentane), 4.45-4.48 (m, 1H, H-cyclopentane), 4.68-4.71 (m, 2H, H-cyclopentane), 4.97-4.99 (m, 1H, H-cyclopentane), 7.87-7.88 (d, 2H, *J* = 4.5 Hz, ArH), 7.90-7.93(d, 2H, *J* = 8.8 Hz, ArH), 8.01-8.06(m, 3H, ArH), 8.11-8.14(d, 1H, *J* = 9.4 Hz, ArH), 8.48-8.51(d, 1H, *J* = 9.4 Hz, ArH), 10.04-10.6 (m, 1H, NH), 10.64-10.67 (m, 1H, NH). **^{13}C-NMR** (75.5 MHz, $CDCl_3$) δ = 23.7, 24.3, 24.6, 25.1, 25.3, 25.4, 25.5, 27.3, 28, 28.2, 28.3, 28.4, 28.6, 29.1, 47.1, 47.3, 47.5, 47.7, 57.9, 58.4, 60.9, 80.4, 120.7, 121.5, 123, 124.6, 124.8, 125, 125.1, 125.2, 125.9, 126.1, 126.6, 127.2, 127.6, 128.8, 130.7, 131.3, 155, 170.4, 171.4, 172.2, 172.8. **HRMS**: (ASAP$^+$) calcd. for $C_{46}H_{54}N_6O_7$ $[M]^+$: 802.39, found $[M]^+$ 802.39, $[M+NH_4]^+$820.43, $[M+Na]^+$ 825.39.

Preparation of PY-P$_4$-NH

Compound **PY-P$_4$** (70mg, 0.1 mmol, 1 eq.) was treated with 4M HCl in 1,4-dioxane (6 ml) and the reaction stirred overnight at room temperature under nitrogen. The volatiles were evaporated under reduced pressure to give a yellow solid. Saturated $NaHCO_3$ was added to neutralize the solution. Then CH_2Cl_2 was added and the organic layer was separated and concentrated to afford the desired product **PY-P$_4$-NH** as a yellow solid (98 mg, 98%). **R$_f$**: 0.25 (CH_2Cl_2/CH_3OH = 1:1). **Mp**: 129-130°C. **^{1}H NMR** (700 MHz, $CDCl_3$) δ = 1.95-2.18 (m, 16H, H-cyclopentane), 3.56-3.71 (m, 8H, H-cyclopentane), 4.54-4.58 (m, 1H, H-cyclopentane), 4.69-

4.74 (m, 2H, H-cyclopentane), 4.98-4.99 (m, 1H, H-cyclopentane), 7.95-7.96 (d, 4H, *J* = 4.5 Hz, ArH), 8.09-8.10 (d, 4H, *J* = 9.4 Hz, ArH), 8.34-8.38(d, 1H, *J* = 9.4 Hz, ArH) 9.72 (br., 1H, NH), 10.43 (br., 1H, NH). **^{13}C-NMR** (75.5 MHz, $CDCl_3$) δ =22.6, 24.7, 24.9, 25.3, 27.9, 28.3, 28.8, 29.6, 31.5, 47.3, 47.4, 47.6, 48.1, 58.5, 59.4, 60.8, 61.5, 120.8, 121.8, 123.2, 124.6, 125, 125.1, 125.3, 125.8, 126.1, 126.7, 127.2, 127.6, 128.9, 130.6, 130.7,131.2, 166.8, 170.3, 171.5, 172.2. **IR** (neat): v_{max}/cm^{-1} 2920 (C-H, w), 1527 (N-O, s). **HRMS**: ($ASAP^+$) calcd.for $C_{36}H_{39}N_5O_4$ $[M]^+$: 605.30, found $[M]^+$ 605.30, $[M+H]^+$ 606.30, $[M+Na]^+$628.28.

Preparation of PY-P$_6$

To a 50 mL Schlenk flask, under a nitrogen atmosphere, compound **1** (0.1 g, 0.32 mmol, 1eq) was dissolved in dry DMF (20 mL). Keep the reaction stirring for 10 mins under nitrogen, then add N,N-diisopropylethylamine (9.6 *μl*, 0.64 mmol, 2eq) followed by COMU (180 mg, 0.64 mmol, 2eq). The reaction mixture was stirred for 5 mins after that **PY-P$_4$-NH** (193mg, 0.32 mmol, 1eq) was added. The reaction mixture stirred at room temperature for 12 h. After the reaction finish, the solvent was distilled and the crude was dissolved in dichloromethane and washed with 0.1M HCl, $NaHCO_3$, brine and water. The solvent was evaporated under vacuo. The resulting residue was purified by silica gel column chromatography using DCM: MeOH (6:4, V/V). Compound **PY-P$_6$** was isolated as yellow ppt (146 mg, 50%), **R_f**: 0.72 (CH_2Cl_2: MeOH, 6:4). **Mp**: 129-131°C. **^{1}H NMR** (400 MHz, $CDCl_3$) δ = 1.38-1.45 (2s, 9H, CH_3 (Boc), rotamers), 1.93-2.10 (m, 24H, H-cyclopentane), 3.42-3.76 (m, 12H, H-cyclopentane), 4.29-4.34 (m, 1H, H-cyclopentane), 4.47-4.53 (m, 1H, H-cyclopentane), 4.61-4.71 (m, 3H, H-cyclopentane), 4.98-5.00 (m, 1H, H-cyclopentane), 7.89-8.16 (d, 9H, *J* = 9.4 Hz, ArH), 10.06-10.06 (br., 1H, NH), 10.66 (br., 1H, NH).**^{13}C-NMR** (75.5 MHz, $CDCl_3$) δ = 22.2, 23.5, 23.9, 24.1, 24.2, 24.6, 25.2, 25.5, 27.1, 27.8, 28.2, 28.3, 28.4, 28.9, 29.8, 31.2, 46, 46.5, 46.6, 46.8, 47.2, 50.1, 57.4, 57.7, 57.8, 58.8, 60.5, 61.8, 79.3, 120.8, 122.2, 124.5, 124.8, 125, 125.3,125.9,126.3, 126.6, 127.1, 128.2, 129, 130.5, 130.6,130.9, 131.3, 154.5, 169.6, 170.2, 170.3, 170.7, 171.2, 172.3. **HRMS**: ($ASAP^+$) calcd. for $C_{51}H_{61}N_7O_8[M]^+$: 899.46, found $[M]^+$899.46, $[M+H]^+$ 900.46, $[M+Na]^+$ 922.44 and $[M+NH_4]^+$917.49.

Preparation of PY-P$_6$-NH

Compound **PY-P$_6$** (90mg, 0.1 mmol, 1 eq.) was treated with 4M HCl in 1,4-dioxane (5 ml) and the reaction stirred overnight at room temperature. The volatiles were evaporated under reduced pressure to give a yellow solid. Saturated $NaHCO_3$ was added to neutralize the

solution. CH_2Cl_2 was added and the organic layer was separated and concentrated to afford the desired product **PY-P$_6$-NH** as a yellow solid (88 mg, 92%). **R$_f$**: 0.20 (CH_2Cl_2/CH_3OH = 1:1). Mp: 132-134°C. **^{1}H NMR** (500 MHz, MeOD) δ =1.61-1.80 (m, 24H, H-cyclopentane), 2.99-3.25 (m, 12H, H-cyclopentane), 4.01-4.42 (m, 6H, H-cyclopentane), 7.58-7.88 (m, 9H, ArH), 9.79 (br., 1H, NH), 10.33 (br., 1H, NH).**^{13}C NMR** (75.5 MHz, MeOD) δ = 24.9, 25.61, 25.66, 25.71, 25.76, 26.1, 28.8, 29.1, 29.3, 30.6, 30.8, 43.8, 47.5, 47.6, 48.2, 59.4, 59.5, 59.7, 60, 61.9, 64.2, 67.9, 72.2, 73.3, 123,124.6, 125.3, 125.8, 125.9,126.1, 126.2, 126.3, 127.3, 128, 128.1, 128.5, 130.6, 131.7, 132, 132.3, 167.7, 171.2, 171.7, 171.9, 172.6, 173.7. **HRMS**: (ASAP$^+$) calcd. for $C_{46}H_{53}N_7O_6$ $[M]^+$: 799.41, found $[M]^+$799.41, $[M+H]^+$800.41.

Preparation of PY-P$_8$

To a 50 mL Schlenk flask, under a nitrogen atmosphere, compound **1** (0.1 g, 0.32 mmol, 1 eq.) was dissolved in dry DMF (20 mL). Keep the reaction stirring for 10 mins, then add N,N-diisopropylethylamine (9.6 *µl*, 0.64 mmol, 2eq) followed by COMU (180 mg, 0.64 mmol, 2eq). The reaction mixture was stirred for 5 mins after that **PY-P$_6$-NH** (256mg, 0.32mmol, 1eq) was added. The reaction mixture stirred at room temperature for 12 h. After the reaction finish, the solvent was distilled and the crude was dissolved in dichloromethane and washed with 0.1M HCl, $NaHCO_3$, brine and water. The solvent was evaporated under vacuo. The resulting residue was purified by silica gel column chromatography using DCM: MeOH (6:4, V/V). Compound **PY-P$_8$** was isolated as yellow ppt. (120 mg, 47%), **R$_f$** : 0.72 (CH_2Cl_2: MeOH, 6:4). **Mp**: 129-131°C. **^{1}H NMR** (300 MHz, $CDCl_3$) δ = 1.32-1.38 (2s, 9H, CH_3 (Boc), rotamers), 1.84-2.07 (m, 32H, H-cyclopentane), 3.29-3.72 (m, 16H, H-cyclopentane), 4.24-4.25 (m, 1H, H-cyclopentane), 4.30-4.39 (m, 1H, H-cyclopentane), 4.55-4.69 (m, 5H, H-cyclopentane), 4.94-5.00 (m, 1H, H-cyclopentane), 7.86-7.90 (m, 4H, ArH), 7.97-8.05 (m, 4H, ArH), 8.11-8.14 (d, 1H, *J* = 9.4 Hz, ArH), 10.04 (br. 1H, NH), 10.59 (br. 1H, NH).**^{13}C-NMR** (75.5 MHz, $CDCl_3$) δ = 22.2, 22.6, 23.5, 24.1, 24.2, 24.4, 24.6, 24.8, 25.3, 26.7, 27.1, 27.5, 27.8, 28, 28.3, 28.5, 29, 29.9, 30.2, 31.2, 46, 46.5, 46.6, 46.8, 47.2, 56.9, 57.4, 57.6, 57.8, 58.8, 60.5, 61.8, 65.7, 79.2, 120.5, 122, 122.4, 124, 124.5, 124.6, 125.1, 125.9, 126.3, 126.6, 127.1, 128.2, 128.9, 130.7, 131, 131.2, 131.5, 154.5, 169.4, 169.6, 169.9, 170, 170.2, 170.6, 171.2, 172.5. **HRMS**: (ASAP$^+$) calcd. for $C_{61}H_{75}N_9O_{10}$ $[M]^+$: 1093.56, found $[M]^+$ 1093.56, $[M+H]^+$1094.56, $[M+Na]^+$1116.55 and $[M+NH_4]^+$1111.59.

Preparation of PY-P_8-NH

Compound **PY-P_8** (109 mg, 0.1 mmol, 1 eq.) was treated with 4M HCl in 1,4-dioxane (5 ml) and the reaction stirred overnight at room temperature. The volatiles were evaporated under reduced pressure to give a yellow solid. Saturated $NaHCO_3$ was added to neutralize the solution. Then CH_2Cl_2 was added and the organic layer was separated and concentrated to afford the desired product **PY-P_8-NH** as a yellow solid (95 mg, 93%). **R_f**: 0.20 (CH_2Cl_2/CH_3OH = 1:1). **Mp**: 134-135°C. **^{1}H NMR** (500 MHz, MeOD) δ = 1.82-2.02 (m, 32H, H-cyclopentane), 3.37-3.54 (m, 16H, H-cyclopentane), 4.39- 4.47 (m, 8H, H-cyclopentane), 7.82-8.03 (d, 9H, *J* = 9.4 Hz, ArH). **IR** (neat): v_{max}/cm^{-1} 2920 (C-H, w), 1527 (N-O, s). **HRMS**: (ASAP$^+$) calcd. for $C_{56}H_{67}N_9O_8$[M]$^+$: 993.51, found [M]$^+$993.51, [M+H]$^+$994.51 and[M+Na]$^+$ 1016.51.

Preparation of PY-P_8-PER

To a 50 mL Schlenk flask, under a nitrogen atmosphere, compound perylene-3-carboxylic acid (94 mg, 0.32 mmol, 1 eq.) was dissolved in dry DMF (20 mL). Keep the reaction stirring for 10 mins under nitrogen, then add *N,N*-diisopropylethylamine (9.6 *µl*, 0.64 mmol, 2eq) followed by COMU (180 mg, 0.64 mmol, 2eq). The reaction mixture was stirred for 5 mins after that add **PY-P_8-NH** (317mg, 0.32mmol, 1eq). The reaction mixture stirred at room temperature for 12 h. After the reaction finish, the solvent was distilled and the crude was dissolved in dichloromethane and washed with 0.1M HCl, $NaHCO_3$, brine and water. The solvent was evaporated under vacuo. The resulting residue was purified by silica gel column chromatography using DCM: MeOH (6:4, V/V). Compound **PY-P_8-PER** was isolated as yellow ppt. (149 mg, 42%), **R_f**: 0.32 (CH_2Cl_2: MeOH, 1:1). **Mp**: 123-125°C. **^{1}H NMR** (700MHz, $CDCl_3$) δ = 1.99-2.11 (m, 32H, H-cyclopentane), 3.42-3.74 (m, 16H, H-cyclopentane), 4.72-4.76 (m, 8H, H-cyclopentane), 7.40-7.51 (m, 4H, ArH), 7.60-7.62 (d, 2H, *J* = 8 Hz, ArH), 7.92-7.95 (d, 5H, *J* = 4.7 Hz, ArH), 8.07-8.09 (d, 9H, *J* = 6.3 Hz, ArH), 10.10 (br., 1H, NH), 10.65 (br., 1H, NH).**^{13}C-NMR** (75.5 MHz, $CDCl_3$) δ = 22.2, 22.7, 24.6, 24.7, 24.9, 25.4, 26.5, 27.9, 28.3, 28.4, 28.9, 30.3, 31.3, 36.4, 38.3, 46, 46.1, 46.2, 46.6, 46.8, 46.9, 47.1, 47.2, 49.2, 57.4, 57.9, 58, 58.1, 58.9, 60.6, 61.9, 66.6, 119.4, 120.4, 120.6, 122.1, 122.5, 124.1, 124.6, 124.7, 125.1, 125.4, 126, 126.1, 126.3, 126.5, 126.6, 126.7, 126.8, 127.1, 127.2, 127.9, 128.2, 128.3, 128.4, 128.6, 129, 130.5, 130.8, 130.9, 131, 131.2, 131.3, 131.5, 132.2, 134.5, 162.5, 169, 169.6, 169.8, 170.13,

170.17, 170.6, 171.3, 172.9. **HRMS**: ($ASAP^+$) calcd. for $C_{77}H_{77}N_9O_9$[M]+: 1271.58, found $[M]^+$ 1271.58, $[M+H]^+$ 1272.59, $[M+Na]^+$ 1295.57 and $[M+NH_4]^+$ 1289.61.

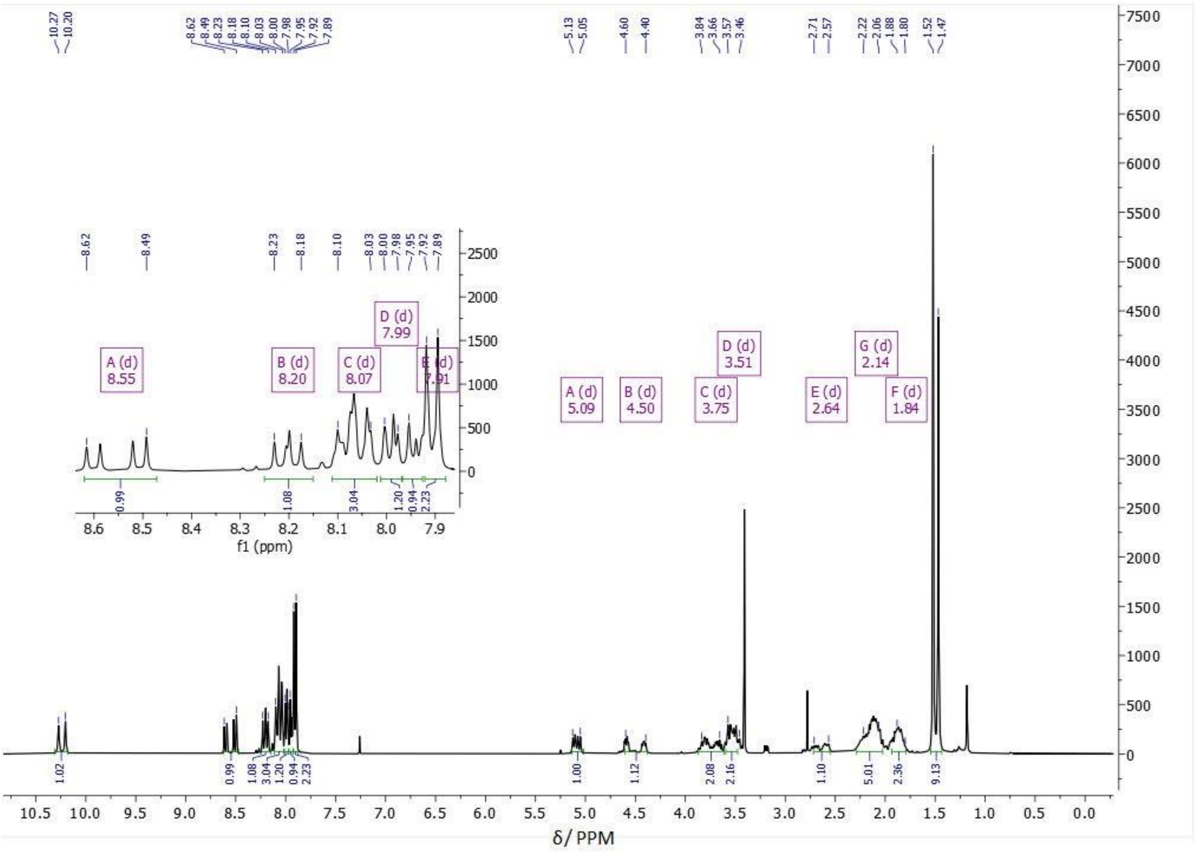

Figure S1. ^{1}H NMR (700 MHz, $CDCl_3$) spectrum recorded for compound **PY-P$_2$**.

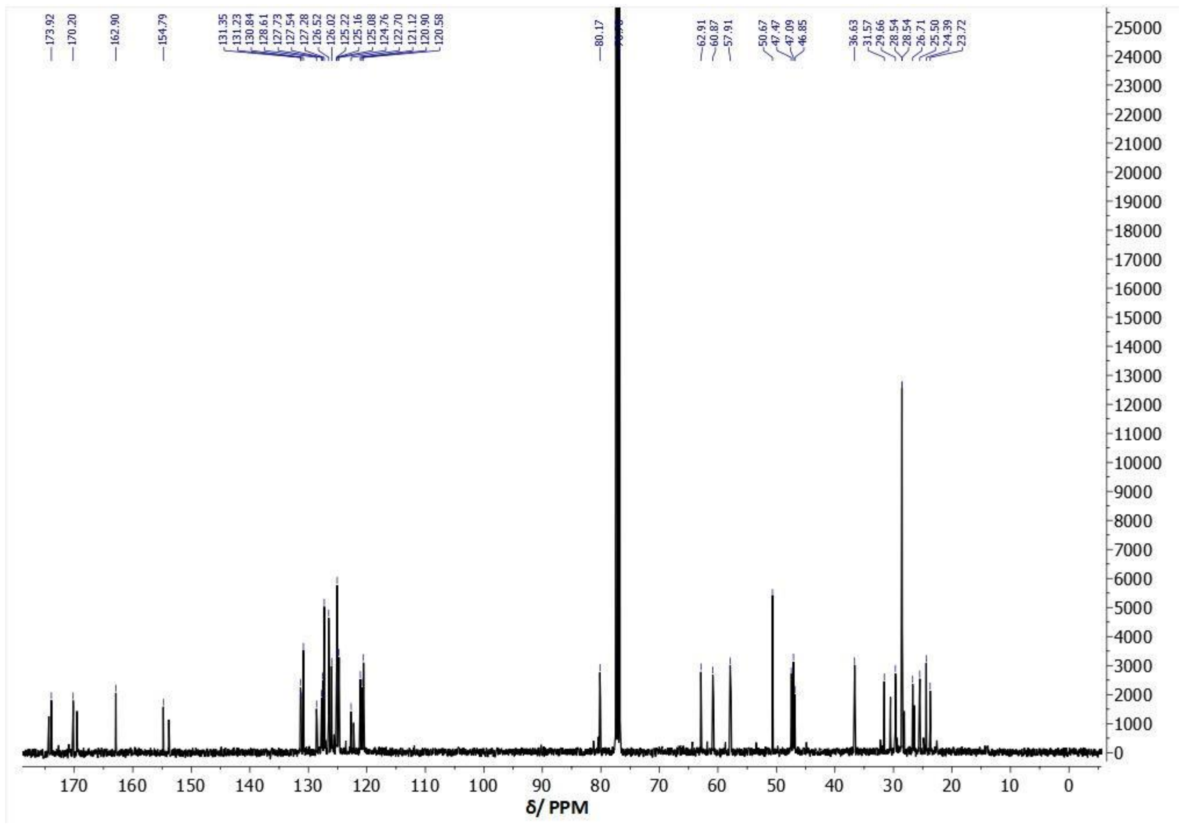

Figure S2. ^{13}C NMR (75 MHz, $CDCl_3$) spectrum recorded for compound **PY-P$_2$**.

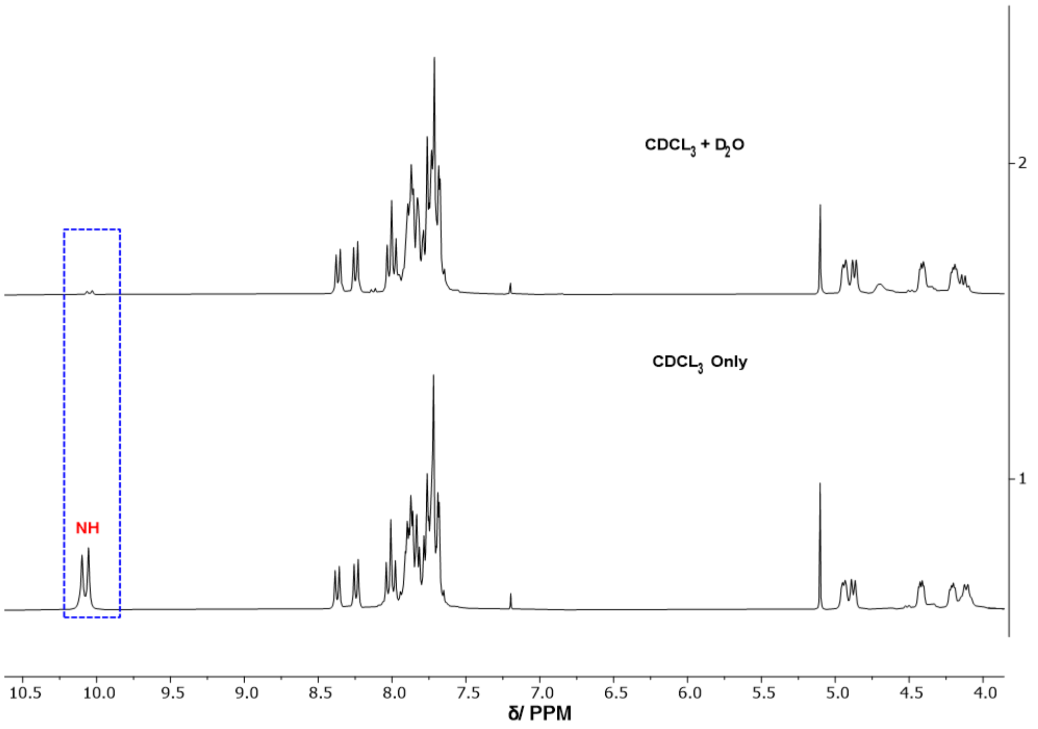

Figure S3. ^{1}H NMR (700 MHz) spectrum recorded for compound **PY-P$_2$** in $CDCl_3 + D_2O$ (above) and in $CDCl_3$ (below).

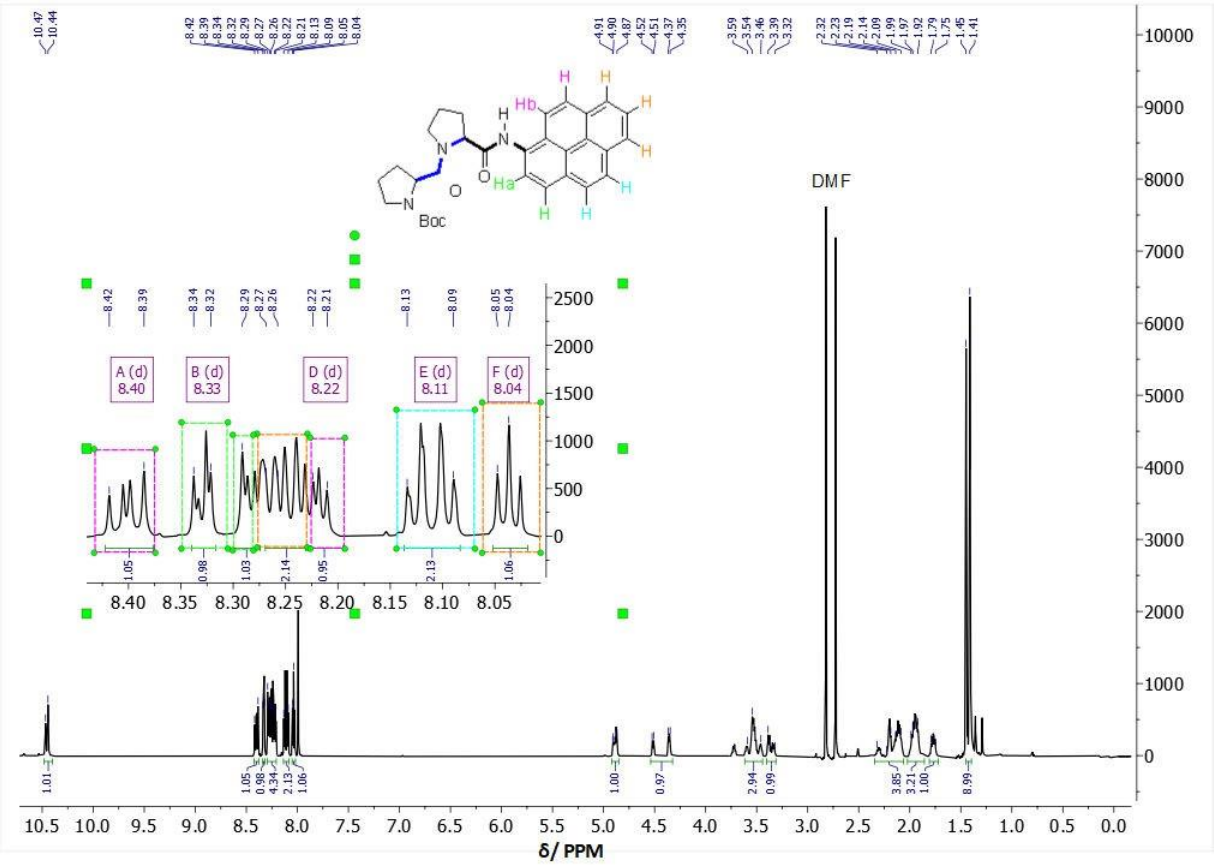

Figure S4. ^{1}H NMR (700 MHz, D6-DMSO) spectrum recorded for compound **PY-P$_2$**.

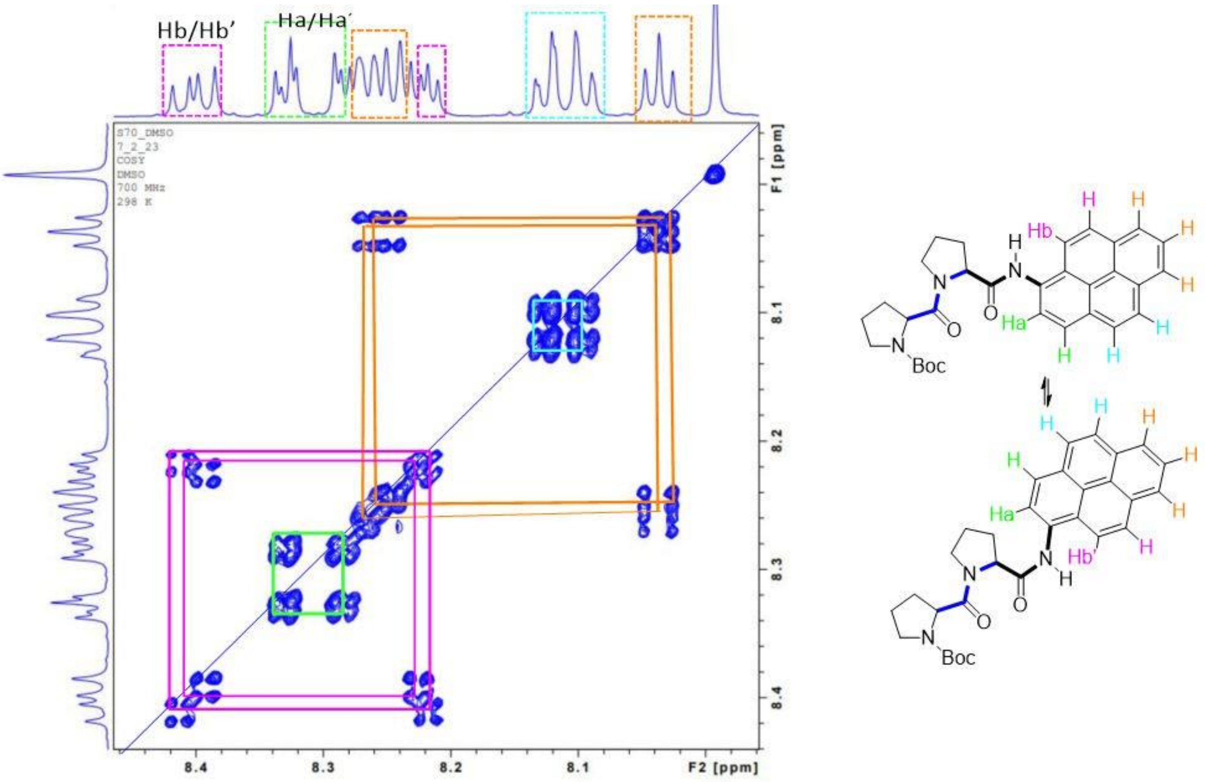

Figure S5. COSY NMR (700 MHz, D6-DMSO) spectrum recorded for compound **PY-P$_2$**.

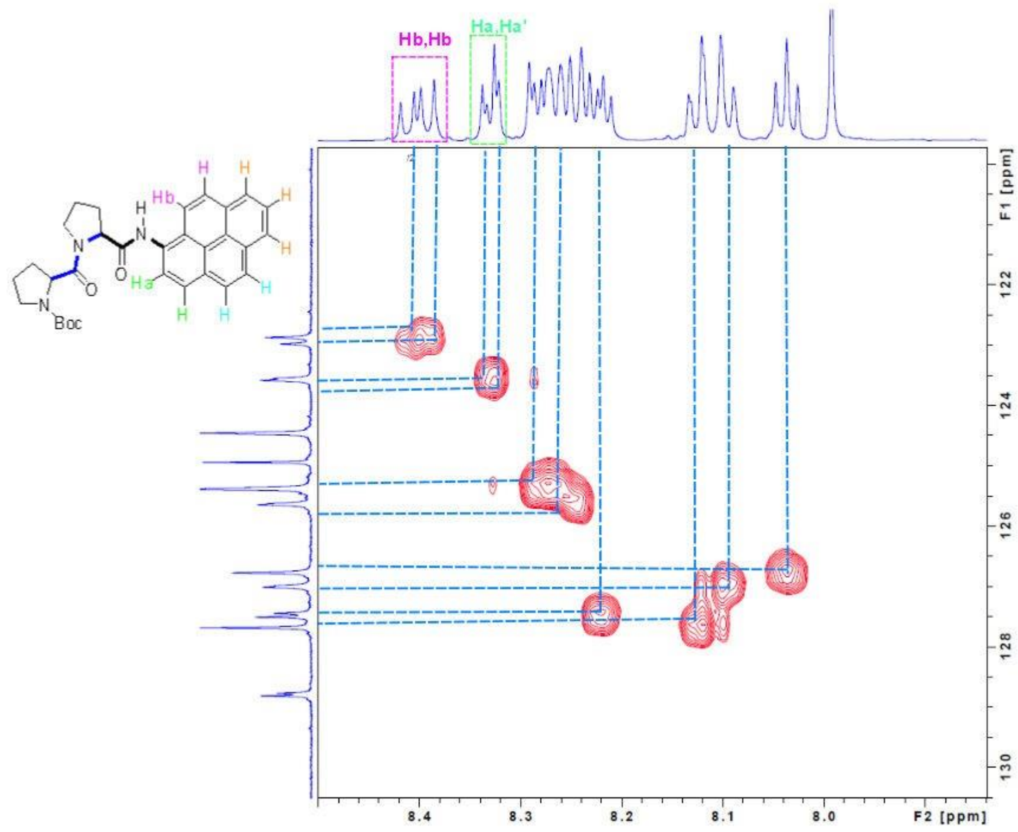

Figure S6. HSQC NMR (700 MHz, D6-DMSO) spectrum recorded for compound **PY-P$_2$**.

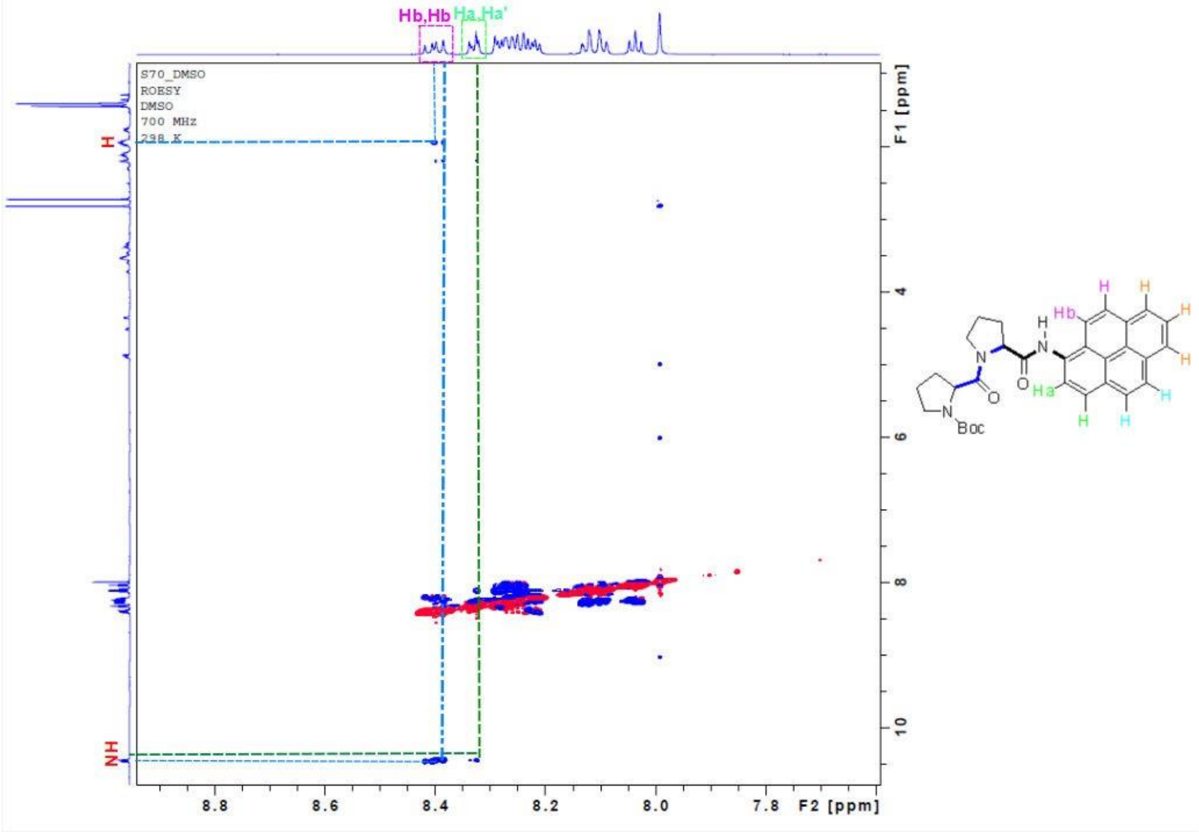

Figure S7. ROESY NMR (700 MHz, D6-DMSO) spectrum recorded for compound **PY-P$_2$**.

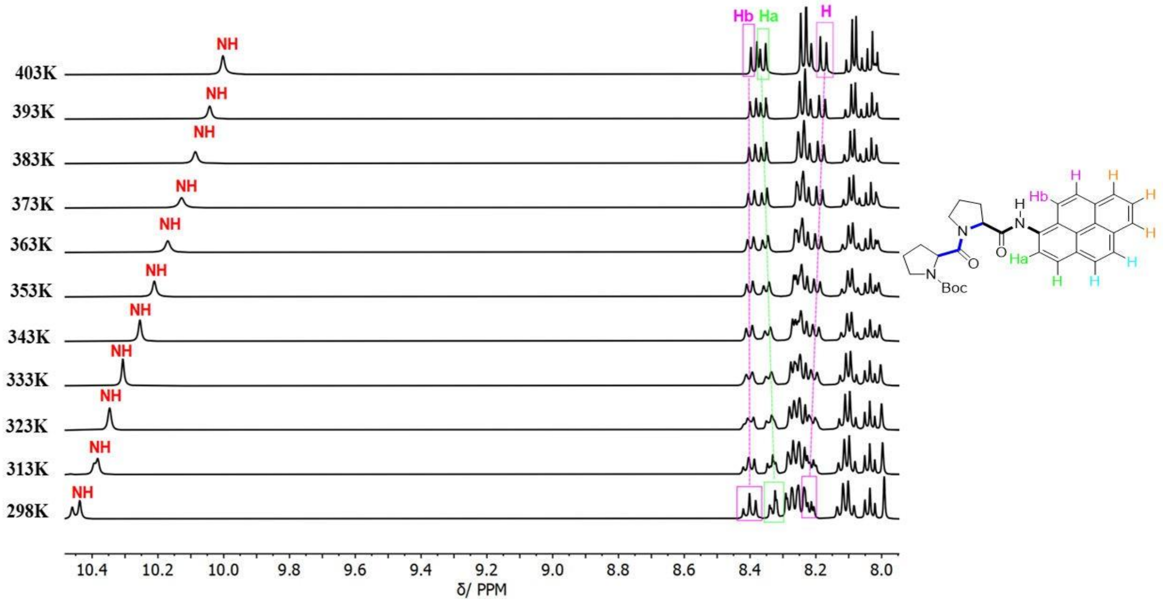

Figure S8. Variable temperature ^{1}H NMR spectra of **PY-P$_2$** in D_6-DMSO showing the aromatic region and the most downfield proton resonances.

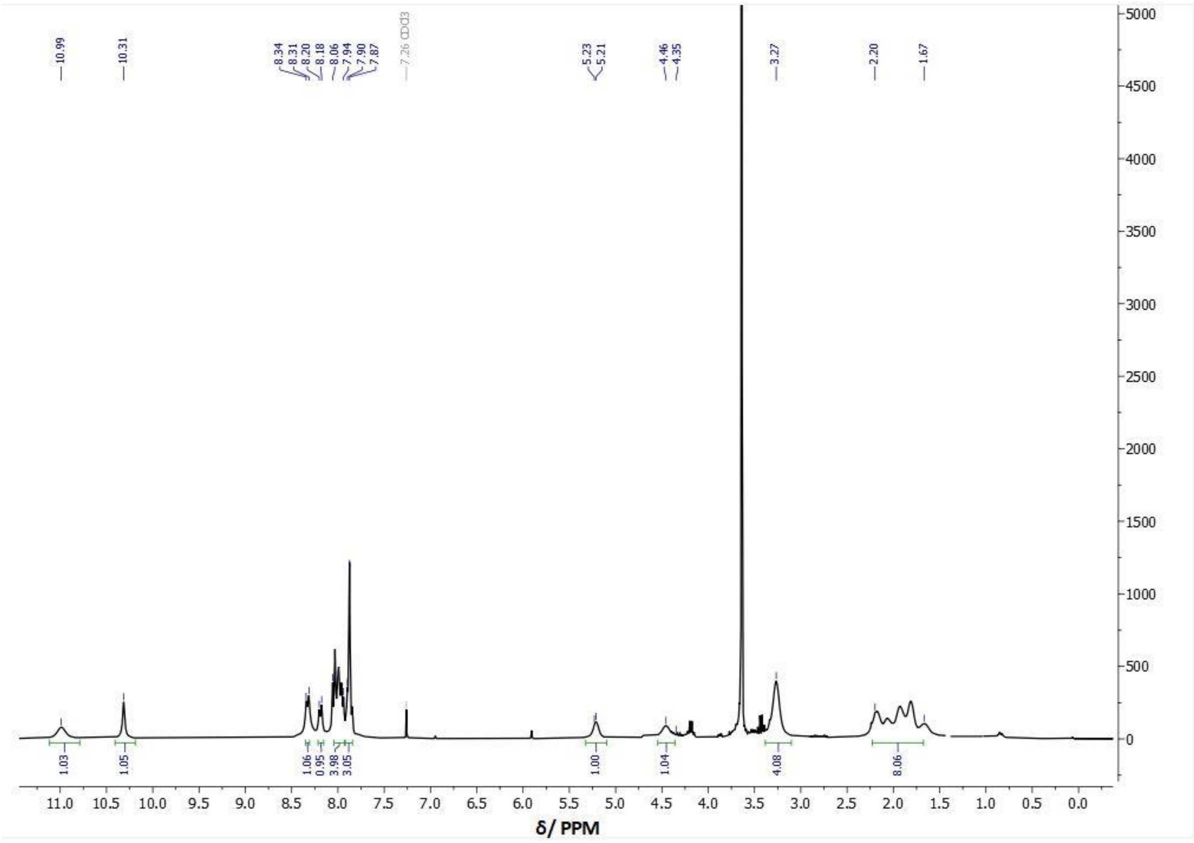

Figure S9. ^{1}H NMR (400 MHz, $CDCl_3$) spectrum recorded for compound **PY-P$_2$-NH**.

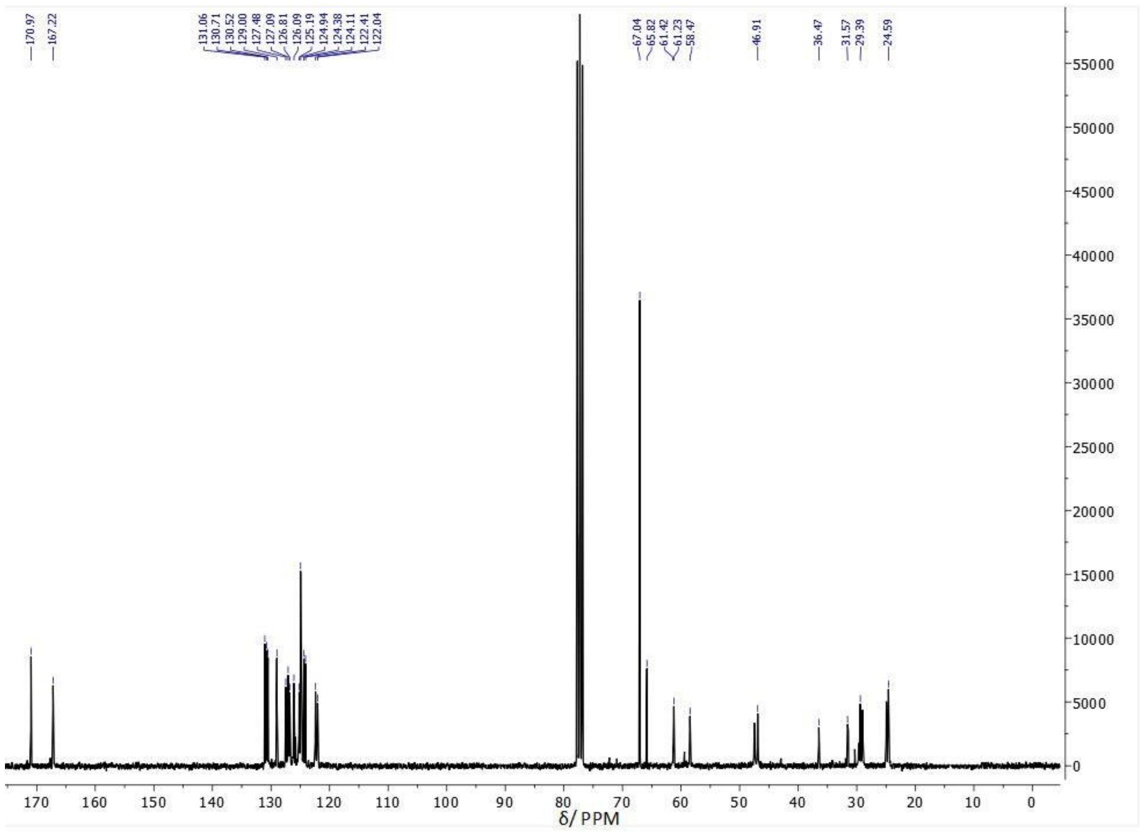

Figure S10. ^{13}C NMR (75 MHz, $CDCl_3$) spectrum recorded for compound **PY-P$_2$-NH**.

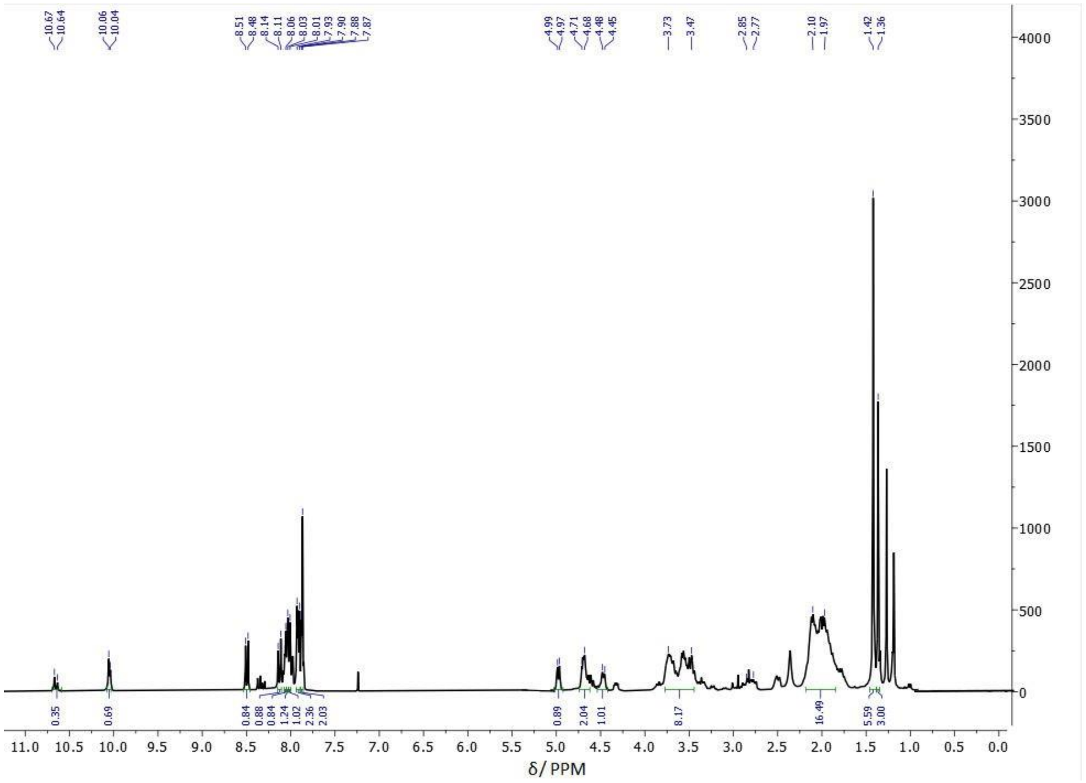

Figure S11. ^{1}H NMR (700 MHz, $CDCl_3$) spectrum recorded for compound **PY-P$_4$**.

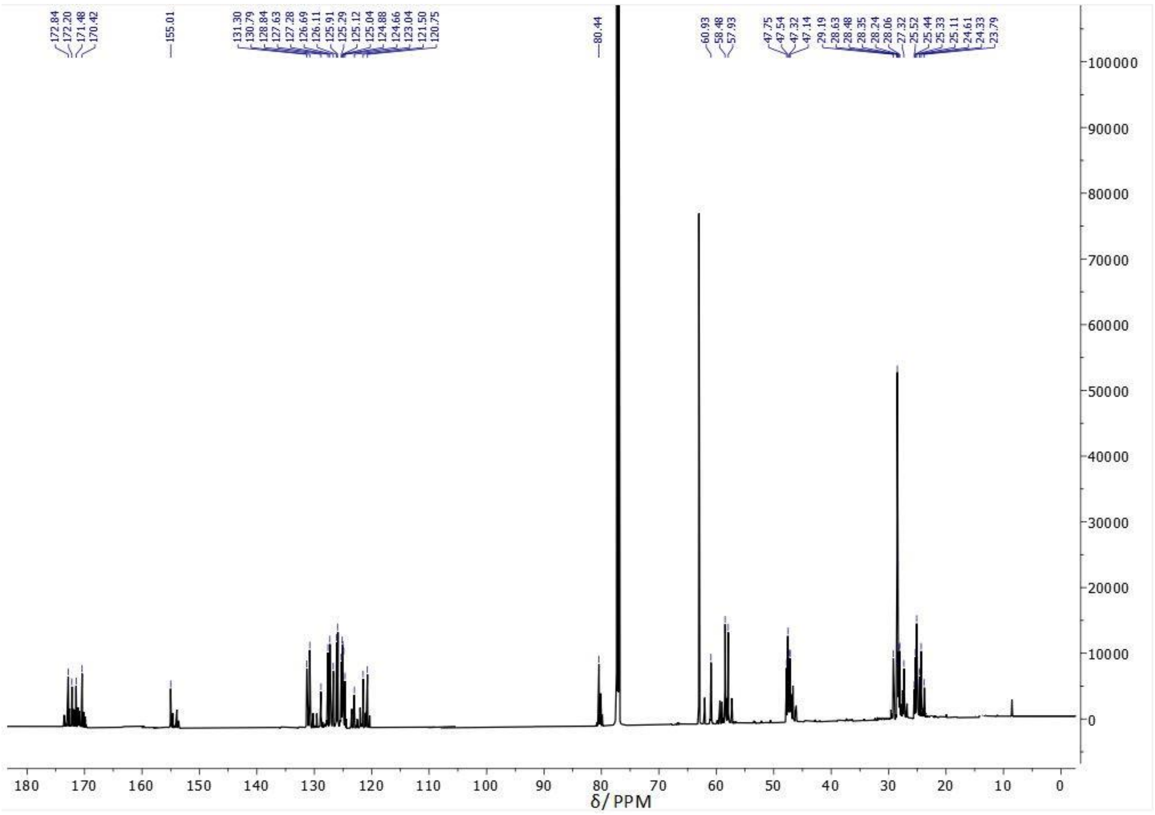

Figure S12. ^{13}C NMR (75 MHz, $CDCl_3$) spectrum recorded for compound **PY-P$_4$**.

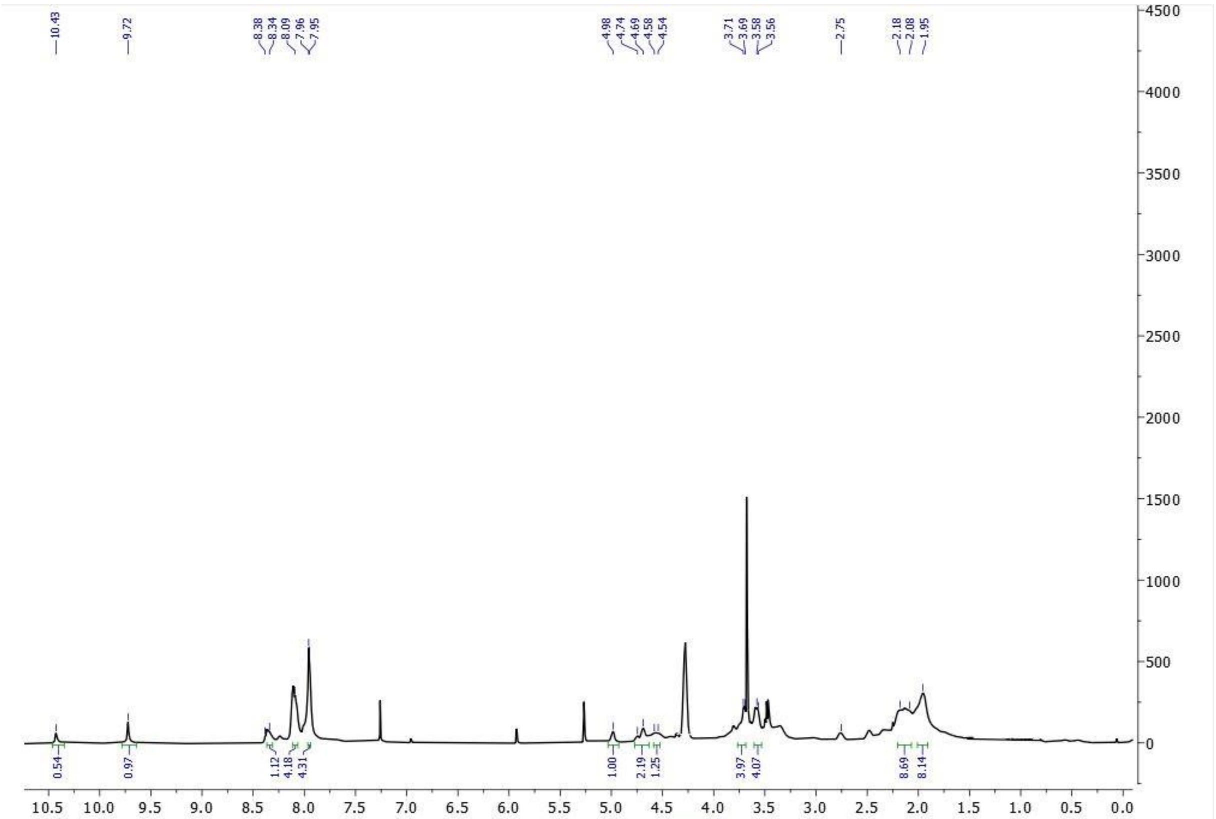

Figure S13. ^{1}H NMR (700 MHz, $CDCl_3$) spectrum recorded for compound **PY-P$_4$-NH**.

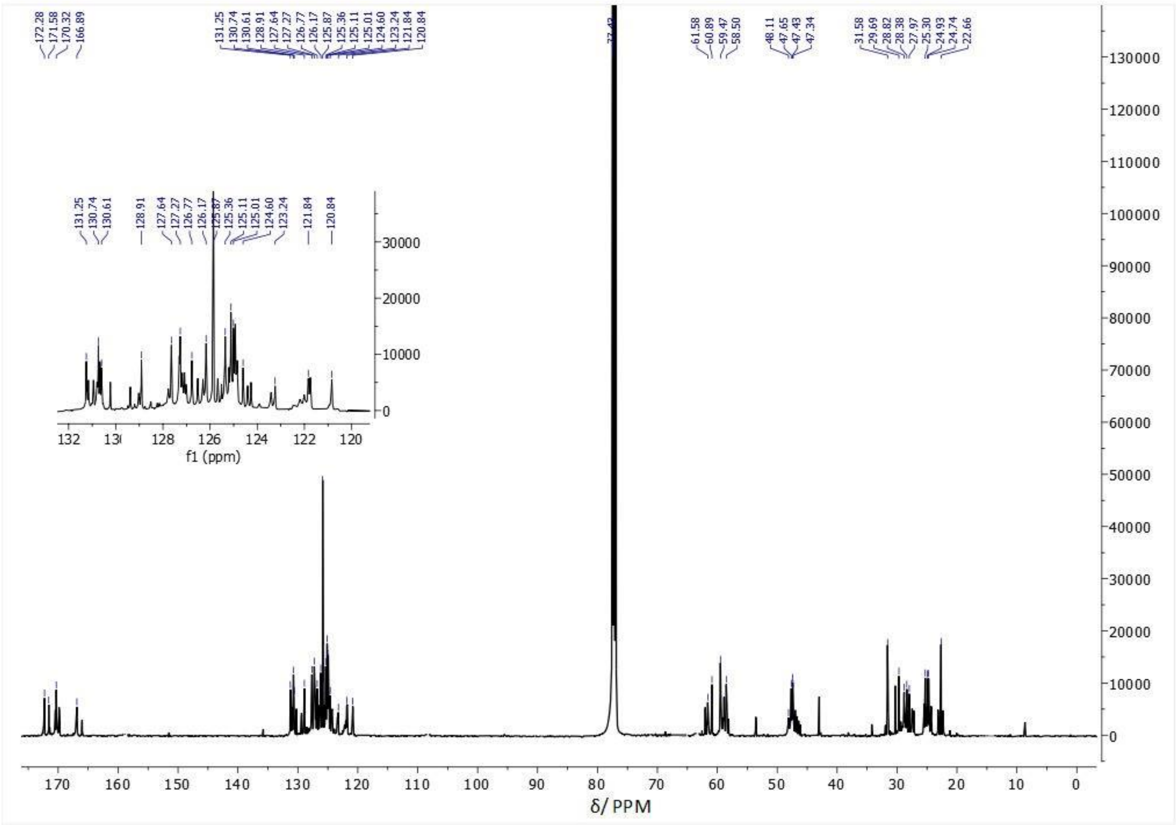

Figure S14. ^{13}C NMR (75 MHz, $CDCl_3$) spectrum recorded for compound **PY-P$_4$-NH**

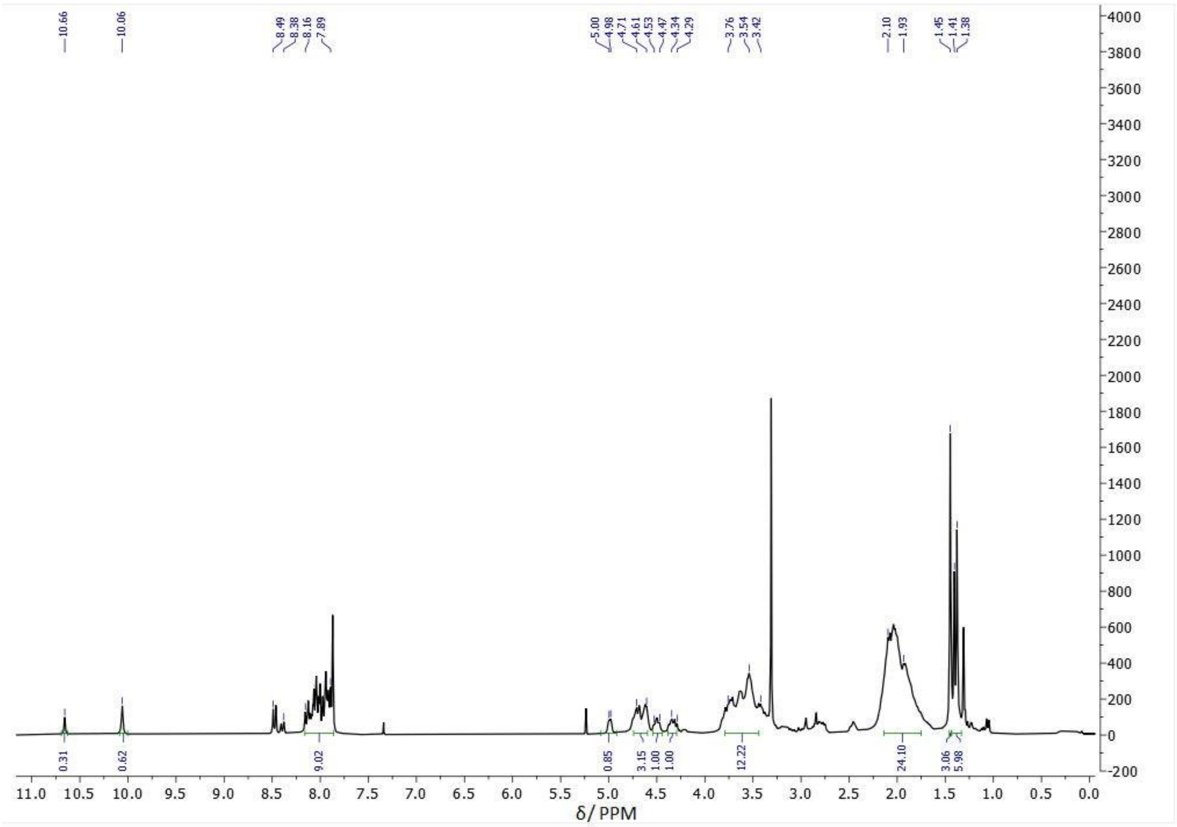

Figure S15. ^{1}H NMR (400 MHz, $CDCl_3$) spectrum recorded for compound **PY-P$_6$.**

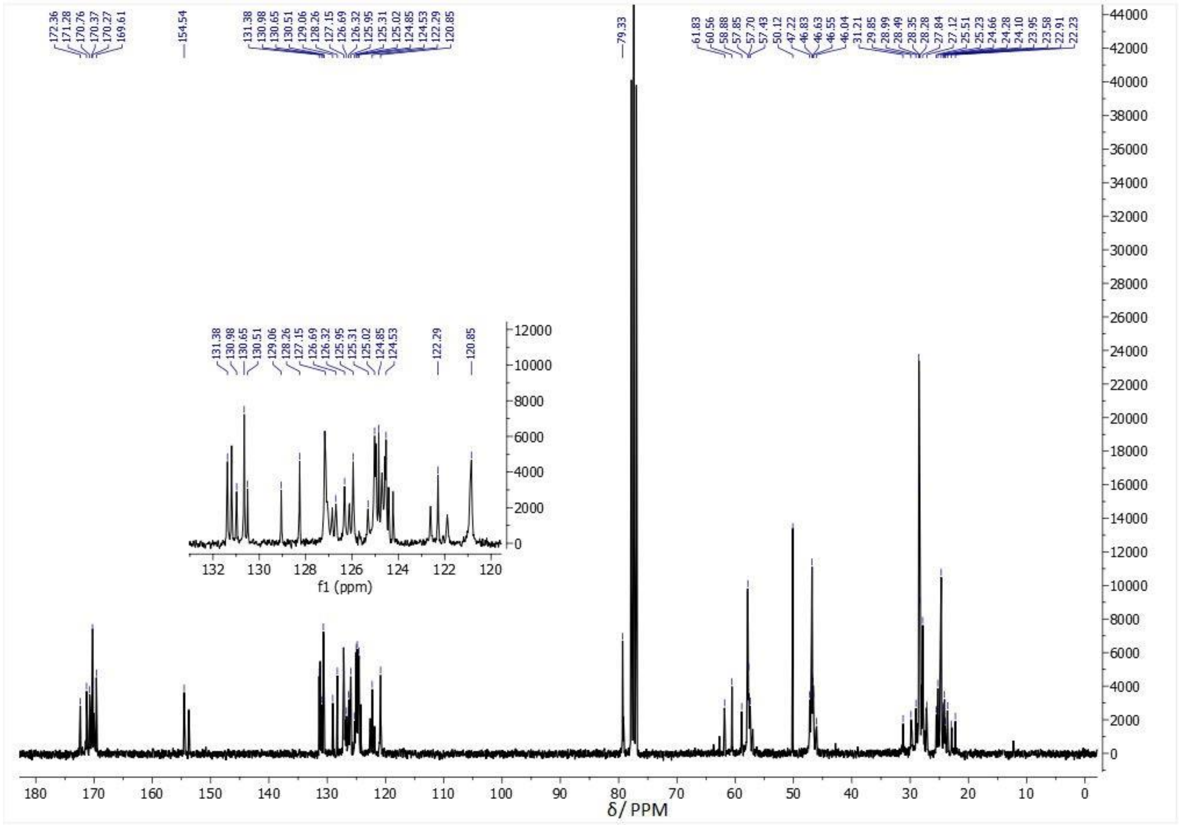

Figure S16. ^{13}C NMR (75 MHz, $CDCl_3$) spectrum recorded for compound **PY-P$_6$.**

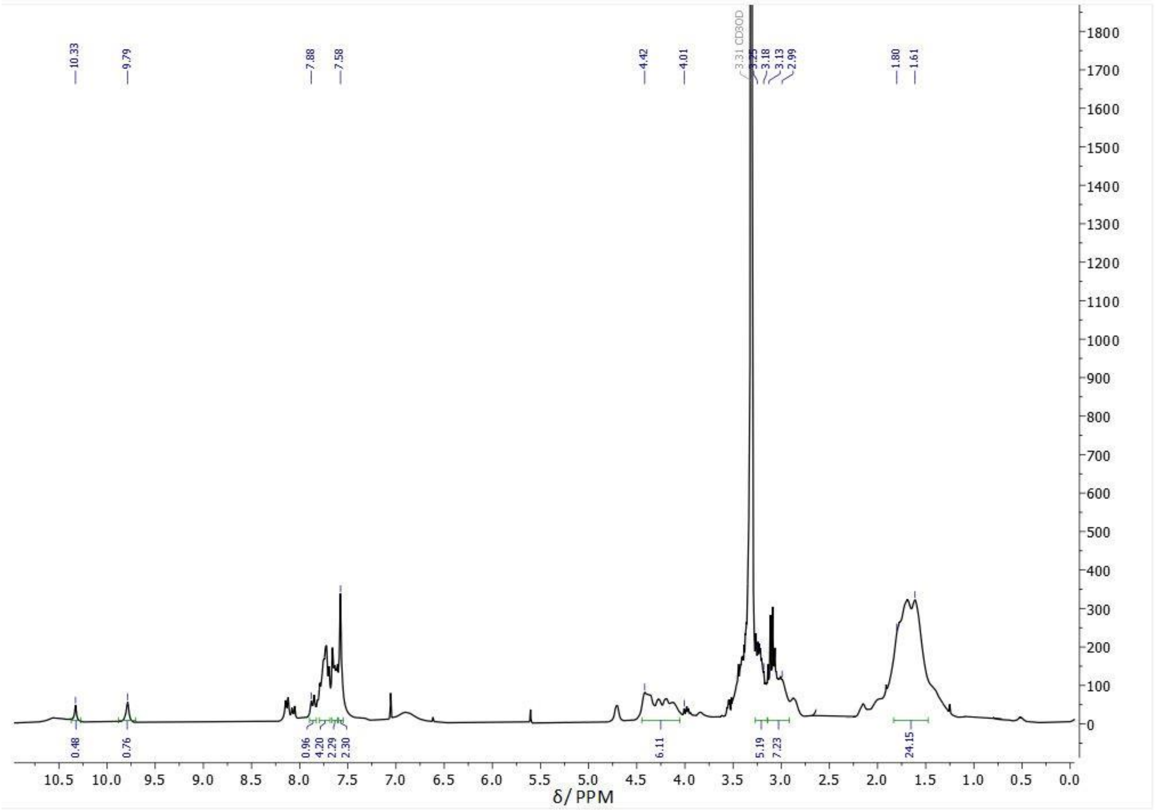

Figure S17. ^{1}H NMR (500 MHz, MeOD) spectrum recorded for compound **PY-P$_6$-NH**.

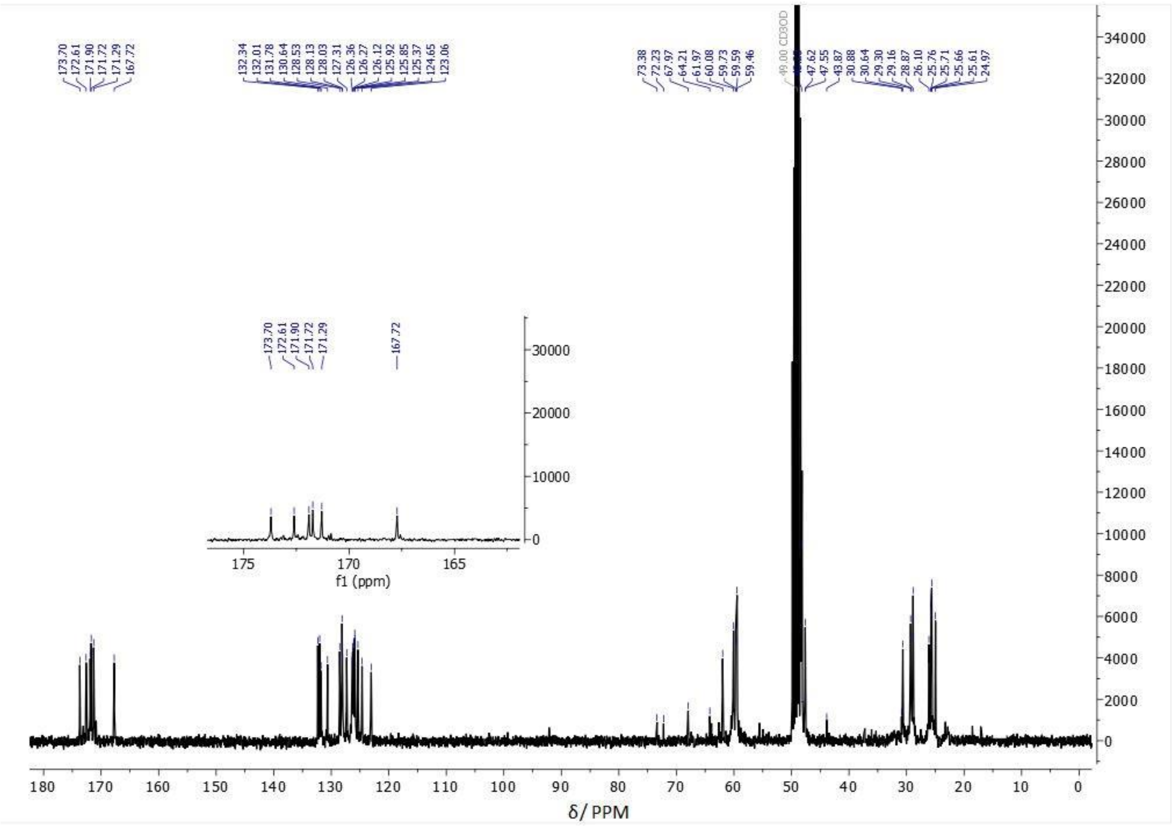

Figure S18. ^{13}C NMR (75 MHz, MeOD) spectrum recorded for compound **PY-P$_6$-NH**.

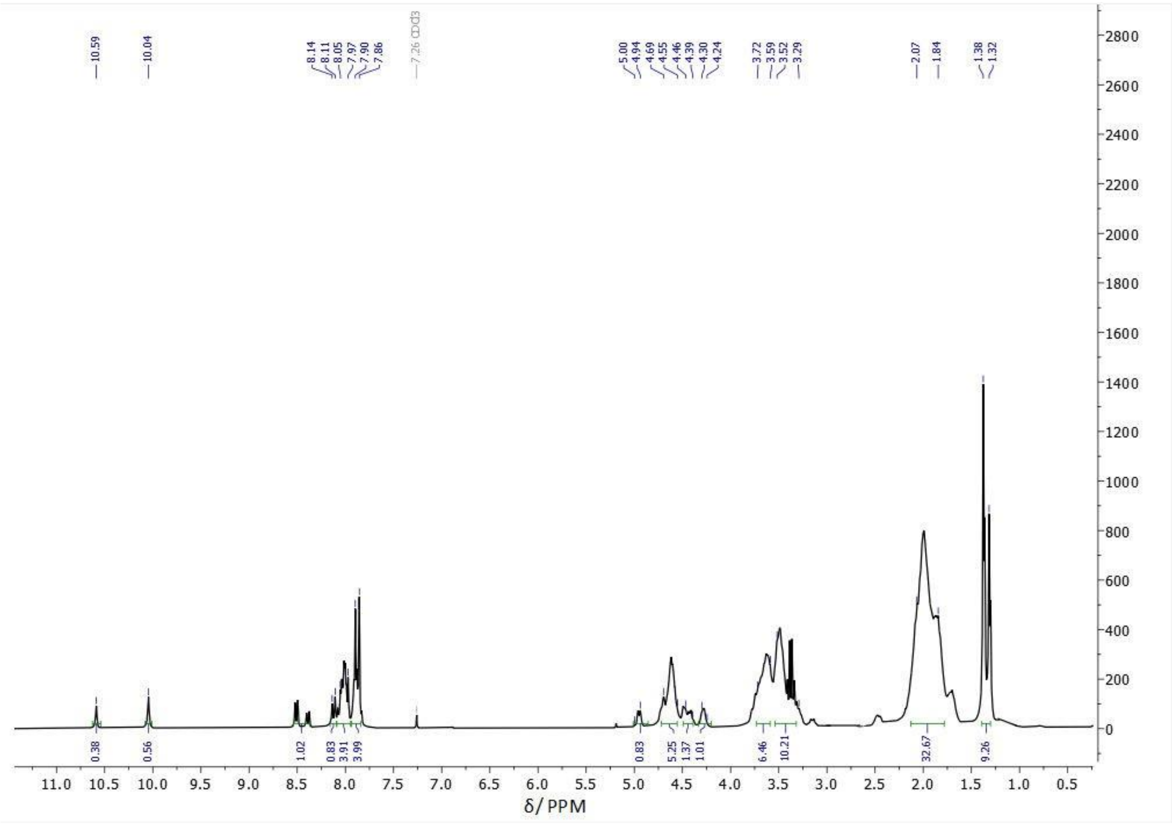

Figure S19. ^{1}H NMR (300 MHz, $CDCl_3$) spectrum recorded for compound **PY-P$_8$**.

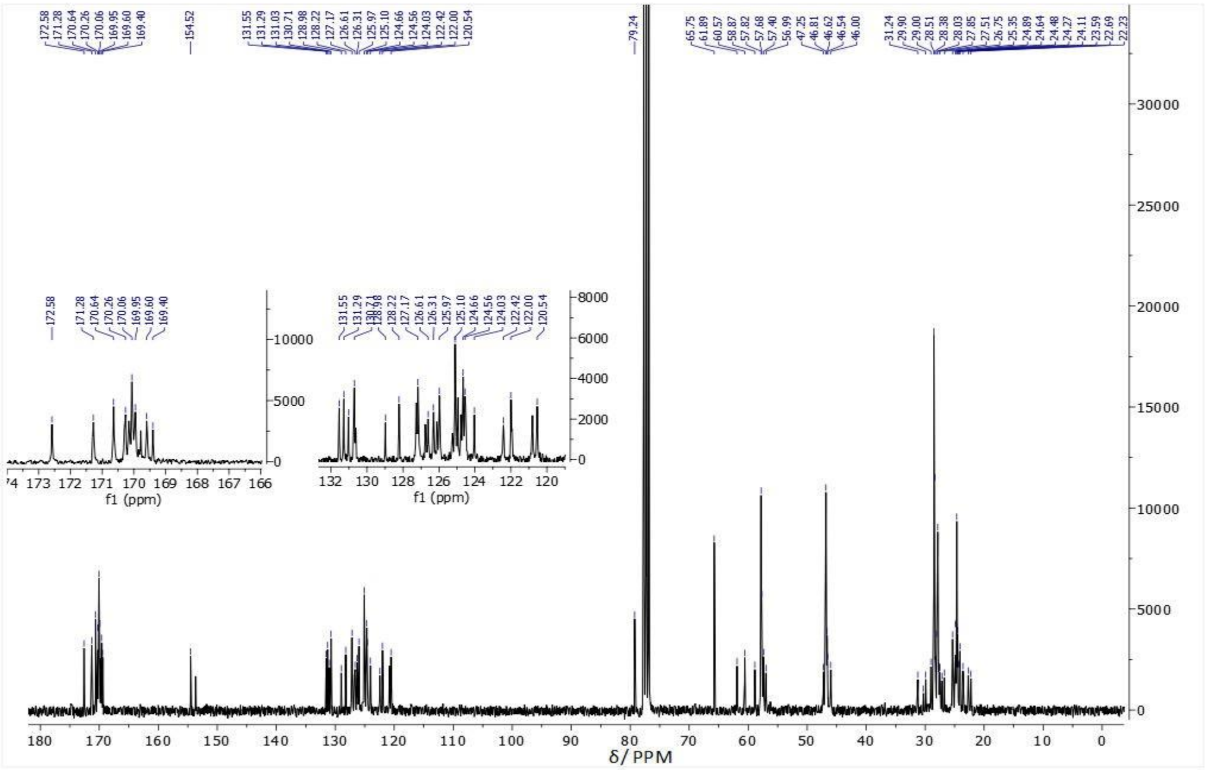

Figure S20. ^{13}C NMR (75 MHz, $CDCl_3$) spectrum recorded for compound **PY-P$_8$**.

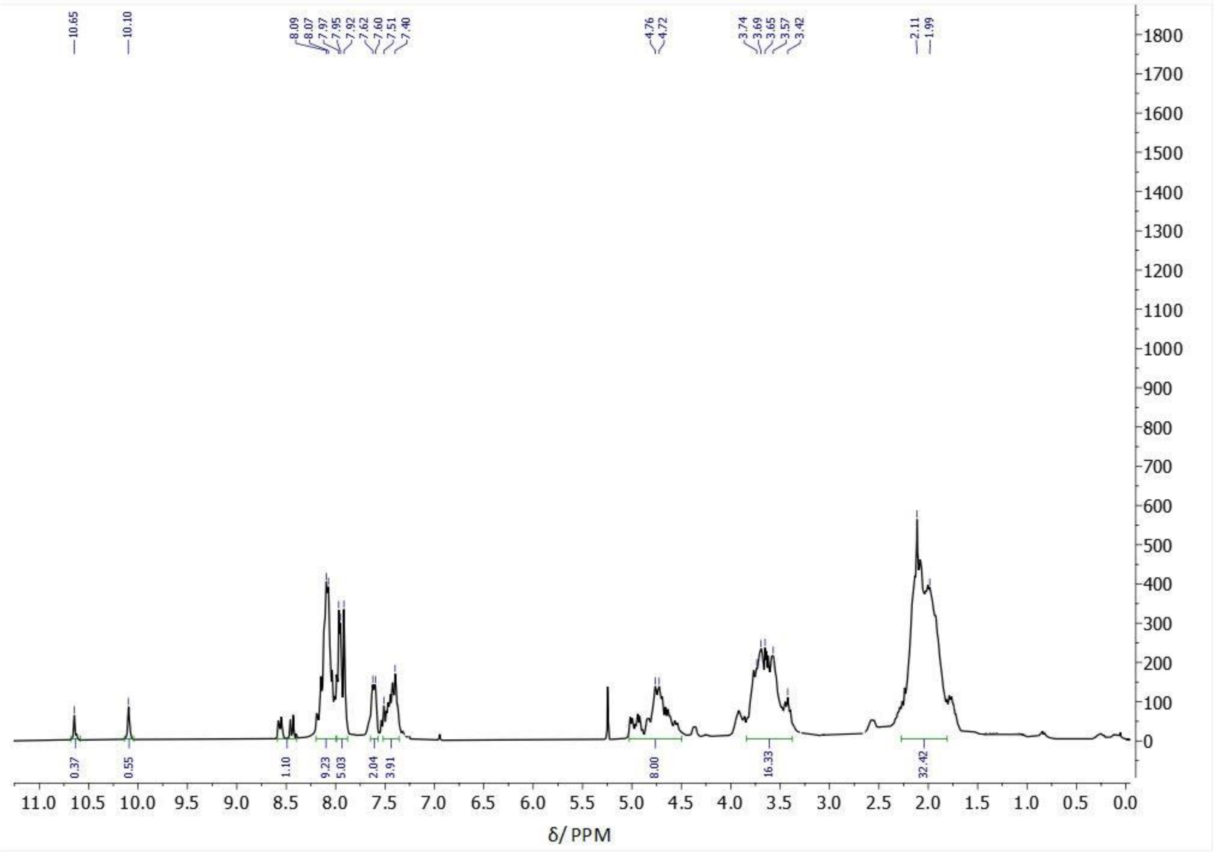

Figure S21. ^{1}H NMR (700 MHz, $CDCl_3$) spectrum recorded for compound **PY-P$_8$-PER**.

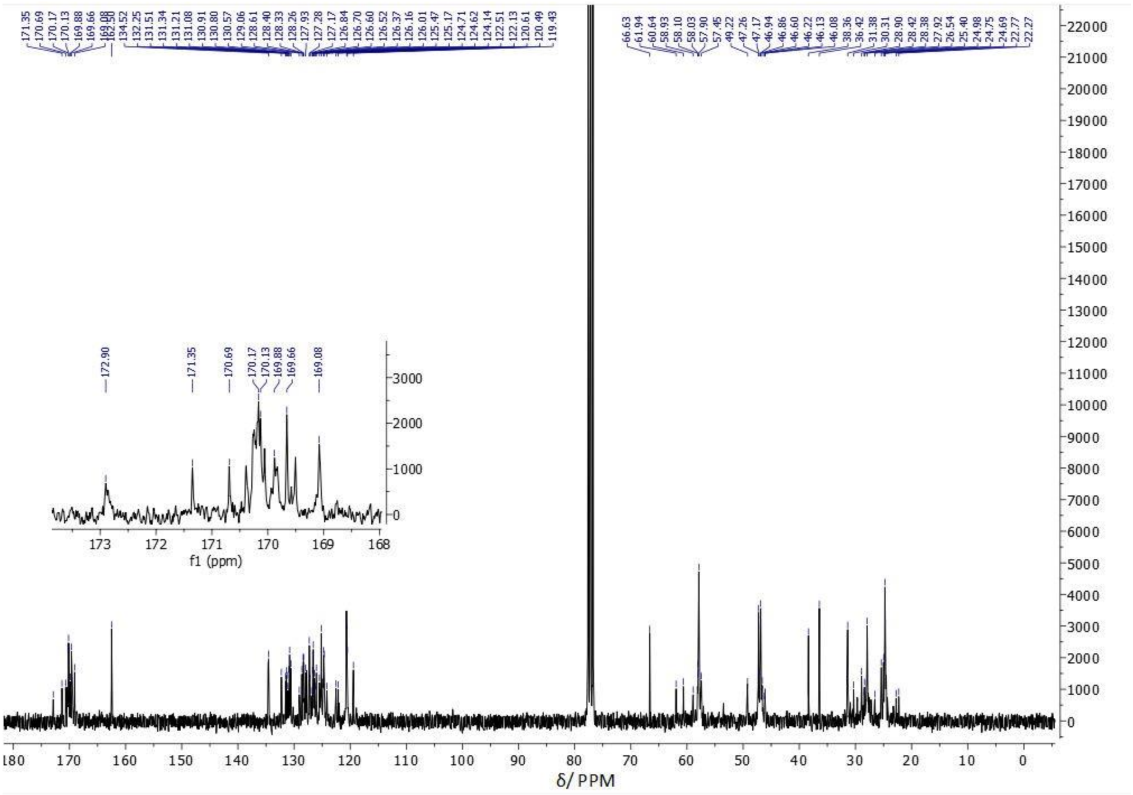

Figure S22. ^{13}C NMR (75 MHz, $CDCl_3$) spectrum recorded for compound **PY-P$_8$-PER**.

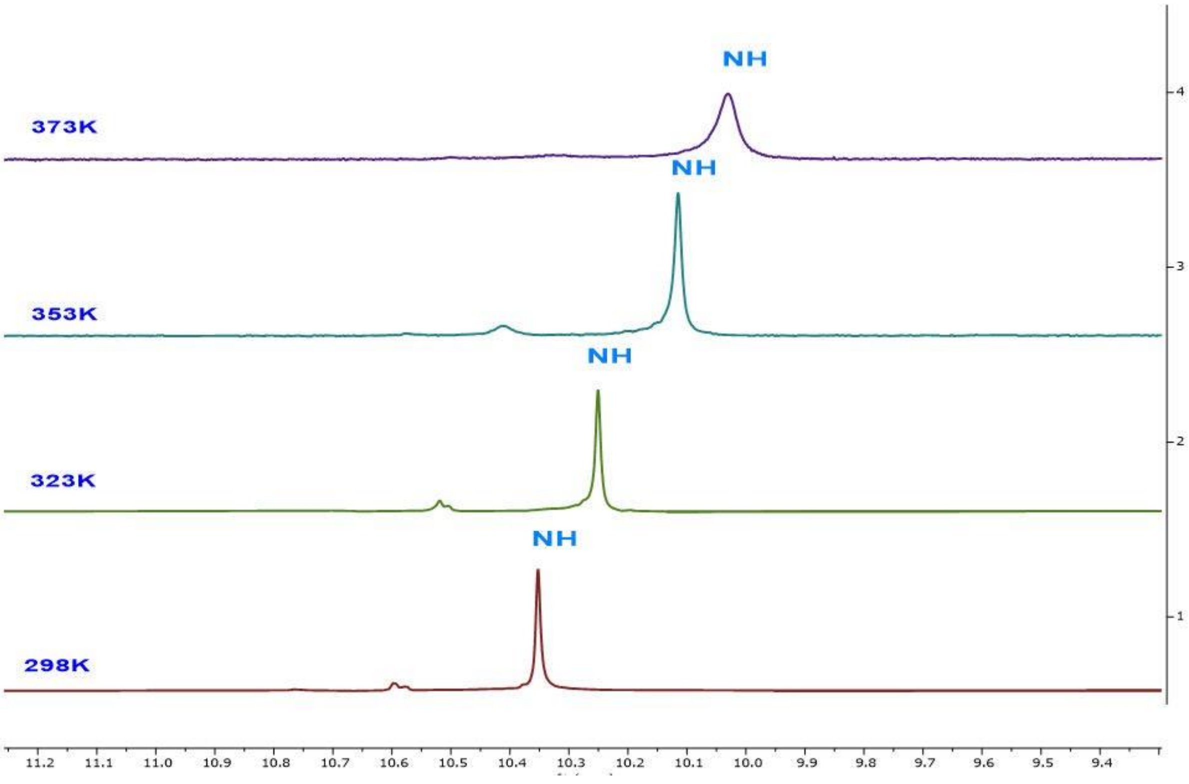

Figure S23. Variable temperature ^1H NMR spectra of **PY-P$_8$-PER** in D_6-DMSO showing the NH peaks.

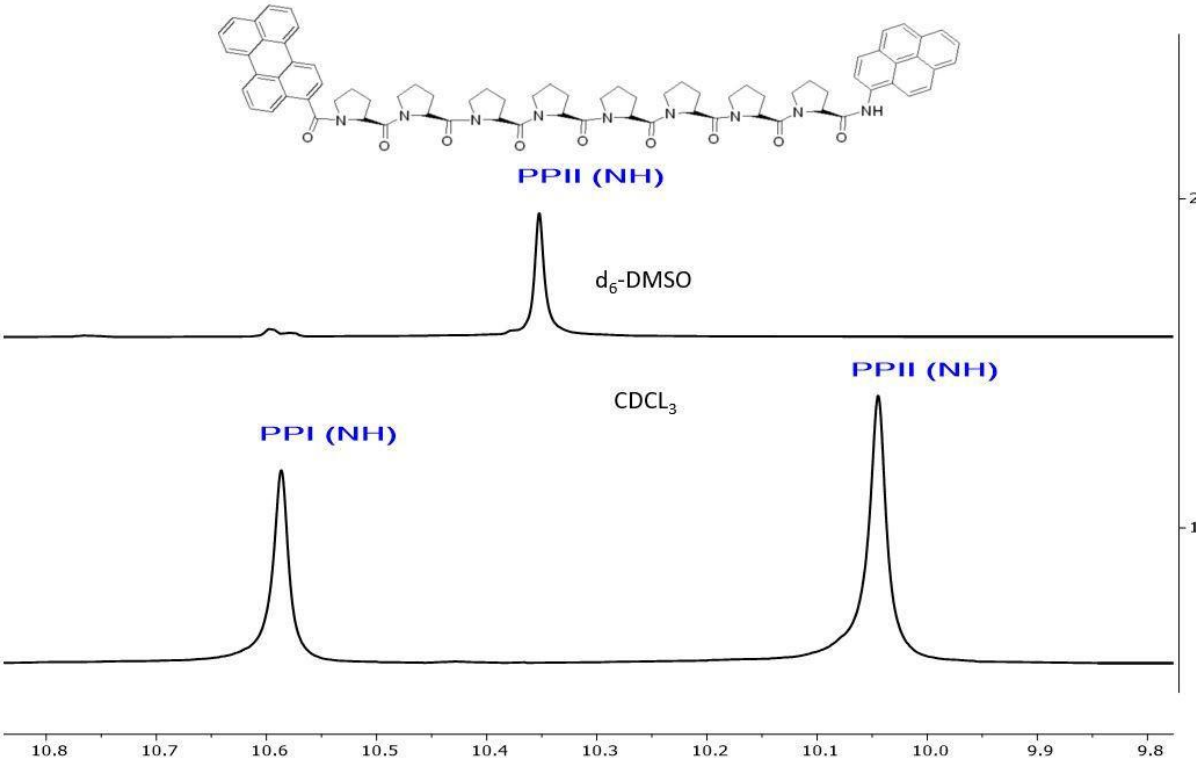

Figure S24. ^{1}H NMR (700 MHz) spectrum recorded for compound **PY-P$_8$-PER** in D$_6$-DMSO (above) and in CDCl$_3$ (below).

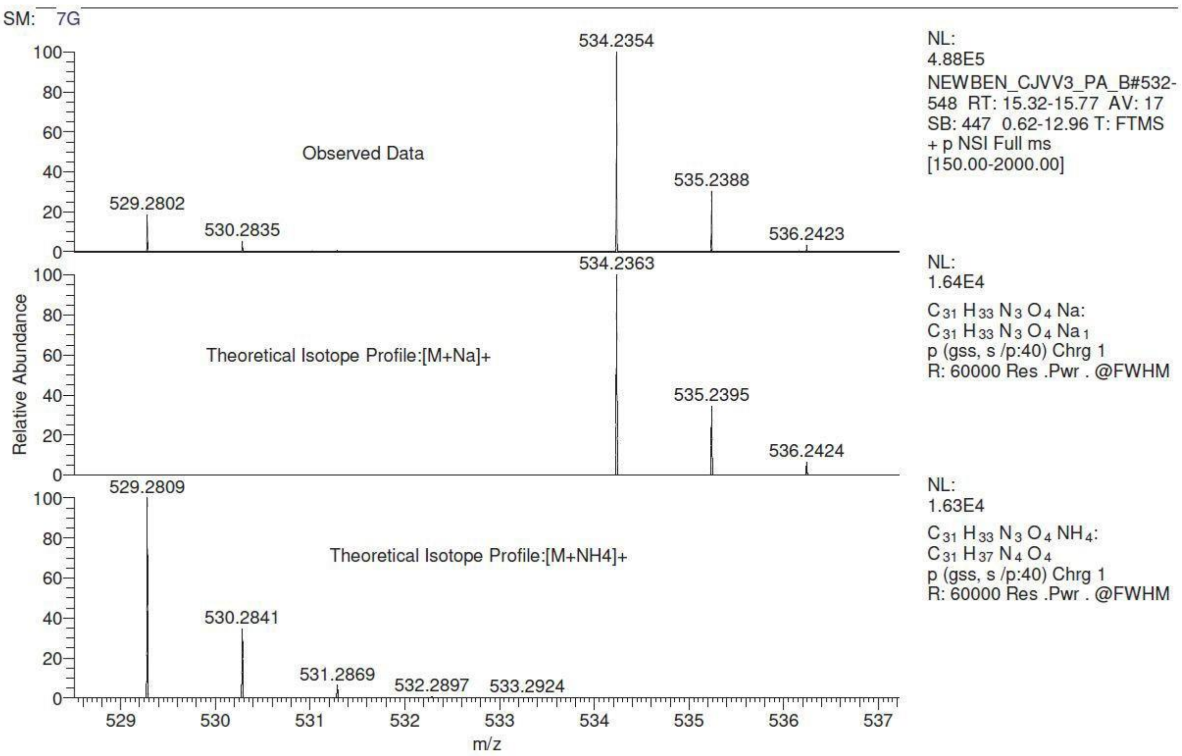

Figure S25. Observed and theoretical mass spectra for **PY-P$_2$** showing its $[M+Na]^+$ and $[M+NH_4]^+$ ions.

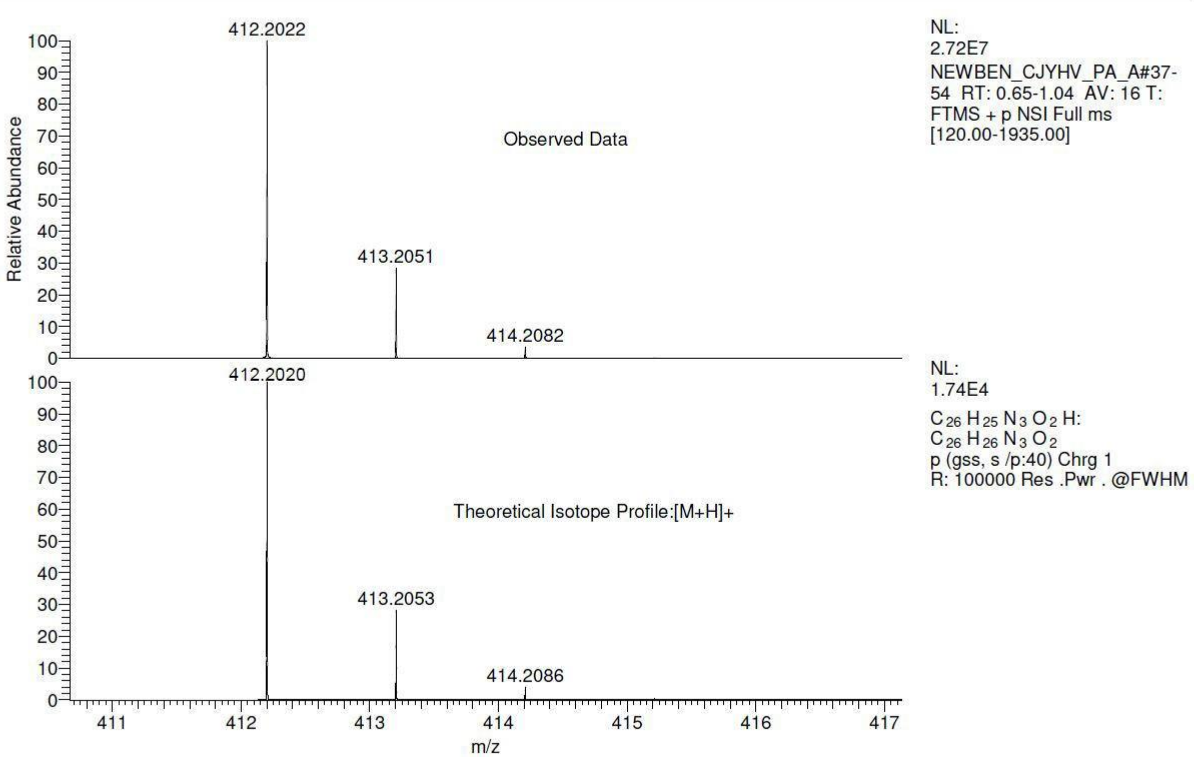

Figure S26. Observed and theoretical mass spectra for **PY-P$_2$-NH** showing its $[M+H]^+$ ions.

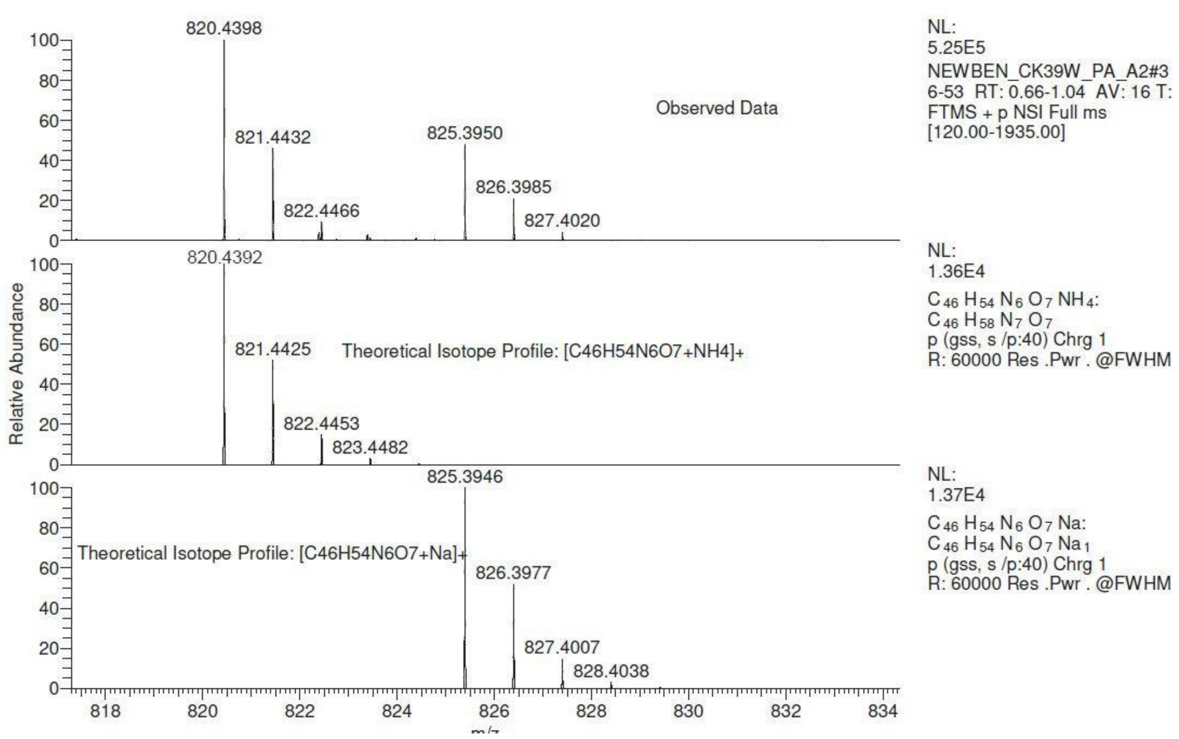

Figure S27. Observed and theoretical mass spectra for **PY-P$_4$** showing its $[M+Na]^+$ and $[M+NH_4]^+$ ions.

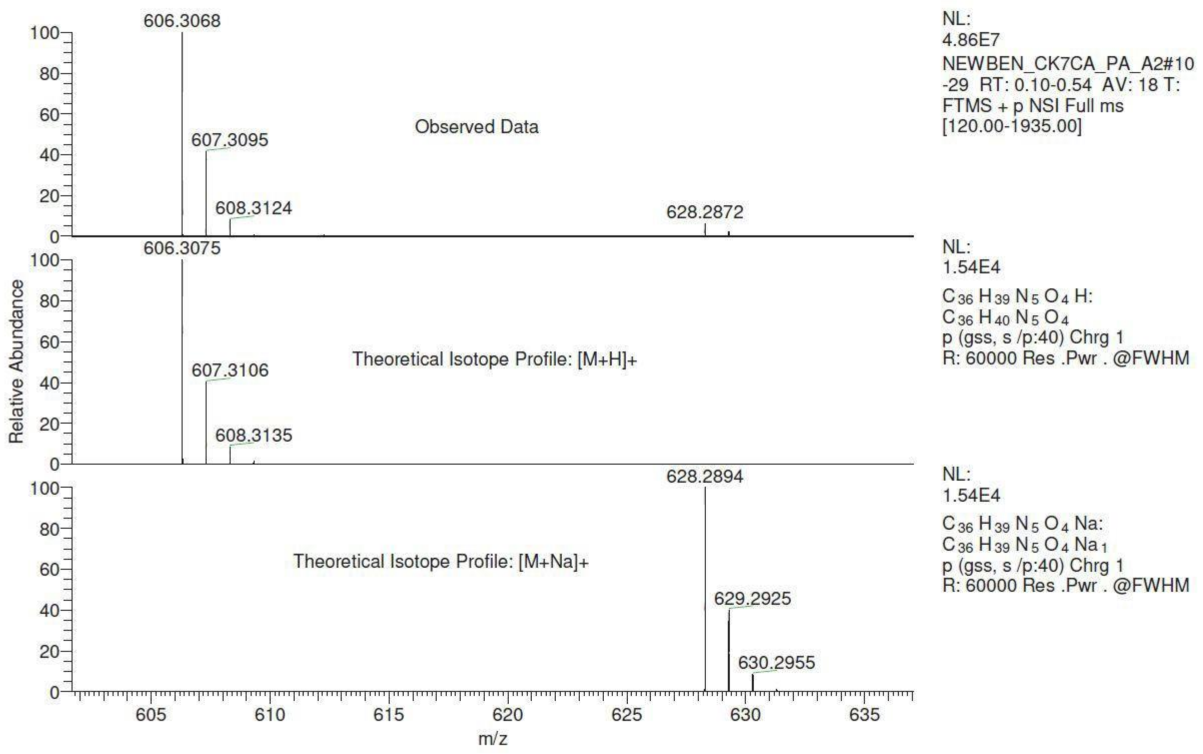

Figure S28. Observed and theoretical mass spectra for **PY-P$_4$-NH** showing its $[M+H]^+$ and $[M+Na]^+$ions.

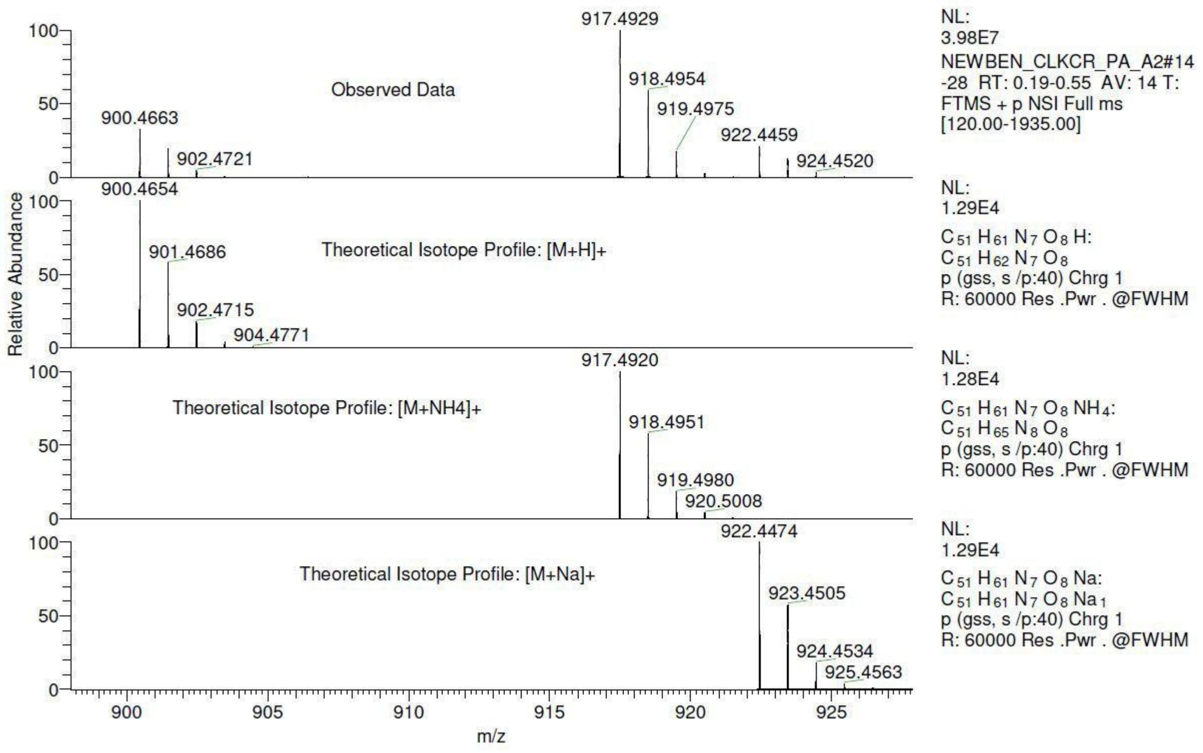

Figure S29. Observed and theoretical mass spectra for **PY-P$_6$** showing its $[M+H]^+$, $[M+NH_4]^+$ and $[M+Na]^+$ ions.

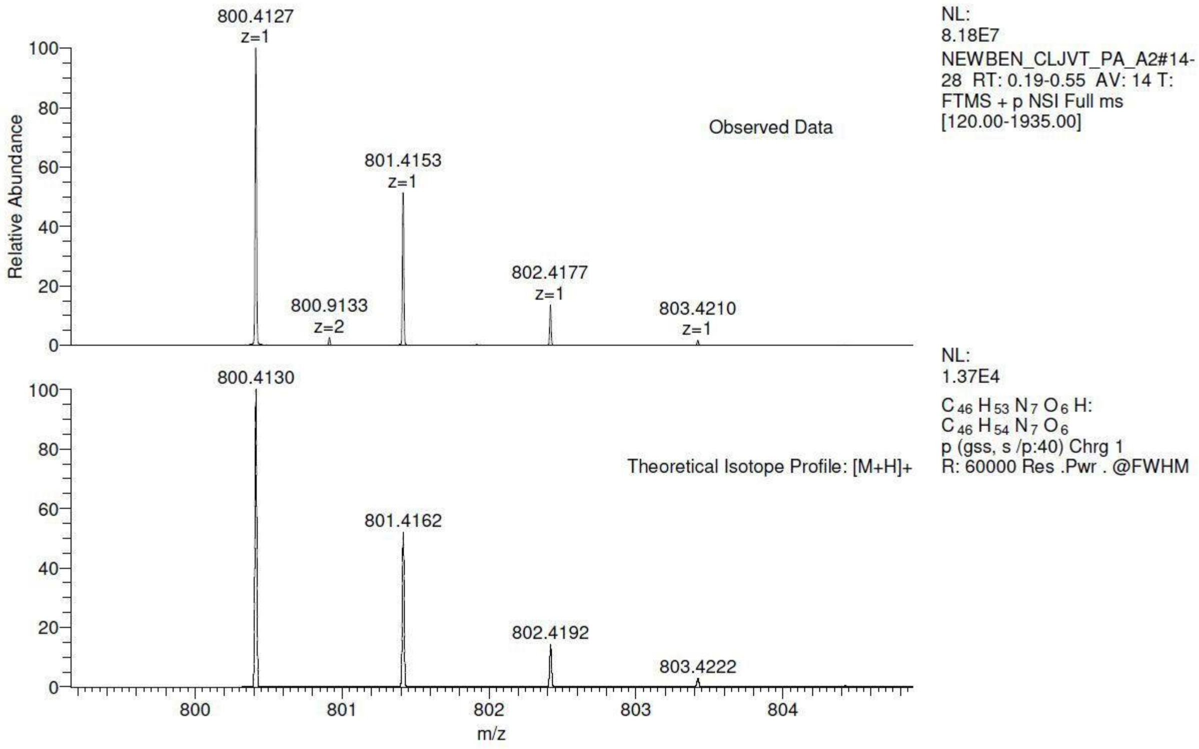

Figure S30. Observed and theoretical mass spectra for **PY-P$_6$-NH** showing its $[M+H]^+$ions.

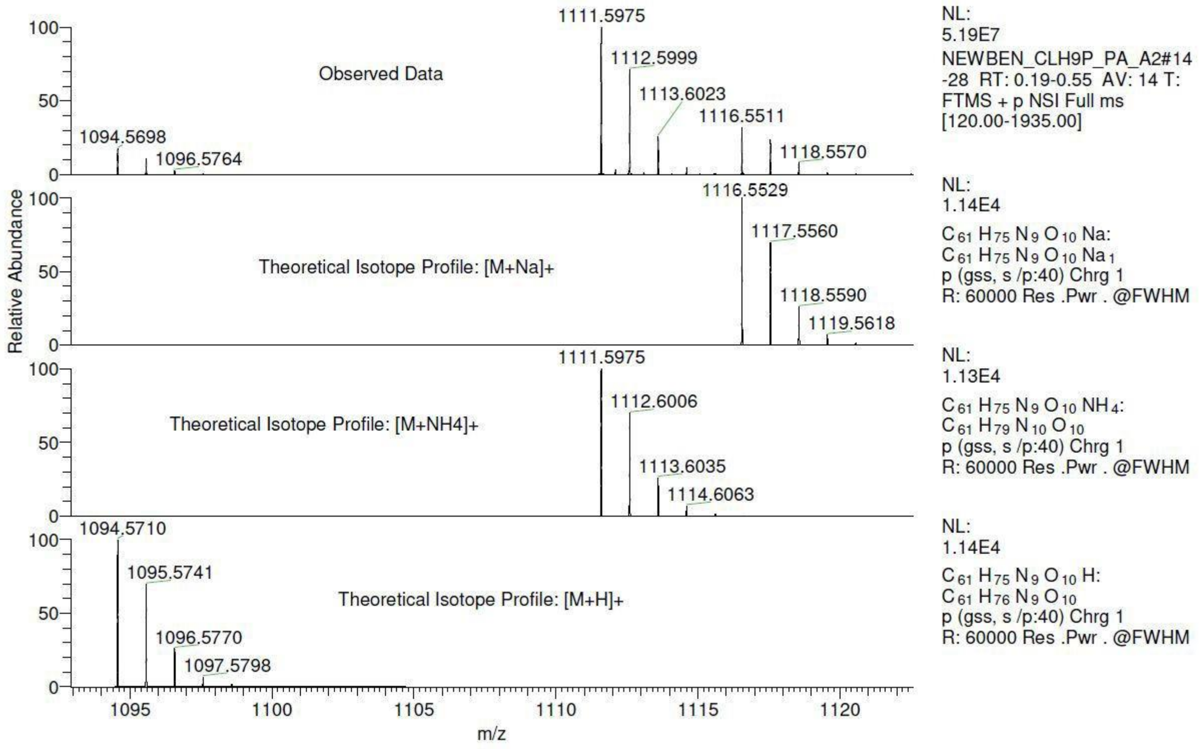

Figure S31. Observed and theoretical mass spectra for **PY-P$_8$** showing its $[M+H]^+$, $[M+Na]^+$ and $[M+NH_4]^+$ions.

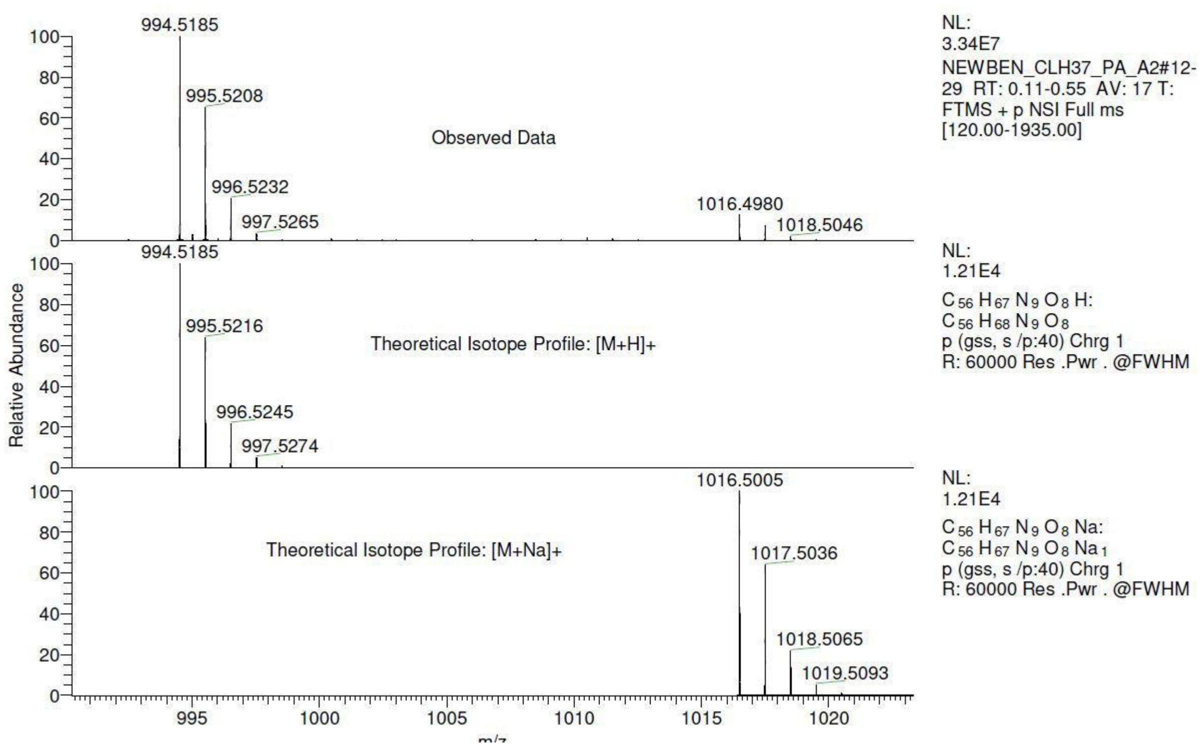

Figure S32. Observed and theoretical mass spectra for **PY-P$_8$-NH** showing its $[M+H]^+$ and $[M+Na]^+$ ions.

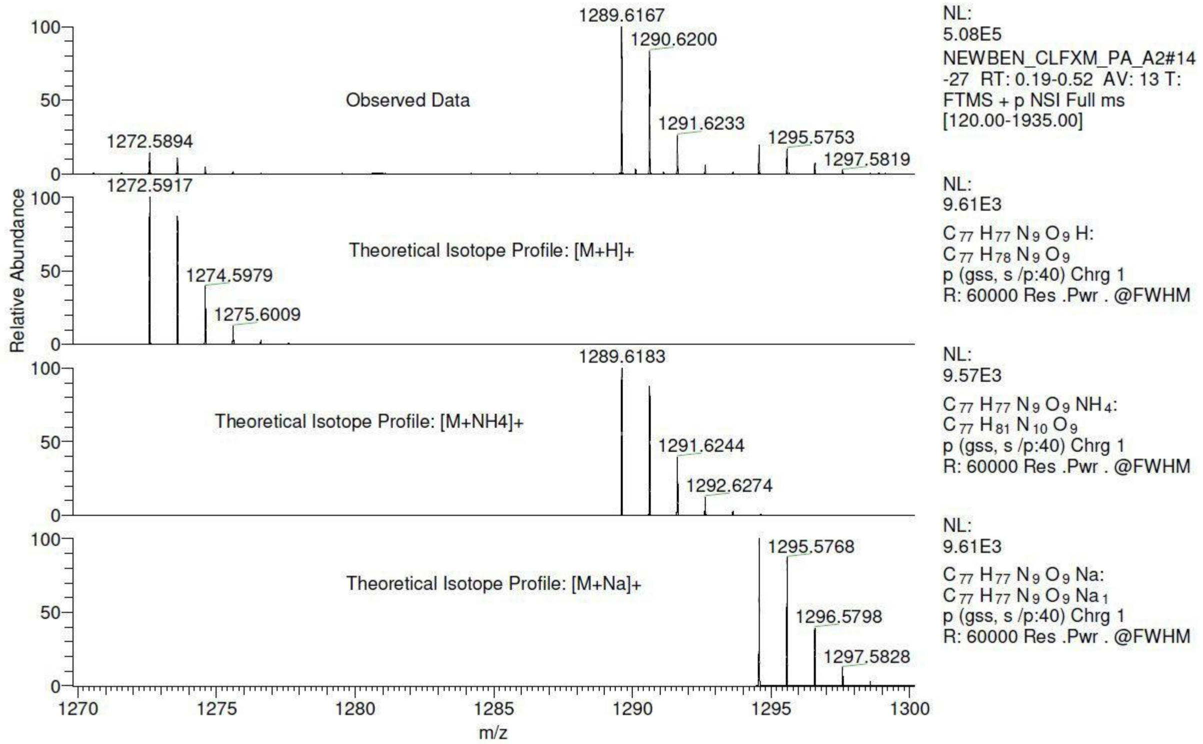

Figure S33. Observed and theoretical mass spectra for **PY-P$_8$-PER** showing its $[M+H]^+$, $[M+Na]^+$and $[M+NH_4]^+$ ions.

S2. Crystal structure determination of PY-P_2 **CCDC 2283731**

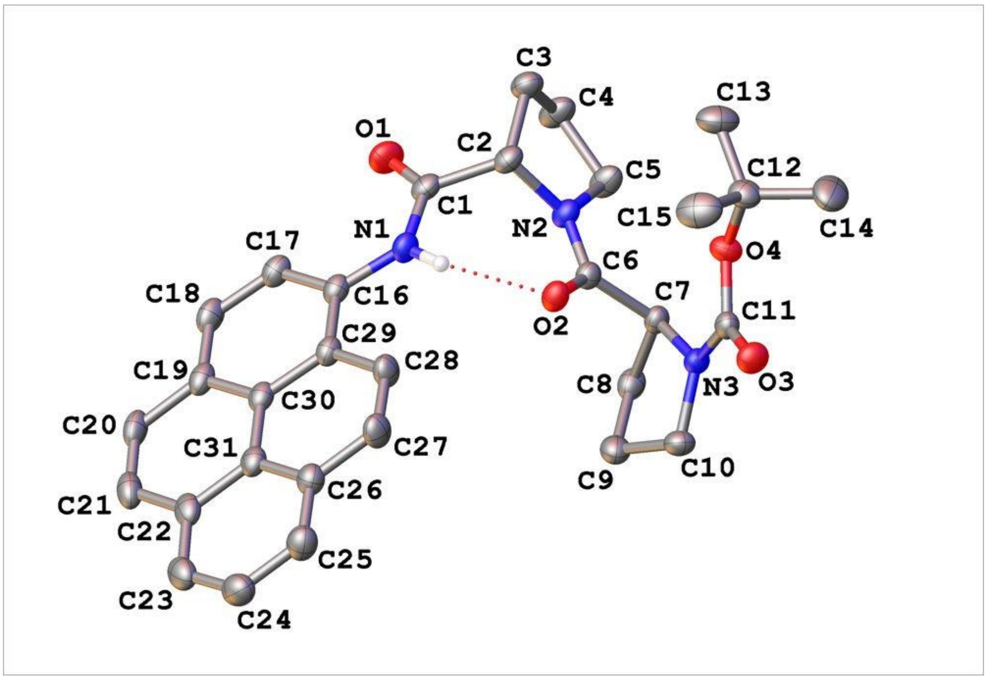

Table S1. Crystal data and structure refinement for PY-P_2.

Identification code	**CCDC 2283731**
Empirical formula	$C_{31}H_{33}N_3O_4$
Formula weight	511.60
Temperature/K	150.0(2)
Crystal system	monoclinic
Space group	$P2_1$
a/Å	10.3503(2)
b/Å	8.7745(2)
c/Å	14.9164(3)
α/°	90
β/°	104.498(2)
γ/°	90
Volume/$Å^3$	1311.55(5)
Z	2
$\rho_{calc}g/cm^3$	1.295
μ/mm^{-1}	0.693
F(000)	544.0
Crystal size/mm^3	0.14 × 0.07 × 0.01
Radiation	Cu Kα (λ = 1.54184)
2Θ range for data collection/°	6.12 to 155.638
Index ranges	-13 ≤ h ≤ 12, -10 ≤ k ≤ 10, -18 ≤ l ≤ 18
Reflections collected	15516
Independent reflections	5086 [R_{int} = 0.0310, R_{sigma} = 0.0326]

Data/restraints/parameters	5086/1/350
Goodness-of-fit on F^2	1.067
Final R indexes [I>=2σ (I)]	R_1 = 0.0313, wR_2 = 0.0775
Final R indexes [all data]	R_1 = 0.0345, wR_2 = 0.0791
Largest diff. peak/hole / e Å^{-3}	0.17/-0.15
Flack parameter	0.05(9)

Experimental

Crystal structure data for **PY-P₂** were collected on a XtaLAB Synergy single source HyPix-Arc 100 diffractometer equipped with a fine-focus sealed X-ray tube ($\lambda_{Cu\ K\alpha}$ = 1.54184 Å) and an Oxford Cryosystems CryostreamPlus open-flow N_2 cooling device. Cell refinement, data collection and data reduction were undertaken via CrysAlisPro 1.171.42.73a (Rigaku OD, 2022). Intensities were corrected for absorption using CrysAlisPro 1.171.42.73a (Rigaku Oxford Diffraction, 2022). Analytical numeric absorption correction used a multifaceted crystal model based on expressions derived by Clark and Reid.[S1] Empirical absorption correction was made using spherical harmonics as implemented in the SCALE3 ABSPACK scaling algorithm. The crystal structure was solved with Olex2,[S2] using SHELXT 2014/5[S3] and refined with XL.[S4]

Crystal structure determination of PY-P_2

Crystal Data for $C_{31}H_{33}N_3O_4$ (*M* =511.60 g/mol): monoclinic, space group $P2_1$ (no. 4), *a* = 10.3503(2) Å, *b* = 8.7745(2) Å, *c* = 14.9164(3) Å, *β* = 104.498(2)°, *V* = 1311.55(5) Å^3, *Z* = 2, *T* = 150.0(2) K, μ(Cu Kα) = 0.693 mm^{-1}, *Dcalc* = 1.295 g/cm^3, 15516 reflections measured (6.12° ≤ 2Θ ≤ 155.638°), 5086 unique (R_{int} = 0.0310, R_{sigma} = 0.0326) which were used in all calculations. The final R_1 was 0.0313 (I > 2σ(I)) and wR_2 was 0.0791 (all data).

CCDC 2283731

S3. Further spectroscopic information

Absorption spectra were recorded with an Hitachi U3310 spectrophotometer using a variety of quartz cuvettes of disparate path length. Additional measurements were made with a variable path length cell to ensure the absence of optical saturation. Molar absorption coefficients were determined from three independent samples after ensuring the validity of the Beer-Lambert law. At the high concentrations required in the search for excimer emission,[S5] **PY-P$_2$** showed extreme self-absorption.[S6] Fluorescence spectra were recorded with an Hitachi F-4500 spectrophotometer and were fully corrected for any instrumental artefacts. Glass cut-off filters were used to exclude scattered excitation light and/or Raman bands associated with the solvent. Excitation spectra were recorded with the same instrument and were corrected using a calibration curve generated according to Parker and Rees.[S7] Emission and excitation spectra were averaged multiple times before data analysis. In all cases, optically dilute solutions were used; this is especially important for the excitation spectra. Deconstruction of reduced spectra into Gaussian components[S8] was made using PEAKFIT. In all other cases, data files were transferred to a PC for manipulation using MATLAB. All optical measurements were repeated several times. Fluorescence quantum yields were measured in deoxygenated $CHCl_3$ solution using a StellaNet integrating sphere with excitation being provided by a stabilised LED source. The collimated light beam was attenuated with neutral density filters, with part (i.e., 5%) of the beam was directed to a radiometer for continuous measurement of the intensity. The operating protocol introduced by Beeby *et al*[S9] was used to obtain the quantum yields and error limits were established from three separate measurements. For the ratiometric measurements, the spectral region of interest was isolated with a narrow bandpass filter. All data analysis was carried out using MATLAB.

Time-resolved fluorescence studies were made with a PTI EasyLife instrument equipped with high performance pulsed LED sources emitting at 330 nm or 440 nm. Excitation was made at 90° to detection. The instrument response function (IRF) was generated using Ludox scattering media. Glass cut-off filters, together with narrow bandpass filters, were used to prevent scattered excitation light reaching the detector. Dilute solutions were used in all cases with the sample being sealed in the cell after pruging with dried N_2. After deconvolution of the IRF, the best temporal resolution of this set-up was in the region of 200 ps. Data analysis was made using in-house software based on the Marquardt algorithm with both the pre-

exponentials and lifetimes treated as variable parameters. The decay records were collected at different counts-per-peak channel (cpc) and analysis was made from before the peak channel to the end of the decay record. Initial analyses were based on the sum of exponentials with the reduced chi-squared parameter (χ^2) being used as the primary screening indicator (Equation S1); Δn refers to the total number of channels used for analysis and p is the number of parameters used in the fit. The weighted reduced residuals (Rt,i) were calculated from Equation S2 where D(model) and D(expt) refer to the intensities at time t for the model fit and for the experimental curve.

$$\chi^2 = \sum_i \frac{\left[D(model) - D(expt)\right]^2}{\sigma^2} \qquad (S1a)$$

$$\sigma^2 = (\Delta n - p + 1)D(expt) \qquad (S1b)$$

$$R(t,i) = \frac{D(model,t) - D(expt,t)}{\sqrt{D(expt,t)}} \qquad (S2)$$

Time-resolved transient absorption measurements were obtained with an Applied Photophysics LKS-70 system using a 4 ns pulsed Quantel Brilliant B Nd:YAG laser with the 1067 nm output being frequency doubled to 534 nm. The repetition rate of the laser was 10 Hz with the power varying from 1 to 45 mJ per pulse. Appropriate excitation wavelengths were selected with an OPO pumped by the third or fourth harmonic of the Q-switched Nd:YAG laser. As necessary, samples were saturated with nitrogen for 20 min before starting the experiment. For kinetic measurements made at a fixed wavelength, 200 individual laser shots were averaged for each sample and solvent under the same experimental conditions, and three different laser intensities were used. Transient differential absorption spectra were compiled point-by-point with 5 individual records being averaged at each wavelength. Metal screen filters were employed to obtain the different laser intensities. The signals were detected with a PMT having a five stage dynode chain for high current linearity, amplified by a preamplifier, recorded by a digital oscilloscope (Tektronix DPO7254C), and subsequently transferred to a PC for data analysis and storage. A plane ruled grating, 1800 g/mm, with 500 nm blaze, was used as standard. The average spectral dispersion was 1.8 nm/mm. On a single shot basis, the sensitivity of the absorption change at 500 nm was ca. 0.0005. Analysis of the background corrected signals was made using in-house software.[S10] Bimolecular triplet-triplet

annihilation effects were treated according to the protocol introduced by Grellmann and Scholtz.[S11]

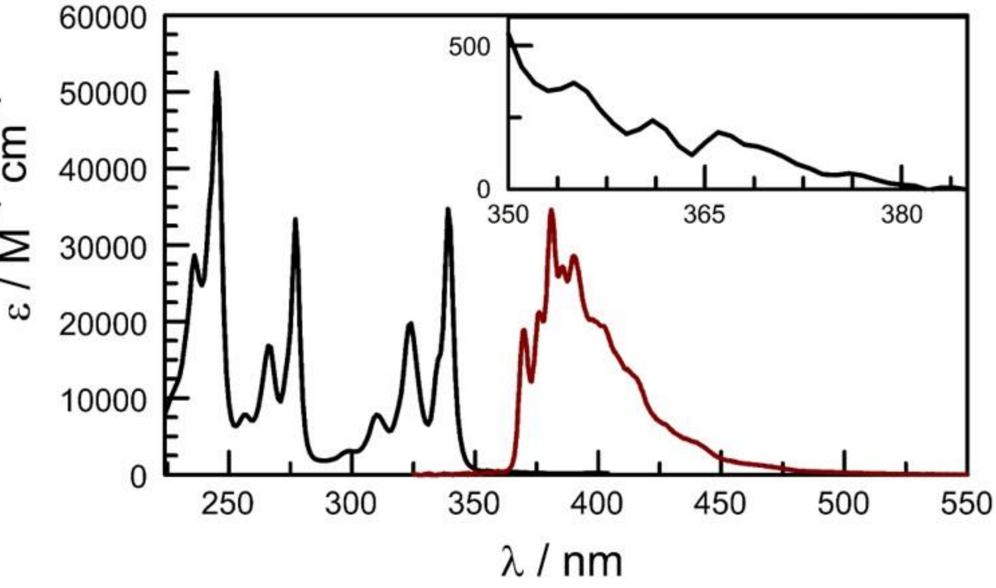

Figure S34. Absorption (black curve) and fluorescence (red curve) spectra recorded for pyrene in cyclohexane solution. The inset shows an expansion of the absorption profile for the $^{1}L_{b}$ state.

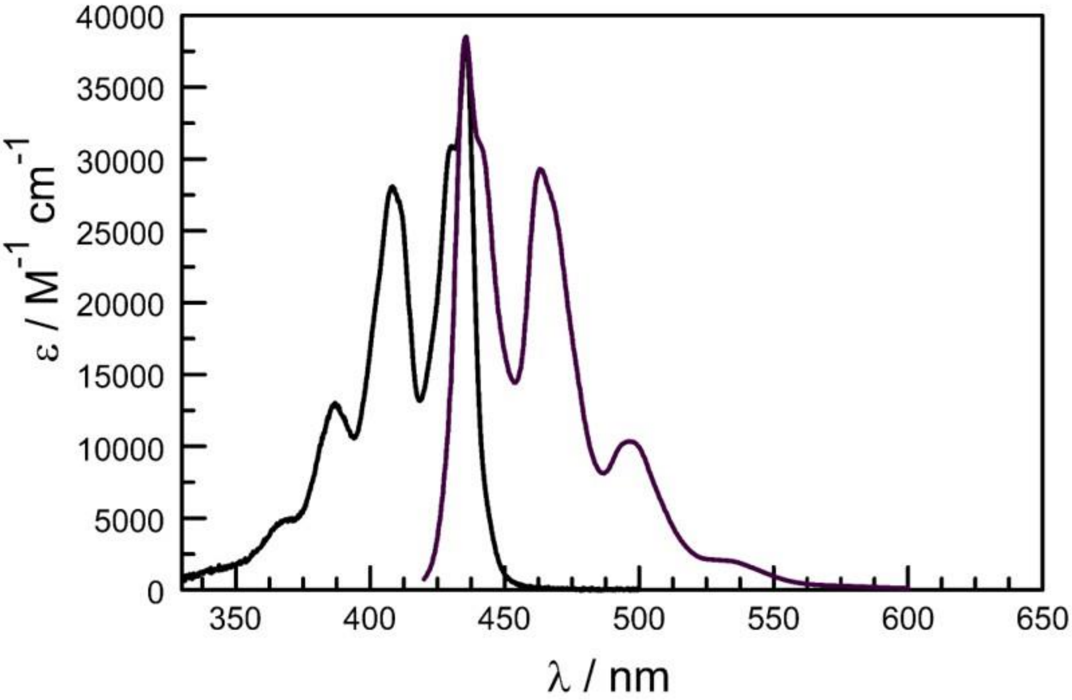

Figure S35. Absorption and fluorescence spectra recorded for perylene in cyclohexane.

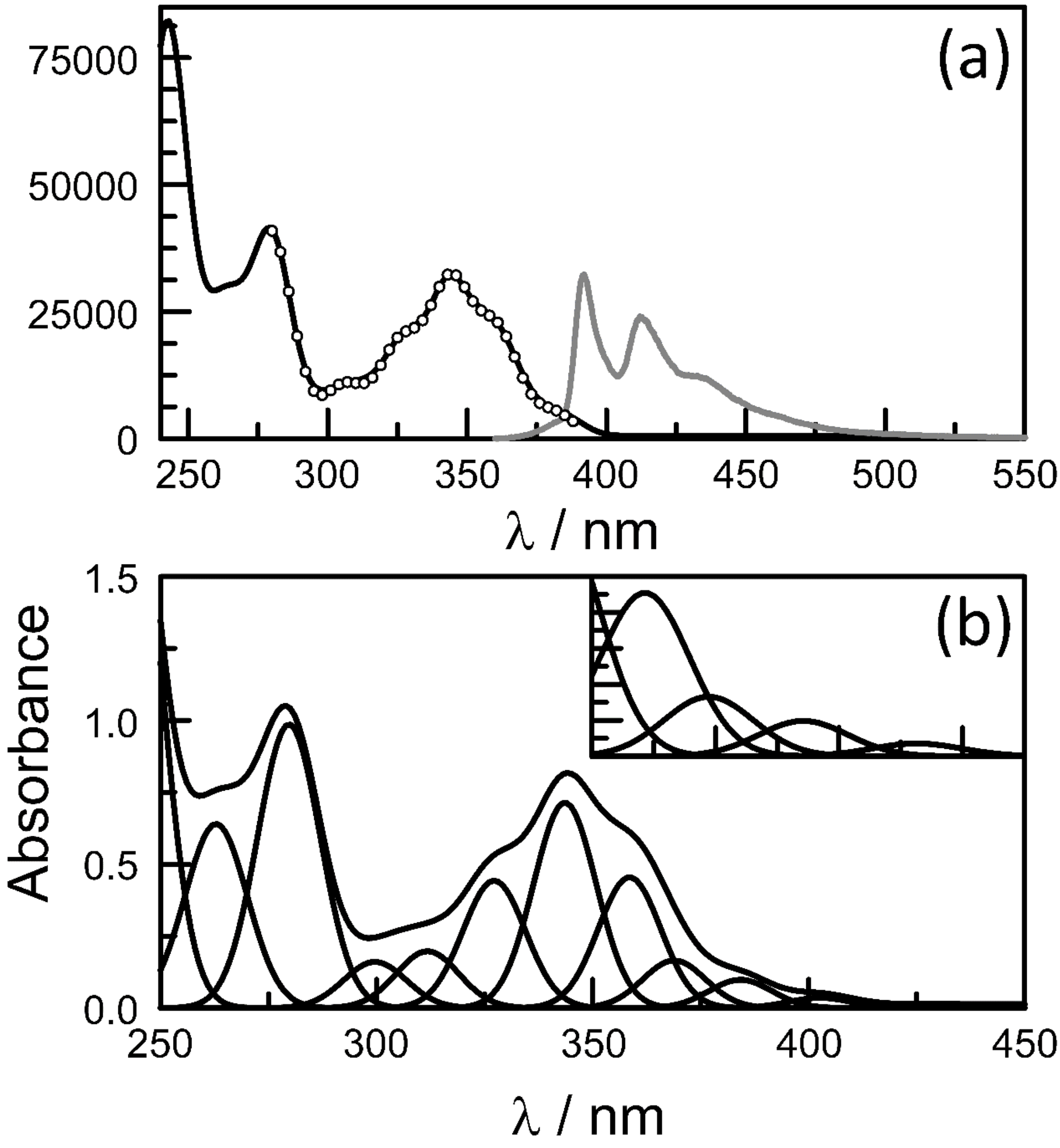

Figure S36. (a) Absorption and emission spectra recorded for **PY-P$_2$** in $CHCl_3$ solution. (b) Gaussian deconstruction of the absorption envelope with expansion of the ^1L_b region.

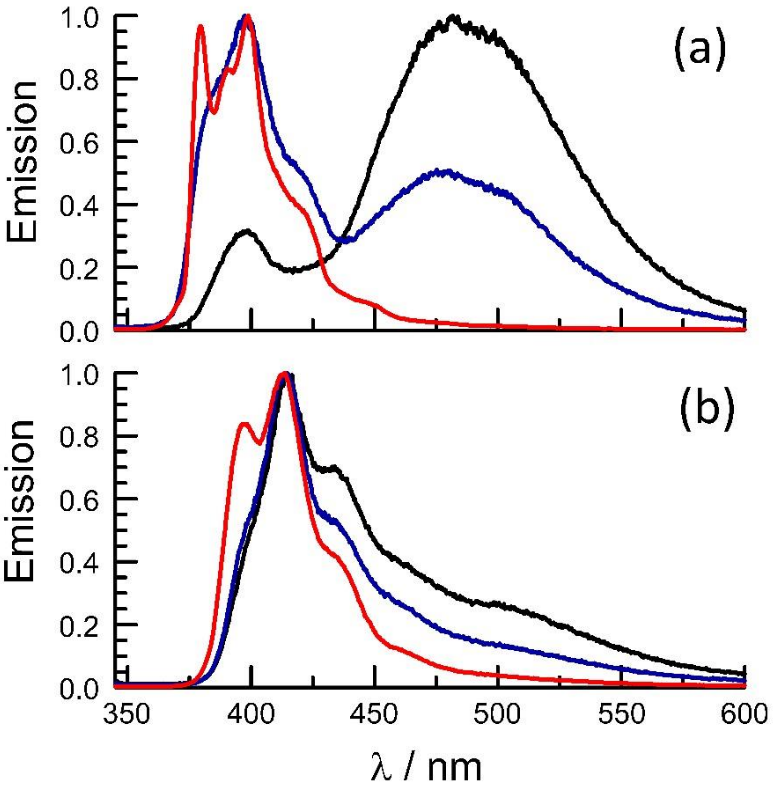

Figure S37. (a) Emission spectra recorded for pyrene in cyclohexane at low (10 μM, red curve) and high (blue and black curves) to illustrate fluorescence from the excimer. The excitation wavelength was 310 nm. (b) The corresponding spectra recorded for **PY-P₂** in cyclohexane. The spectral perturbations are due to self-absorption not excimer formation.

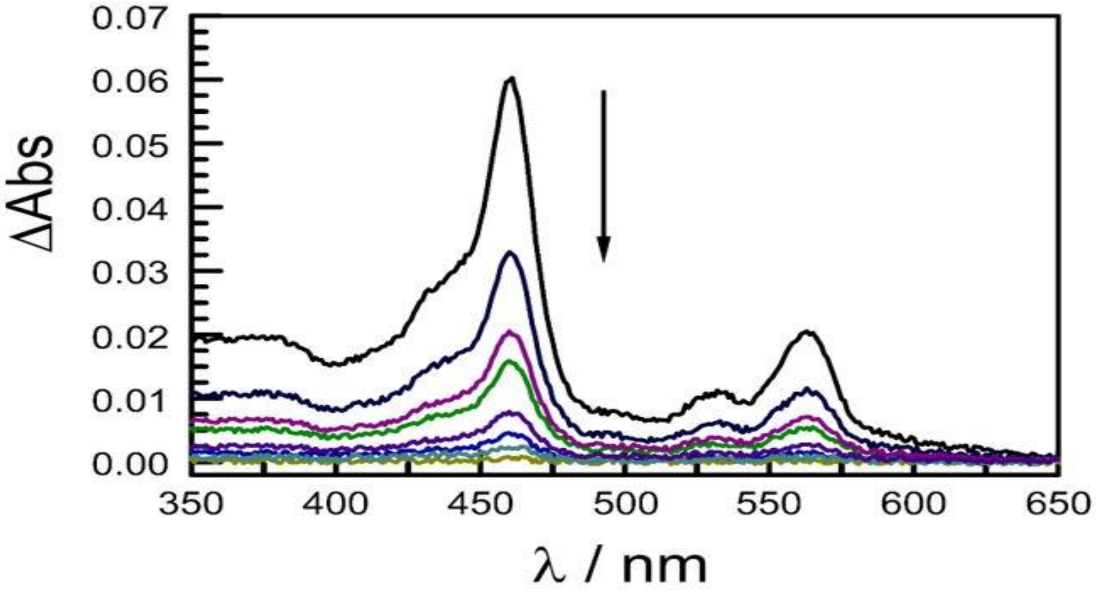

Figure S38. Transient differential absorption spectra recorded for **PY-P$_2$** in deoxygenated $CHCl_3$ following excitation with a 4-ns laser pulse at 340 nm. Spectra were recorded at different times after the excitation pulse.

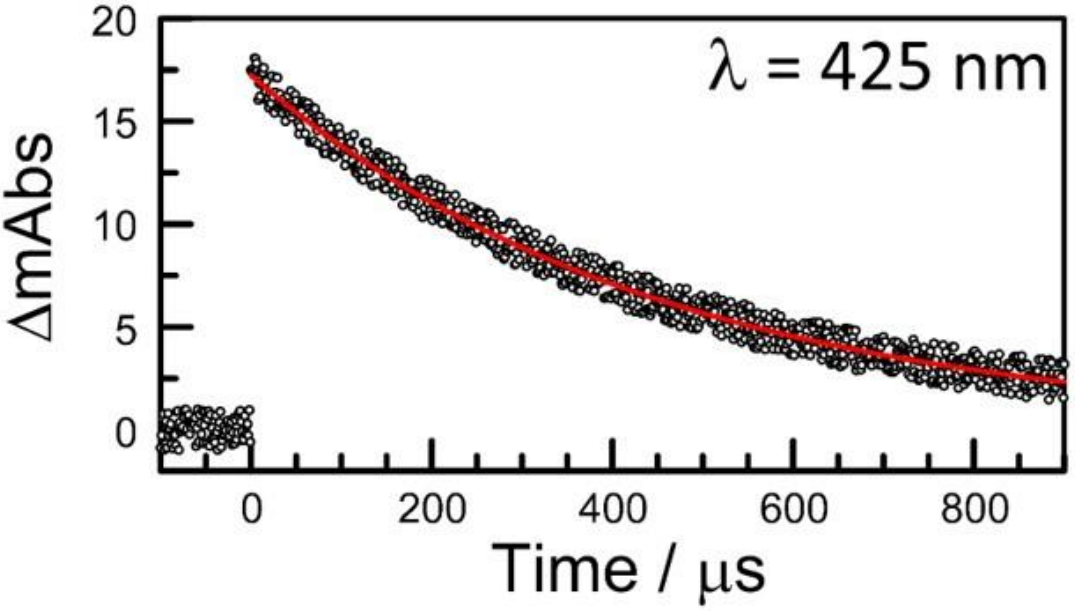

Figure S39. Example of triplet decay curve at 425 nm following laser excitation of **PY-P$_2$** in deoxygenated $CHCl_3$. The red curve passing through the data points corresponds to a first-order kinetic process with a lifetime of 460 μs. The laser intensity was attenuated with metal screen filters until exponential kinetics were obtained.

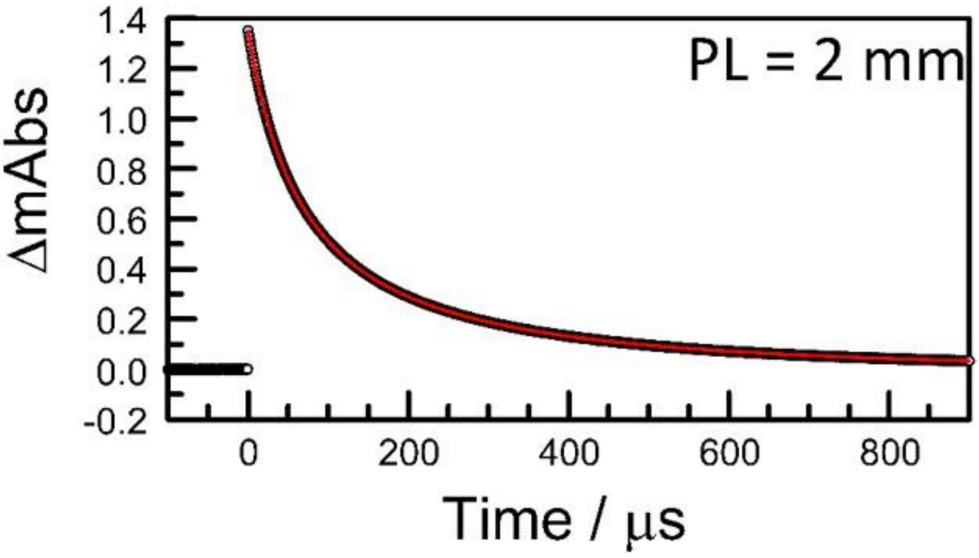

Figure S40. Example of triplet decay curve at 425 nm following laser excitation of **PY-P$_2$** in deoxygenated $CHCl_3$ at high laser intensity. The oprical path length was decreased from 1 cm to 2 mm. The red curve passing through the data points corresponds to a fit to mixed first- and second-order kinetics. The first-order kinetic process corresponds to a lifetime of 445 μs. The second-order process corresponds to a bimolecular rate constant of 1.5×10^8 M^{-1} s^{-1}.

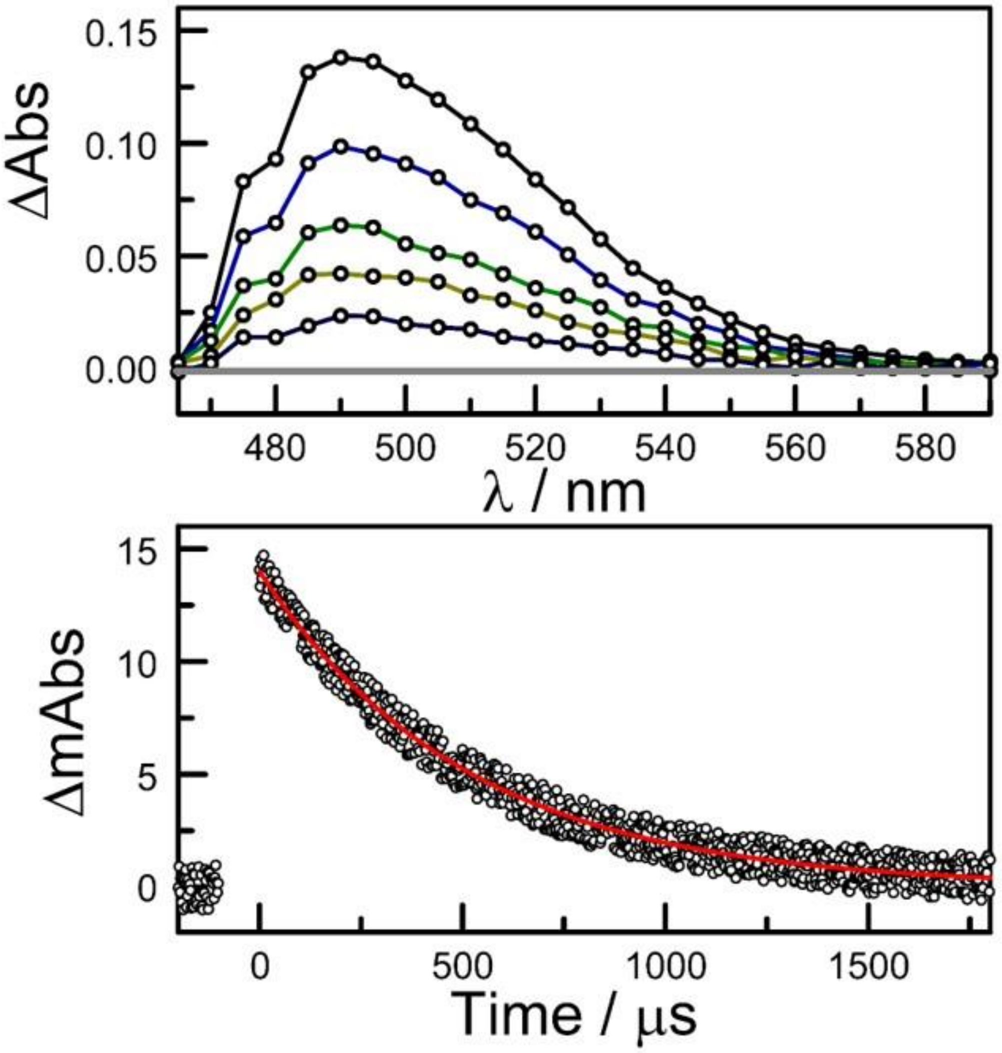

Figure S41. Example of transient differential absorption spectra recorded following excitation of **PY-P$_8$-PER** in deoxygenated $CHCl_3$ with a 4-ns laser pulse at 440 nm. The spectra are assigned to the triplet-excited state of perylene. Spectra were recorded at different times after the excitation pulse. The lower panel shows a decay trace measured at 500 nm, after attenuation of the laser intensity with metal screen filters. The red curve passing through the experimental points corresponds to a first-order process with a lifetime of 510 μs.

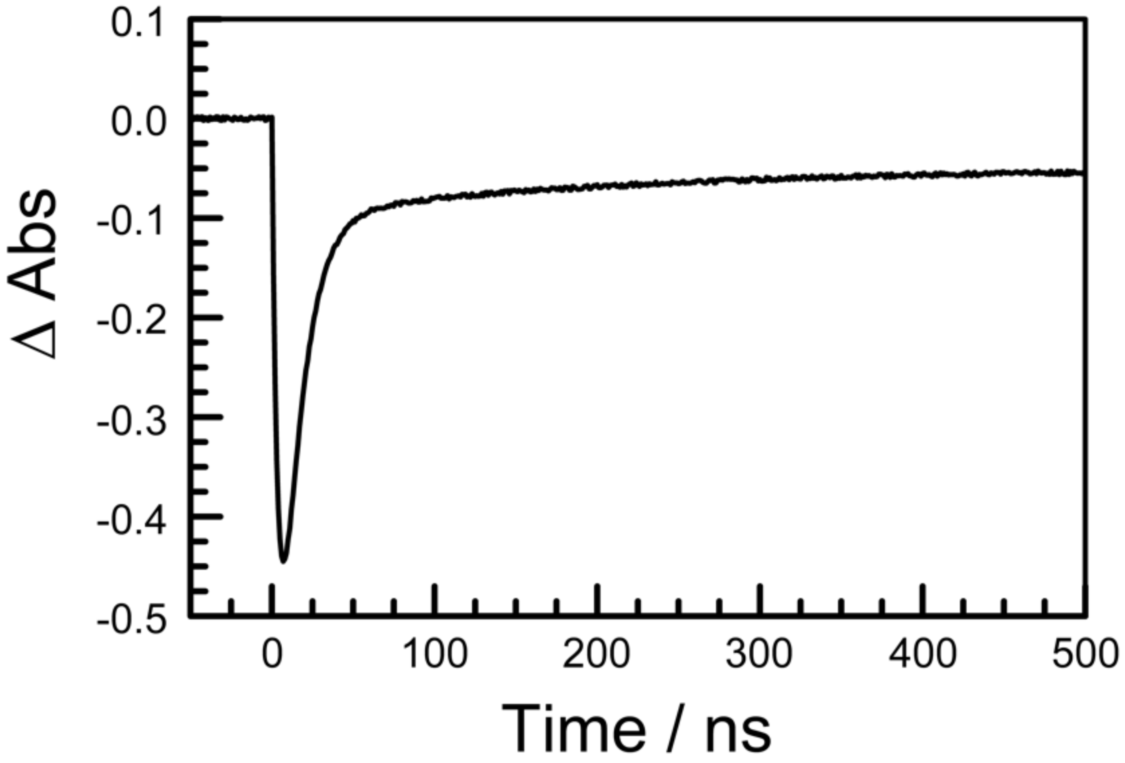

Figure S42. Example of an experimental trace illustrating P-type delayed fluorescence observed for the perylene component of **PY-P_8-PER** in deoxygenated $CHCl_3$ at room temperature. The sample was illuminated with 4-ns laser pulses delivered at 440 nm. The trace contains contributions from both spontaneous emission, decaying with an instrumentally broadened lifetime and delayedfluorescence decaying on a much slower timescale.

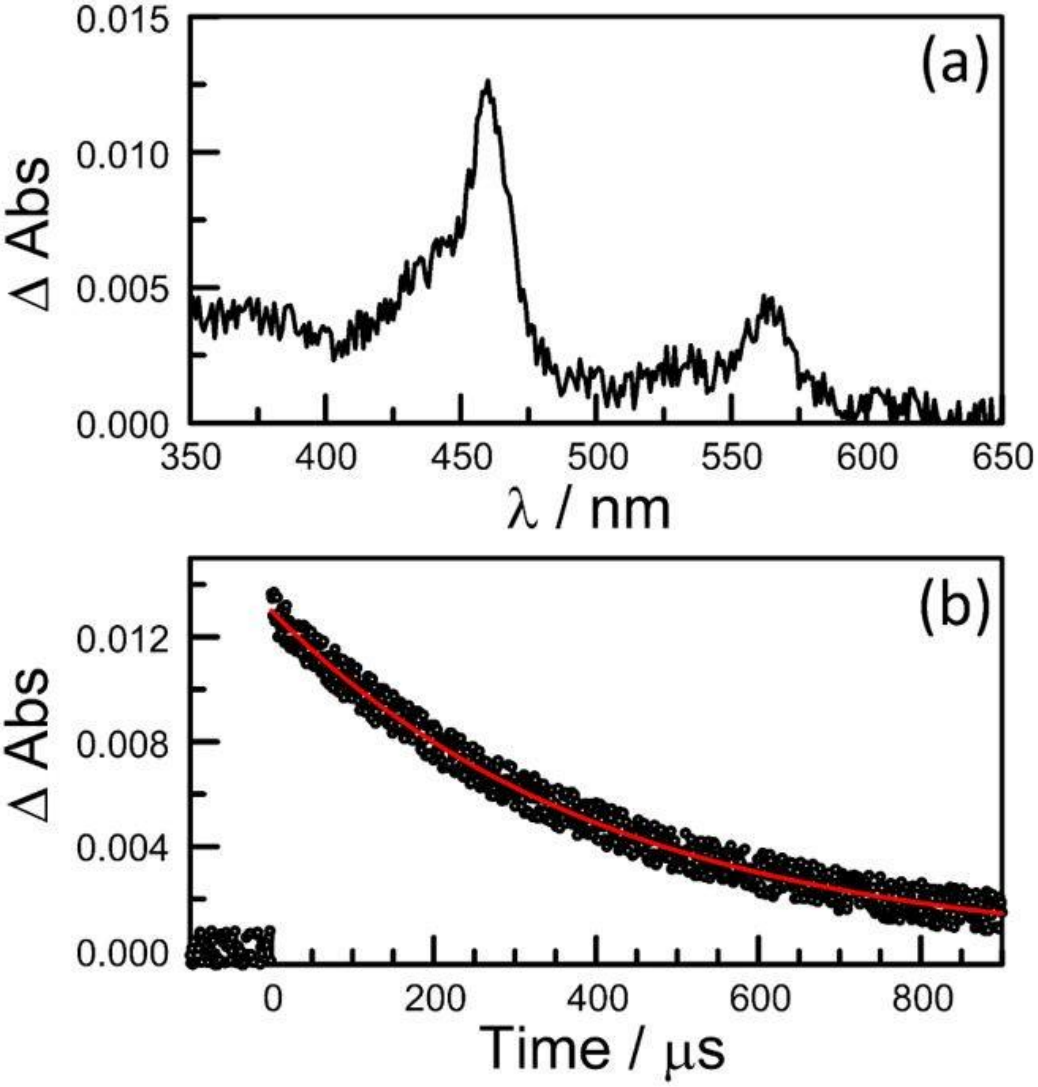

Figure S43. (a) Differential absorption spectrum recorded 100 ns after laser excitation (FWHM = 4 ns) of **PY-P$_8$-PER** in deoxygenated $CHCl_3$, delivered at 340 nm. The spectrum is the average of 100 individual traces. (b) Decay recorded measured at 425 nm for an experiment using metal screen filters to decrease the intensity of the laser pulse. The red curve passing through the experimental points is a least-squares fit to a first-order process having a lifetime of 415 μs.

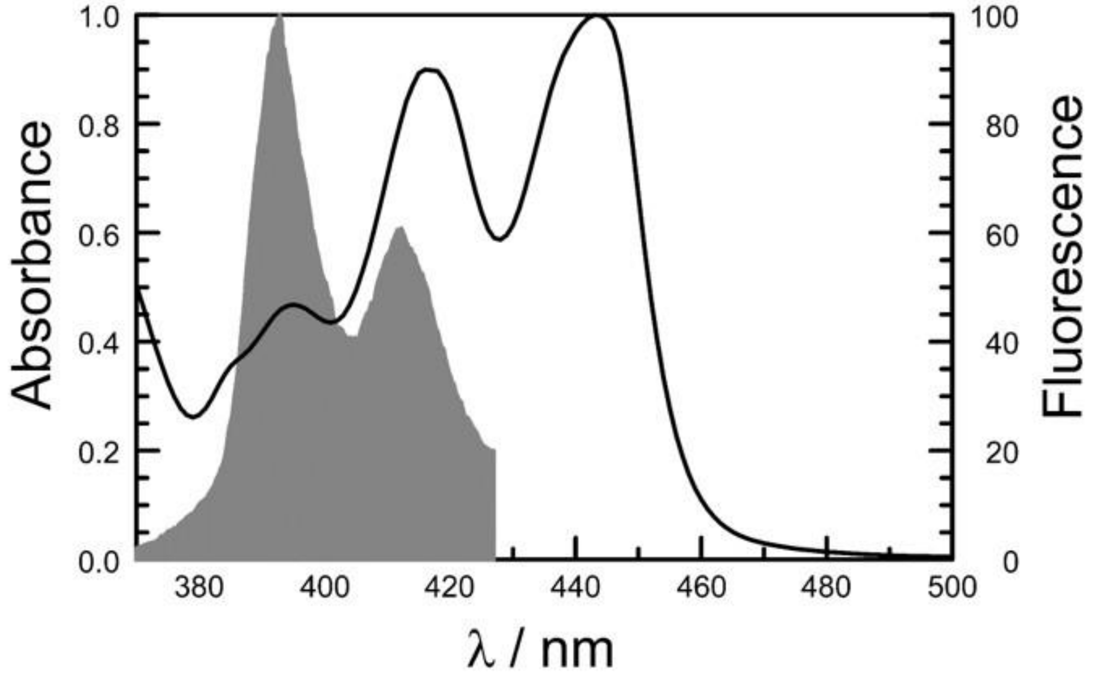

Figure S44. Illustration of spectral overlap between fluorescence from pyrene (grey shaded curve) and absorption by perylene (black curve) for **PY-P$_8$-PER** in $CHCl_3$. The emission spectrum was truncated to avoid fluorescence from perylene. For calculation of the overlap integral, the emission spectrum of **PY-P$_2$** was used in place of the above and was shifted slightly to optimise spectral matching.

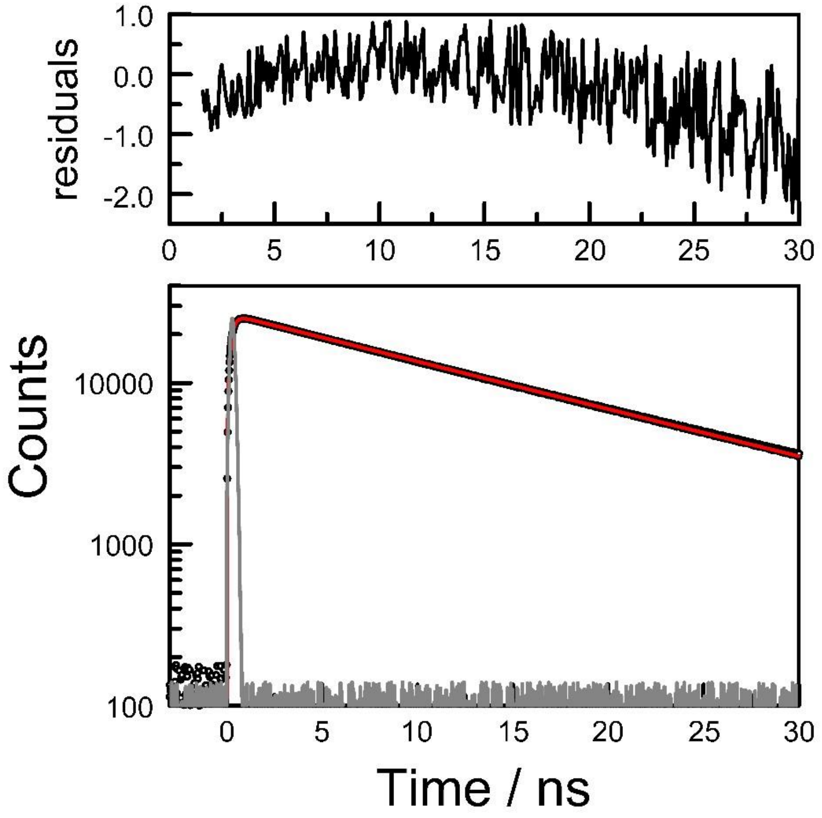

Figure S45. Example of time-resolved fluorescence decay profile recorded for **PY-P$_2$** in deoxygenated $CHCl_3$ following excitation at 330 nm. The instrumental response function is shown as a grey curve while the experimental data are shown as open circes. The non-linear, least-squares fit to a single exponential decay (τ_S = 14.5 ns) is shown as a red curve superimposed over the experimental data (χ^2 = 1.33). The upper panel shows the reduced weighted residuals for the fit.

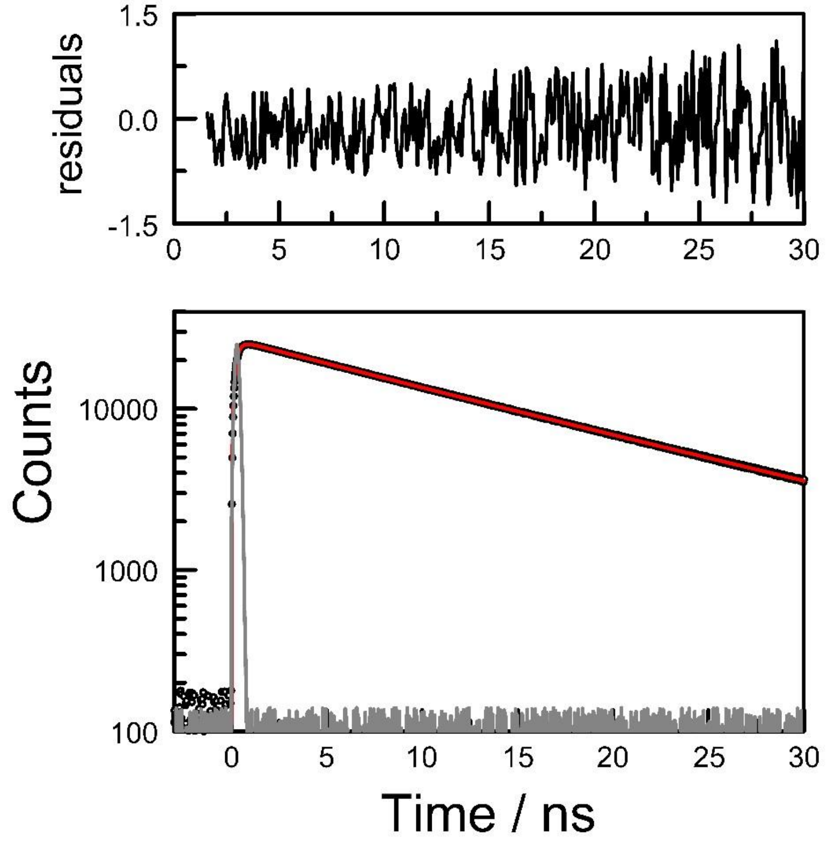

Figure S46. Example of time-resolved fluorescence decay profile recorded for **PY-P$_2$** in deoxygenated $CHCl_3$ following excitation at 330 nm. The instrumental response function is shown as a grey curve while the experimental data are shown as open circes. The non-linear, least-squares fit to a dual exponential decay (τ_S = 11.2 ns (37%); 16.0 ns (63%)) is shown as a red curve superimposed over the experimental data (χ^2 = 1.16). The upper panel shows the reduced weighted residuals for the fit.

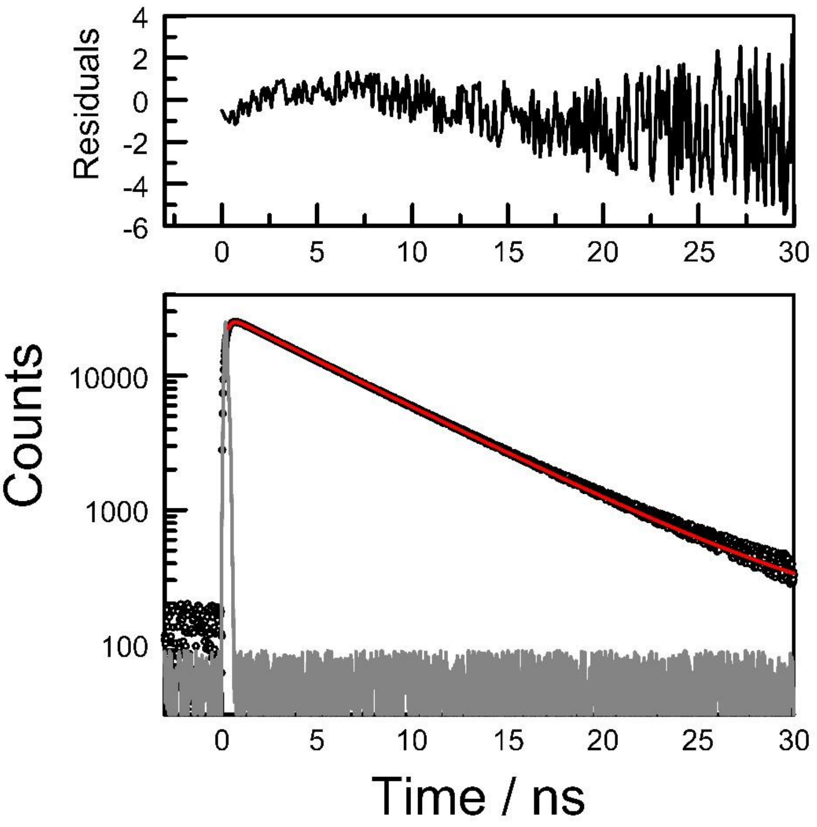

Figure S47. Example of time-resolved fluorescence decay profile recorded for **PY-P$_8$-PER** in deoxygenated $CHCl_3$ following excitation at 440 nm. The instrumental response function is shown as a grey curve while the experimental data are shown as open circes. The non-linear, least-squares fit to a single exponential decay (τ_S = 6.2 ns) is shown as a red curve superimposed over the experimental data (χ^2 = 1.43). The upper panel shows the reduced weighted residuals for the fit.

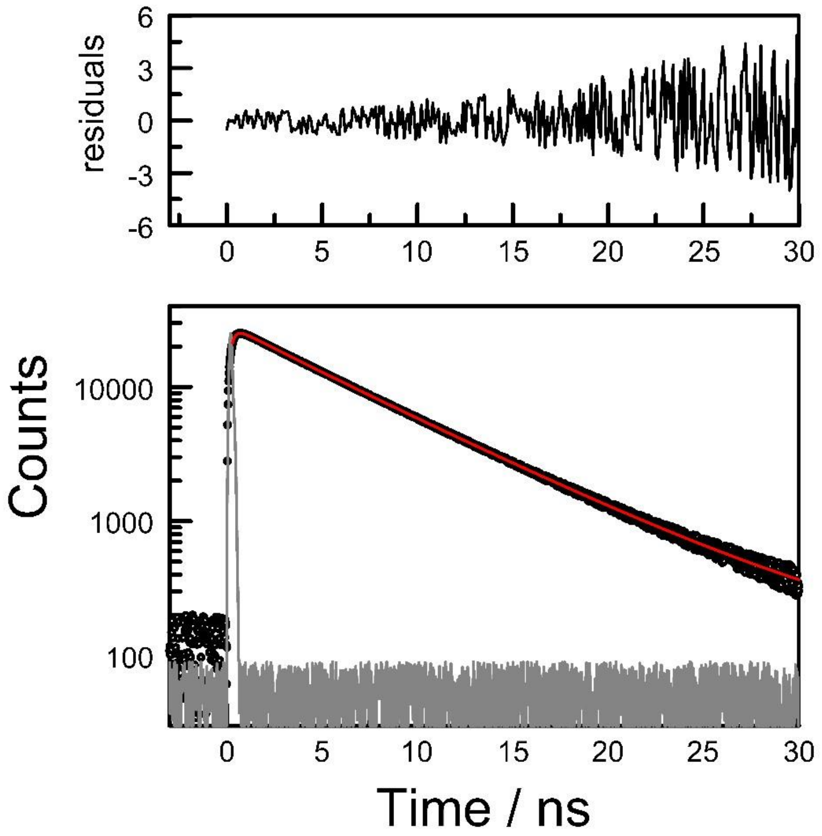

Figure S48. Example of time-resolved fluorescence decay profile recorded for **PY-P8-PER** in deoxygenated $CHCl_3$ following excitation at 440 nm. The instrumental response function is shown as a grey curve while the experimental data are shown as open circes. The non-linear, least-squares fit to a dual exponential decay (τ_S = 4.3 ns (19%); 6.6 ns (81%)) is shown as a red curve superimposed over the experimental data (χ^2 = 1.27). The upper panel shows the reduced weighted residuals for the fit.

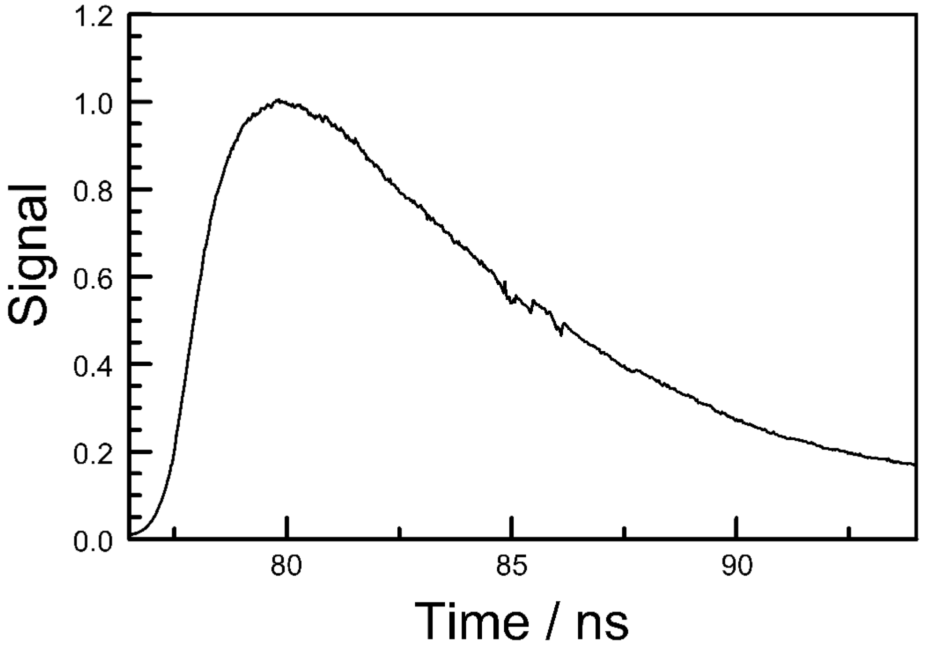

Figure S49. Example of a time-resolved fluorescence decay profile recorded for **PY-P_8-PER** in deoxygenated $CHCl_3$ following indirect excitation at 310 with a low intensity LED. Emission from the perylene component was isolated with a narrow bandpass filter centred at 475 nm. The decay kinetics are considered to be complex since both pyrene and perylene absorb at the excitation wavelength.

S4. Oligoproline Helicity by Circular Dichroism

The conformation of the oligoproline assembly **PY-P_8-PER** was studied with an Aviv model 62DS circular dichromic (CD) spectropolarimeter (Aviv Associates, Lakewood, NJ) using 0.1 cm path length cell and Concentration was 0.25mM **PY-P_8-PER**. The observed ellipticity [θ] (in deg cm^2 $dmo1^{-1}$) was measured at integral values of wavelength (nm) over the range of 190-300 nm using an integration time of 4 s per data point and an acquisition time of about 6 min per spectrum. In prior studies, it was demonstrated[S12,S13] that a PPI helix displays a negative CD band at 199 nm, with a stronger positive band at 205-215 nm. In contrast, the PPII helix has a strong negative CD band at 205-210 nm and a weak positive band at 226 nm. The PPII helix is more favoured in polar solvents.[S13] To probe the helicity of the oligoproline **PY-P_8-PER** CD spectra were recorded in methanol. Spectra were collected after initial preparation of the solution and after leaving for around 12 hours.

The CD spectrum for fresh sample of **PY-P_8-PER** in methanol is illustrated in Figure S48. In addition, showed a strong negative band at around 212 nm, which is consistent with dominance in solution of the PPII helix. This finding is in agreement with previous studies demonstrating[S13] the stabilization of the PPII helix in a polar protic solvent. Over time, the negative band diminished in intensity and was accompanied by a positive band in the 450 nm to 550 nm region, which might be associated with the perylene chromophore.

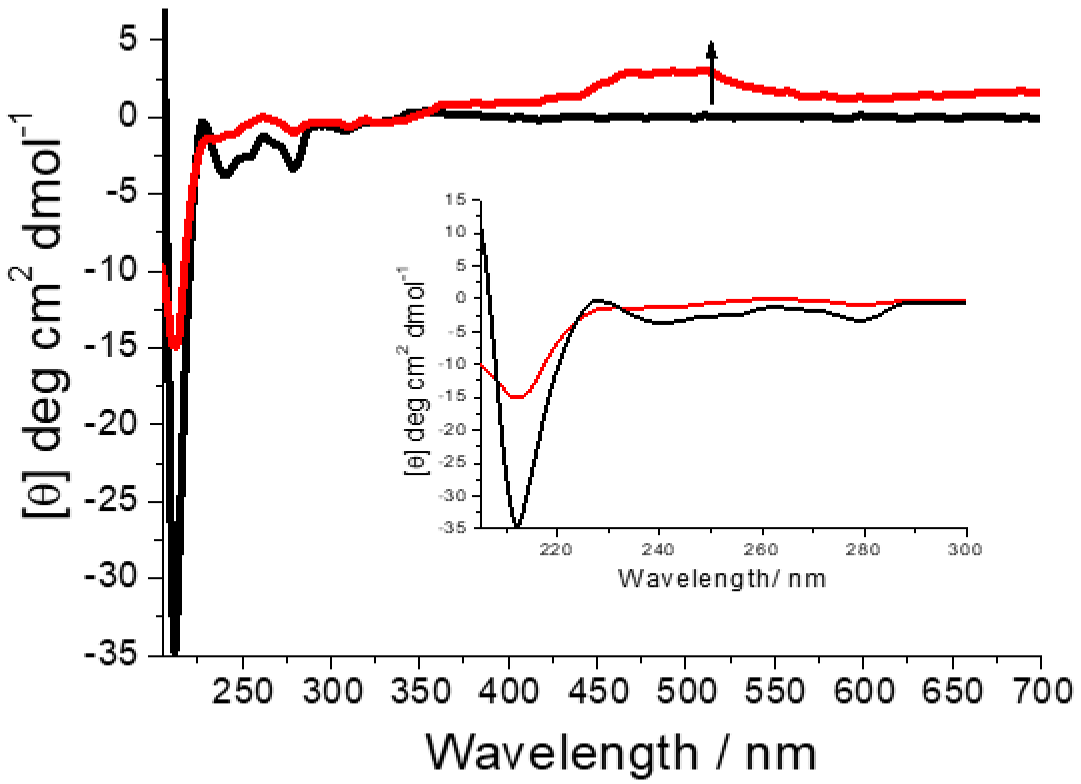

Figure S50. Circular dichroism spectra recorded for **PY-P$_8$-PER** (conc. = 0.25mM) in methanol. The spectrum recorded for a fresh sample is shown as a black curve while an aged sample is shown in red. The insert shows an expansion of the region from 190 to 300 nm.

S5. FRET orientation factor

The orientation factor for dipole-dipole EET was calculated from Equation S3, where ϕ_A and ϕ_D, respectively, refer to the angle tended between the molecular axis and the transition dipole moment vectors localised on acceptor and donor. The latter follow the long molecular axis for perylene[S14] and the short molecular axis for pyrene.[S15] To obtain the require angles, a plane was established for each polycycle as indicated by Figure S51 using Olex-2.[S2] The distance between the centres of these two planes was also obtained using Olex-2 and was equated to the Förster[S16] centre-to-centre separation distance, r. The molecular axis was taken as a straight line connecting these two centres. This allowed straightforward determination of the respective angles.

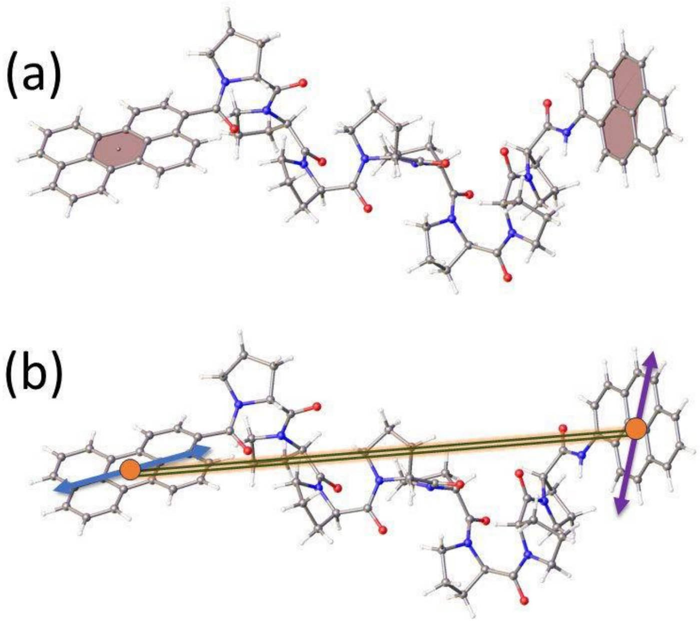

Figure S51. (a) Establishing the planes defining the centres of the terminal polycycles. The planes are coloured pinkish brown. (b) Joining the centres to define the molecular axis (yellow line) and adding the directions of the transition dipole moment vectors for donor (purple line) and acceptor (blue line).

$$\kappa\kappa = \cos(\alpha\alpha) - 3\cos(\phi\phi_{AA})\cos(\phi\phi_{DD}) \qquad (SS3)$$

Manipulation of Equation S3 requires quantitative knowledge of α, which refers to the angle between the two transition dipole moment vectors. This angle is difficult to determine for a 3D structure with widely spaced reactants and considerable internal structural

variations. Two approaches were used: Firstly, the 3D structure was cropped to remove the oligo-proline chain but without affecting the mutual orientation of the polycyclic terminals. One terminal was moved, without affecting the orientation, until adjacent or superimposed above the second terminal. This allowed the angle α to be obtained with a projector (Figure S52). The second method was used in cases where it was difficult to re-align the two polycycles. Here, the cartesian coordinates for the carbon atoms at each end of the transition dipole moment vectors were identified from the structure and used to define the vector in 3D space. The vectors were extended until crossing, allowing access to the required angle.

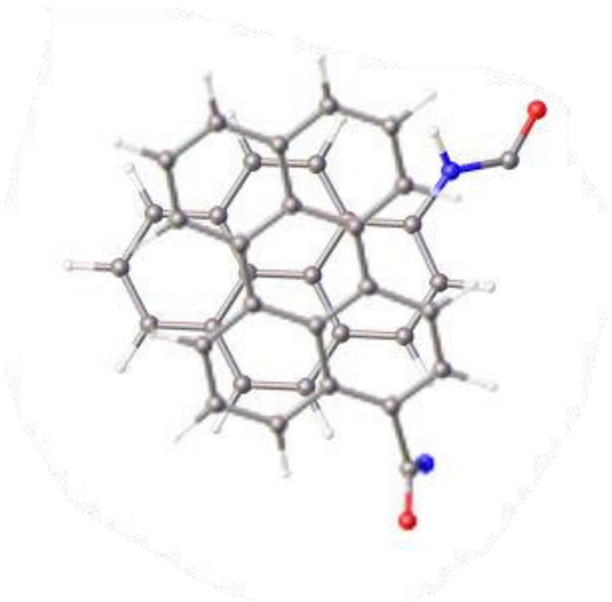

Figure S52. Example of cropping the terminal polycycles from the 3D structure and overlaying the two chromophores without disrupting the calculated mutual orientation. The respective transition dipole moment vectors can now be added and α determined with a projector.

S6. Computational studies

Initial estimates for the structure of **PY-P₂** in the gas phase were made using Spartan with the MMFF94 force field.[S17] The geometry around the pyrene-connected amide bond was constrained to be either *cis* or *trans*. No other restrictions were applied. Output from these calculations was used as primary input for more precise structure determinations made at the DFT level using the GAMESS program suite.[S18] The PBE0 hybrid functional[S19] was used together with the 6-311(G,d) basis set.[S20] The solute was held in a reservoir of $CHCl_3$ molecules using the PCM treatment[S21] with the Mennucci-Thomasi correction.[S22] Vibrational spectra were calculated for the optimised geometries and the derived cartesian coordinates are provided below. All optimised geometries were further examined through vibrational frequency analysis to find the local minimum energy of each conformer. Torsional potentials for twisting around the amide bond were evaluated by internal rotation from 0° to 360° with a 5° increment. The geometry of each structure was fully optimized at the B3LYP/6-311G** level[S23] during the rotational calculations, again using the PCM model for the solvent. After geometric optimisation, the internal rotational barriers were calculated from the maximum energy difference along the respective coordinate. The second amide bond was not considered in these calculations.

Table S2. Cartesian coordinates for the *trans*-isomer of **PY-P$_2$**.

C1	C	0.22557	-0.52009	0.45975	1.00000	0.05000
C2	C	0.24399	-0.48797	0.34025	1.00000	0.05000
C3	C	0.32780	-0.50785	0.28429	1.00000	0.05000
C4	C	0.39110	-0.55778	0.35401	1.00000	0.05000
C5	C	0.37083	-0.58843	0.47534	1.00000	0.05000
C6	C	0.28802	-0.56971	0.52634	1.00000	0.05000
C7	C	0.47612	-0.57750	0.30388	1.00000	0.05000
C8	C	0.53907	-0.62619	0.37470	1.00000	0.05000
C9	C	0.51688	-0.65552	0.49431	1.00000	0.05000
C10	C	0.43365	-0.63703	0.54388	1.00000	0.05000
C11	C	0.35333	-0.48064	0.16301	1.00000	0.05000
C12	C	0.43660	-0.50040	0.11351	1.00000	0.05000
C13	C	0.49869	-0.54826	0.18301	1.00000	0.05000
C14	C	0.58277	-0.56731	0.13462	1.00000	0.05000
C15	C	0.64453	-0.61483	0.20499	1.00000	0.05000
C16	C	0.62280	-0.64417	0.32428	1.00000	0.05000
N1	N	0.18119	-0.43578	0.27321	1.00000	0.05000

C17	C	0.10111	-0.40058	0.31420	1.00000	0.05000
O1	O	0.06837	-0.40999	0.41798	1.00000	0.05000
C18	C	0.05096	-0.35159	0.20805	1.00000	0.05000
H1	H	0.20052	-0.41444	0.18844	1.00000	0.05000
C19	C	-0.01353	-0.41706	0.14523	1.00000	0.05000
C20	C	-0.10013	-0.40021	0.21427	1.00000	0.05000
C21	C	-0.09797	-0.29890	0.23863	1.00000	0.05000
N2	N	-0.00475	-0.27815	0.25356	1.00000	0.05000
C22	C	0.03281	-0.19632	0.28592	1.00000	0.05000
C23	C	-0.03041	-0.12096	0.33149	1.00000	0.05000
O2	O	0.11356	-0.18572	0.27972	1.00000	0.05000
C24	C	-0.06346	-0.14032	0.46364	1.00000	0.05000
C25	C	0.00492	-0.09375	0.54612	1.00000	0.05000
C26	C	0.02835	-0.00916	0.47304	1.00000	0.05000
N3	N	0.01468	-0.03402	0.34433	1.00000	0.05000
C27	C	0.04163	0.02044	0.24852	1.00000	0.05000
O3	O	0.08027	0.09227	0.26426	1.00000	0.05000
O4	O	0.01961	-0.01476	0.13597	1.00000	0.05000
C28	C	0.04971	0.02816	0.02225	1.00000	0.05000
C29	C	0.15055	0.02234	0.01158	1.00000	0.05000
C30	C	0.01645	0.12576	0.01009	1.00000	0.05000
C31	C	0.00833	-0.02831	-0.08266	1.00000	0.05000
H2	H	0.16259	-0.50763	0.50489	1.00000	0.05000
H3	H	0.27104	-0.59298	0.61935	1.00000	0.05000
H4	H	0.56422	-0.69298	0.55035	1.00000	0.05000
H5	H	0.41852	-0.66071	0.63733	1.00000	0.05000
H6	H	0.30917	-0.44284	0.10289	1.00000	0.05000
H7	H	0.45230	-0.47732	0.01998	1.00000	0.05000
H8	H	0.60088	-0.54487	0.04168	1.00000	0.05000
H9	H	0.70963	-0.62891	0.16694	1.00000	0.05000
H10	H	0.67189	-0.68112	0.37795	1.00000	0.05000
H11	H	0.09840	-0.32451	0.14055	1.00000	0.05000
H12	H	-0.02166	-0.39862	0.04706	1.00000	0.05000
H13	H	0.00692	-0.48799	0.14982	1.00000	0.05000
H14	H	-0.15822	-0.42111	0.16107	1.00000	0.05000
H15	H	-0.10015	-0.43730	0.30265	1.00000	0.05000
H16	H	-0.12226	-0.26090	0.15808	1.00000	0.05000
H17	H	-0.13634	-0.27958	0.32041	1.00000	0.05000
H18	H	-0.08476	-0.11403	0.26490	1.00000	0.05000
H19	H	-0.12815	-0.10841	0.47749	1.00000	0.05000
H20	H	-0.06937	-0.21211	0.48654	1.00000	0.05000
H21	H	-0.02014	-0.07853	0.63922	1.00000	0.05000
H22	H	0.06327	-0.13713	0.55589	1.00000	0.05000
H23	H	-0.01681	0.04660	0.49471	1.00000	0.05000
H24	H	0.09625	0.01348	0.48883	1.00000	0.05000
H25	H	0.17358	0.04647	-0.07914	1.00000	0.05000

H26	H	0.17312	-0.04752	0.02418	1.00000	0.05000
H27	H	0.18359	0.06267	0.08320	1.00000	0.05000
H28	H	0.02733	0.15202	-0.08401	1.00000	0.05000
H29	H	-0.05421	0.13000	0.03138	1.00000	0.05000
H30	H	0.05032	0.17173	0.07404	1.00000	0.05000
H31	H	0.02757	-0.00335	-0.17470	1.00000	0.05000
H32	H	-0.06397	-0.02719	-0.07644	1.00000	0.05000
H33	H	0.02808	-0.09946	-0.07525	1.00000	0.05000

Table S3. Cartesian coordinates for the *cis*-isomer of **PY-P$_2$**.

C1	C	-0.26202	-0.34637	0.39011	1.00000	0.05000
C2	C	-0.31997	-0.31316	0.31817	1.00000	0.05000
C3	C	-0.40771	-0.27561	0.34372	1.00000	0.05000
C4	C	-0.43013	-0.26414	0.44098	1.00000	0.05000
C5	C	-0.36816	-0.29383	0.51168	1.00000	0.05000
C6	C	-0.28548	-0.33702	0.48524	1.00000	0.05000
C7	C	-0.51483	-0.22166	0.46959	1.00000	0.05000
C8	C	-0.53509	-0.20708	0.56659	1.00000	0.05000
C9	C	-0.47165	-0.23603	0.63460	1.00000	0.05000
C10	C	-0.38919	-0.27971	0.60739	1.00000	0.05000
C11	C	-0.47541	-0.24852	0.27788	1.00000	0.05000
C12	C	-0.55906	-0.20842	0.30584	1.00000	0.05000
C13	C	-0.57925	-0.19332	0.40119	1.00000	0.05000
C14	C	-0.66239	-0.15091	0.42989	1.00000	0.05000
C15	C	-0.68169	-0.13582	0.52537	1.00000	0.05000
C16	C	-0.61844	-0.16370	0.59339	1.00000	0.05000
N1	N	-0.29510	-0.30474	0.22176	1.00000	0.05000
C17	C	-0.22298	-0.32818	0.16726	1.00000	0.05000
O1	O	-0.22550	-0.28628	0.08619	1.00000	0.05000
C18	C	-0.14571	-0.40861	0.19720	1.00000	0.05000
H1	H	-0.33779	-0.25753	0.18104	1.00000	0.05000
C19	C	-0.16186	-0.53666	0.16128	1.00000	0.05000
C20	C	-0.11152	-0.54054	0.06788	1.00000	0.05000
C21	C	-0.03021	-0.46509	0.08847	1.00000	0.05000
N2	N	-0.06074	-0.37461	0.15451	1.00000	0.05000
C22	C	-0.00513	-0.28200	0.18135	1.00000	0.05000
C23	C	-0.04480	-0.18112	0.24365	1.00000	0.05000
O2	O	0.07312	-0.28114	0.15689	1.00000	0.05000
C24	C	-0.04322	-0.21345	0.34891	1.00000	0.05000
C25	C	0.03915	-0.15071	0.38674	1.00000	0.05000
C26	C	0.04278	-0.03604	0.32925	1.00000	0.05000
N3	N	0.00606	-0.06986	0.23901	1.00000	0.05000
C27	C	0.01311	0.00158	0.16061	1.00000	0.05000
O3	O	0.05124	0.09834	0.16032	1.00000	0.05000

O4	O	-0.02571	-0.05013	0.08455	1.00000	0.05000
C28	C	-0.01281	-0.00259	-0.00962	1.00000	0.05000
C29	C	0.08549	0.00049	-0.03597	1.00000	0.05000
C30	C	-0.05661	0.12033	-0.02058	1.00000	0.05000
C31	C	-0.05964	-0.09262	-0.07478	1.00000	0.05000
H2	H	-0.19548	-0.37636	0.37855	1.00000	0.05000
H3	H	-0.23704	-0.36148	0.53874	1.00000	0.05000
H4	H	-0.48575	-0.22514	0.70945	1.00000	0.05000
H5	H	-0.34130	-0.30175	0.66203	1.00000	0.05000
H6	H	-0.46557	-0.25999	0.20237	1.00000	0.05000
H7	H	-0.60814	-0.18904	0.25175	1.00000	0.05000
H8	H	-0.71268	-0.12886	0.37781	1.00000	0.05000
H9	H	-0.74610	-0.10236	0.54685	1.00000	0.05000
H10	H	-0.63468	-0.15136	0.66762	1.00000	0.05000
H11	H	-0.13531	-0.40999	0.27234	1.00000	0.05000
H12	H	-0.13256	-0.60147	0.21045	1.00000	0.05000
H13	H	-0.23174	-0.55935	0.15253	1.00000	0.05000
H14	H	-0.09497	-0.63164	0.04549	1.00000	0.05000
H15	H	-0.15102	-0.49867	0.01191	1.00000	0.05000
H16	H	0.02127	-0.51812	0.12339	1.00000	0.05000
H17	H	-0.00232	-0.42335	0.02516	1.00000	0.05000
H18	H	-0.11186	-0.16128	0.21953	1.00000	0.05000
H19	H	-0.10175	-0.17592	0.38375	1.00000	0.05000
H20	H	-0.04090	-0.30964	0.36338	1.00000	0.05000
H21	H	0.03601	-0.13402	0.46280	1.00000	0.05000
H22	H	0.09757	-0.20588	0.37230	1.00000	0.05000
H23	H	0.00017	0.03340	0.35969	1.00000	0.05000
H24	H	0.10965	-0.00015	0.32162	1.00000	0.05000
H25	H	0.09478	0.02134	-0.11095	1.00000	0.05000
H26	H	0.11695	-0.08598	-0.02100	1.00000	0.05000
H27	H	0.12174	0.06801	0.00459	1.00000	0.05000
H28	H	-0.05547	0.15031	-0.09428	1.00000	0.05000
H29	H	-0.12543	0.11749	0.00336	1.00000	0.05000
H30	H	-0.02349	0.18902	0.02175	1.00000	0.05000
H31	H	-0.05422	-0.06689	-0.14922	1.00000	0.05000
H32	H	-0.12985	-0.10002	-0.05670	1.00000	0.05000
H33	H	-0.03160	-0.18267	-0.06634	1.00000	0.05000

Table S4. Cartesian coordinates for the all-*trans*-isomer of **PY-P_8-PER**.

C1	C	-0.71548	-0.54490	-1.37017	1.00000	0.05000
C2	C	-0.75340	-0.59624	-1.34731	1.00000	0.05000
C3	C	-0.78153	-0.57192	-1.28043	1.00000	0.05000
C4	C	-0.77159	-0.49475	-1.23643	1.00000	0.05000

C5	C	-0.73316	-0.44347	-1.25985	1.00000	0.05000
C6	C	-0.70522	-0.46895	-1.32657	1.00000	0.05000
C7	C	-0.80002	-0.46879	-1.16944	1.00000	0.05000
C8	C	-0.79068	-0.39046	-1.12586	1.00000	0.05000
C9	C	-0.75144	-0.34189	-1.14997	1.00000	0.05000
C10	C	-0.72324	-0.36759	-1.21585	1.00000	0.05000
C11	C	-0.81941	-0.62336	-1.25678	1.00000	0.05000
C12	C	-0.84731	-0.59860	-1.19019	1.00000	0.05000
C13	C	-0.83796	-0.52180	-1.14676	1.00000	0.05000
C14	C	-0.86616	-0.49687	-1.08071	1.00000	0.05000
C15	C	-0.85746	-0.41944	-1.03874	1.00000	0.05000
C16	C	-0.82045	-0.36425	-1.06016	1.00000	0.05000
N1	N	-0.81428	-0.28190	-1.01837	1.00000	0.05000
C17	C	-0.83837	-0.24685	-0.94814	1.00000	0.05000
O1	O	-0.86585	-0.28843	-0.90066	1.00000	0.05000
C18	C	-0.79771	-0.14146	-0.84365	1.00000	0.05000
C19	C	-0.82563	-0.14752	-0.92803	1.00000	0.05000
N2	N	-0.79457	-0.10229	-0.99137	1.00000	0.05000
C20	C	-0.74851	-0.07962	-0.95344	1.00000	0.05000
C21	C	-0.74545	-0.13504	-0.87206	1.00000	0.05000
C22	C	-0.81550	-0.06370	-1.06255	1.00000	0.05000
O2	O	-0.85825	-0.08077	-1.07842	1.00000	0.05000
C23	C	-0.78768	0.00121	-1.12363	1.00000	0.05000
N3	N	-0.73532	0.01422	-1.11730	1.00000	0.05000
C24	C	-0.71074	-0.03424	-1.18553	1.00000	0.05000
C25	C	-0.74934	-0.08970	-1.22955	1.00000	0.05000
C26	C	-0.64355	0.11625	-0.97967	1.00000	0.05000
C27	C	-0.66265	0.11215	-1.07194	1.00000	0.05000
N4	N	-0.62662	0.05642	-1.11575	1.00000	0.05000
C28	C	-0.59861	0.00470	-1.05411	1.00000	0.05000
C29	C	-0.62135	0.02356	-0.96728	1.00000	0.05000
C30	C	-0.61381	0.07610	-1.19925	1.00000	0.05000
C31	C	-0.57708	0.01810	-1.25068	1.00000	0.05000
O3	O	-0.63464	0.13806	-1.23747	1.00000	0.05000
N5	N	-0.54877	-0.05217	-1.20904	1.00000	0.05000
C32	C	-0.49861	-0.02476	-1.20119	1.00000	0.05000
C33	C	-0.49664	0.07233	-1.23174	1.00000	0.05000
C34	C	-0.53915	0.07877	-1.29260	1.00000	0.05000
C35	C	-0.71707	0.08515	-1.07027	1.00000	0.05000
O4	O	-0.74469	0.12949	-1.02431	1.00000	0.05000
C36	C	-0.56770	-0.13773	-1.20181	1.00000	0.05000
O5	O	-0.60916	-0.15299	-1.22772	1.00000	0.05000
C37	C	-0.53300	-0.29218	-1.22663	1.00000	0.05000
C38	C	-0.53951	-0.21687	-1.16043	1.00000	0.05000
N6	N	-0.49140	-0.20295	-1.12492	1.00000	0.05000
C39	C	-0.45558	-0.25133	-1.17490	1.00000	0.05000

C40	C	-0.48131	-0.28031	-1.25610	1.00000	0.05000
C41	C	-0.48655	-0.16647	-1.04406	1.00000	0.05000
C42	C	-0.43666	-0.15091	-1.00135	1.00000	0.05000
O6	O	-0.52304	-0.14484	-1.00314	1.00000	0.05000
N7	N	-0.39255	-0.16487	-1.05044	1.00000	0.05000
C43	C	-0.36693	-0.24366	-1.01971	1.00000	0.05000
C44	C	-0.40091	-0.28983	-0.95721	1.00000	0.05000
C45	C	-0.43113	-0.21248	-0.92333	1.00000	0.05000
C46	C	-0.29384	-0.02886	-1.13063	1.00000	0.05000
C47	C	-0.32790	-0.10626	-1.15530	1.00000	0.05000
N8	N	-0.29772	-0.18739	-1.15227	1.00000	0.05000
C48	C	-0.24974	-0.16507	-1.12189	1.00000	0.05000
C49	C	-0.25537	-0.07492	-1.07737	1.00000	0.05000
C50	C	-0.30915	-0.25939	-1.20453	1.00000	0.05000
C51	C	-0.28200	-0.35126	-1.19967	1.00000	0.05000
O7	O	-0.34482	-0.25443	-1.25214	1.00000	0.05000
N9	N	-0.23816	-0.36735	-1.14890	1.00000	0.05000
C52	C	-0.20149	-0.41010	-1.20192	1.00000	0.05000
C53	C	-0.21435	-0.38021	-1.29191	1.00000	0.05000
C54	C	-0.26905	-0.38305	-1.29061	1.00000	0.05000
C55	C	-0.37545	-0.09759	-1.10299	1.00000	0.05000
O8	O	-0.39907	-0.02724	-1.11118	1.00000	0.05000
C56	C	-0.15828	-0.37151	-1.01990	1.00000	0.05000
C57	C	-0.12063	-0.39143	-0.96933	1.00000	0.05000
C58	C	-0.12571	-0.44147	-0.89702	1.00000	0.05000
C59	C	-0.17092	-0.47273	-0.87732	1.00000	0.05000
C60	C	-0.20886	-0.45694	-0.93251	1.00000	0.05000
C61	C	-0.20223	-0.40593	-1.00412	1.00000	0.05000
C62	C	-0.17869	-0.51869	-0.80207	1.00000	0.05000
C63	C	-0.22389	-0.54969	-0.78575	1.00000	0.05000
C64	C	-0.11067	-0.58424	-0.61363	1.00000	0.05000
C65	C	-0.25273	-0.49169	-0.91359	1.00000	0.05000
C66	C	-0.08786	-0.45900	-0.84317	1.00000	0.05000
C67	C	-0.09620	-0.50259	-0.76719	1.00000	0.05000
C68	C	-0.14152	-0.53135	-0.74575	1.00000	0.05000
C69	C	-0.05879	-0.51722	-0.71169	1.00000	0.05000
C70	C	-0.06623	-0.55765	-0.63549	1.00000	0.05000
C71	C	-0.26019	-0.53725	-0.84096	1.00000	0.05000
C72	C	-0.14757	-0.57161	-0.66800	1.00000	0.05000
C73	C	-0.04166	-0.43430	-0.86128	1.00000	0.05000
C74	C	-0.00515	-0.45065	-0.80695	1.00000	0.05000
C75	C	-0.01363	-0.49145	-0.73197	1.00000	0.05000
C76	C	-0.24355	-0.37656	-1.06123	1.00000	0.05000
O9	O	-0.28241	-0.35613	-1.02825	1.00000	0.05000
C77	C	-0.79412	-0.03243	-1.21599	1.00000	0.05000
H1	H	-0.69302	-0.56474	-1.42395	1.00000	0.05000

H2	H	-0.76073	-0.65710	-1.38376	1.00000	0.05000
H3	H	-0.67436	-0.42893	-1.34625	1.00000	0.05000
H4	H	-0.74053	-0.28084	-1.11737	1.00000	0.05000
H5	H	-0.69248	-0.32568	-1.23265	1.00000	0.05000
H6	H	-0.82807	-0.68527	-1.29057	1.00000	0.05000
H7	H	-0.87752	-0.64140	-1.17261	1.00000	0.05000
H8	H	-0.89670	-0.53752	-1.06059	1.00000	0.05000
H9	H	-0.88240	-0.40266	-0.98770	1.00000	0.05000
H10	H	-0.79214	-0.23652	-1.04622	1.00000	0.05000
H11	H	-0.80784	-0.07954	-0.80958	1.00000	0.05000
H12	H	-0.80352	-0.19818	-0.80020	1.00000	0.05000
H13	H	-0.85989	-0.11115	-0.92278	1.00000	0.05000
H14	H	-0.74840	-0.00797	-0.93749	1.00000	0.05000
H15	H	-0.71940	-0.09628	-0.99752	1.00000	0.05000
H16	H	-0.72238	-0.10360	-0.82392	1.00000	0.05000
H17	H	-0.73105	-0.20218	-0.88600	1.00000	0.05000
H18	H	-0.80573	0.06643	-1.11653	1.00000	0.05000
H19	H	-0.69618	0.01426	-1.23115	1.00000	0.05000
H20	H	-0.68242	-0.07669	-1.15877	1.00000	0.05000
H21	H	-0.74125	-0.10164	-1.29721	1.00000	0.05000
H22	H	-0.75370	-0.15486	-1.19786	1.00000	0.05000
H23	H	-0.61527	0.16770	-0.97488	1.00000	0.05000
H24	H	-0.67079	0.13082	-0.93100	1.00000	0.05000
H25	H	-0.66229	0.18004	-1.09939	1.00000	0.05000
H26	H	-0.56117	0.02788	-1.05577	1.00000	0.05000
H27	H	-0.60114	-0.06702	-1.06823	1.00000	0.05000
H28	H	-0.59530	0.02113	-0.91475	1.00000	0.05000
H29	H	-0.64955	-0.02609	-0.95375	1.00000	0.05000
H30	H	-0.59866	-0.01308	-1.30137	1.00000	0.05000
H31	H	-0.47628	-0.06855	-1.24130	1.00000	0.05000
H32	H	-0.48729	-0.02796	-1.13456	1.00000	0.05000
H33	H	-0.46234	0.08843	-1.26260	1.00000	0.05000
H34	H	-0.50170	0.11877	-1.17790	1.00000	0.05000
H35	H	-0.52884	0.05097	-1.35512	1.00000	0.05000
H36	H	-0.55046	0.14872	-1.30205	1.00000	0.05000
H37	H	-0.53701	-0.35807	-1.19567	1.00000	0.05000
H38	H	-0.55828	-0.28928	-1.28036	1.00000	0.05000
H39	H	-0.56290	-0.24116	-1.10879	1.00000	0.05000
H40	H	-0.44351	-0.30970	-1.13785	1.00000	0.05000
H41	H	-0.42536	-0.20818	-1.19106	1.00000	0.05000
H42	H	-0.46594	-0.34165	-1.28348	1.00000	0.05000
H43	H	-0.47938	-0.22693	-1.30449	1.00000	0.05000
H44	H	-0.43727	-0.08063	-0.97984	1.00000	0.05000
H45	H	-0.33387	-0.22114	-0.98815	1.00000	0.05000
H46	H	-0.35925	-0.28895	-1.07272	1.00000	0.05000
H47	H	-0.38161	-0.32595	-0.90673	1.00000	0.05000

H48	H	-0.42412	-0.33823	-0.99048	1.00000	0.05000
H49	H	-0.41113	-0.17743	-0.87272	1.00000	0.05000
H50	H	-0.46529	-0.23573	-0.89613	1.00000	0.05000
H51	H	-0.27743	-0.00010	-1.18843	1.00000	0.05000
H52	H	-0.31110	0.02618	-1.09542	1.00000	0.05000
H53	H	-0.33904	-0.09604	-1.22197	1.00000	0.05000
H54	H	-0.22585	-0.16035	-1.17760	1.00000	0.05000
H55	H	-0.23554	-0.21422	-1.07726	1.00000	0.05000
H56	H	-0.22166	-0.03705	-1.07503	1.00000	0.05000
H57	H	-0.26838	-0.08437	-1.01170	1.00000	0.05000
H58	H	-0.30944	-0.39780	-1.17478	1.00000	0.05000
H59	H	-0.20474	-0.48338	-1.19418	1.00000	0.05000
H60	H	-0.16509	-0.38909	-1.18567	1.00000	0.05000
H61	H	-0.19874	-0.42351	-1.34117	1.00000	0.05000
H62	H	-0.20207	-0.31078	-1.30296	1.00000	0.05000
H63	H	-0.28137	-0.45273	-1.29976	1.00000	0.05000
H64	H	-0.28461	-0.34251	-1.34206	1.00000	0.05000
H65	H	-0.15218	-0.32561	-1.07273	1.00000	0.05000
H66	H	-0.08669	-0.36169	-0.98886	1.00000	0.05000
H67	H	-0.23358	-0.58673	-0.72854	1.00000	0.05000
H68	H	-0.11702	-0.61593	-0.55199	1.00000	0.05000
H69	H	-0.28391	-0.48465	-0.95500	1.00000	0.05000
H70	H	-0.03685	-0.56901	-0.59071	1.00000	0.05000
H71	H	-0.29580	-0.56359	-0.82641	1.00000	0.05000
H72	H	-0.18212	-0.59487	-0.64451	1.00000	0.05000
H73	H	-0.03127	-0.40129	-0.92019	1.00000	0.05000
H74	H	0.03125	-0.43030	-0.82357	1.00000	0.05000
H75	H	0.01637	-0.50321	-0.68869	1.00000	0.05000
H76	H	-0.79352	0.02478	-1.26055	1.00000	0.05000
H77	H	-0.82707	-0.07067	-1.22790	1.00000	0.05000

Table S5. Cartesian coordinates for the *cis*-isomer (C1) of **PY-P_8-PER**.

C1	C	-2.01329	-0.25444	-0.40938	1.00000	0.05000
C2	C	-2.04324	-0.19906	-0.42660	1.00000	0.05000
C3	C	-2.00365	-0.17062	-0.47481	1.00000	0.05000
C4	C	-1.93307	-0.19803	-0.50597	1.00000	0.05000
C5	C	-1.90322	-0.25398	-0.48836	1.00000	0.05000
C6	C	-1.94374	-0.28186	-0.44014	1.00000	0.05000
C7	C	-1.89270	-0.16973	-0.55470	1.00000	0.05000
C8	C	-1.82118	-0.19689	-0.58583	1.00000	0.05000
C9	C	-1.79226	-0.25258	-0.56696	1.00000	0.05000
C10	C	-1.83318	-0.28091	-0.51928	1.00000	0.05000
C11	C	-2.03350	-0.11515	-0.49227	1.00000	0.05000

C12	C	-1.99419	-0.08702	-0.54052	1.00000	0.05000
C13	C	-1.92441	-0.11397	-0.57187	1.00000	0.05000
C14	C	-1.88641	-0.08629	-0.62078	1.00000	0.05000
C15	C	-1.81575	-0.11271	-0.65109	1.00000	0.05000
C16	C	-1.77999	-0.16685	-0.63345	1.00000	0.05000
N1	N	-1.70535	-0.19112	-0.66518	1.00000	0.05000
C17	C	-1.62606	-0.16407	-0.68795	1.00000	0.05000
O1	O	-1.57446	-0.19487	-0.72092	1.00000	0.05000
C18	C	-1.54403	-0.10468	-0.60460	1.00000	0.05000
C19	C	-1.59746	-0.10011	-0.66485	1.00000	0.05000
N2	N	-1.53482	-0.06811	-0.70698	1.00000	0.05000
C20	C	-1.44629	-0.05551	-0.67774	1.00000	0.05000
C21	C	-1.44261	-0.09943	-0.62397	1.00000	0.05000
C22	C	-1.57512	-0.04132	-0.75720	1.00000	0.05000
O2	O	-1.65630	-0.05603	-0.76932	1.00000	0.05000
C23	C	-1.52259	0.00058	-0.80285	1.00000	0.05000
N3	N	-1.43931	0.03278	-0.78228	1.00000	0.05000
C24	C	-1.35590	0.00548	-0.80841	1.00000	0.05000
C25	C	-1.38808	-0.05239	-0.83985	1.00000	0.05000
C26	C	-1.50311	0.17784	-0.80356	1.00000	0.05000
C27	C	-1.51295	0.13432	-0.74883	1.00000	0.05000
N4	N	-1.61184	0.11863	-0.74951	1.00000	0.05000
C28	C	-1.66351	0.16078	-0.78807	1.00000	0.05000
C29	C	-1.59531	0.21111	-0.80382	1.00000	0.05000
C30	C	-1.64879	0.08635	-0.70142	1.00000	0.05000
C31	C	-1.75337	0.06861	-0.69930	1.00000	0.05000
O3	O	-1.59696	0.06864	-0.65981	1.00000	0.05000
N5	N	-1.82247	0.08717	-0.74509	1.00000	0.05000
C32	C	-1.90424	0.11249	-0.71581	1.00000	0.05000
C33	C	-1.86788	0.13653	-0.65564	1.00000	0.05000
C34	C	-1.79494	0.08958	-0.63789	1.00000	0.05000
C35	C	-1.43166	0.08804	-0.75320	1.00000	0.05000
O4	O	-1.35336	0.10585	-0.73717	1.00000	0.05000
C36	C	-1.82256	0.05888	-0.80160	1.00000	0.05000
O5	O	-1.75200	0.03399	-0.82147	1.00000	0.05000
C37	C	-1.89701	0.10749	-0.89385	1.00000	0.05000
C38	C	-1.91093	0.06259	-0.84128	1.00000	0.05000
N6	N	-1.92650	0.00415	-0.87115	1.00000	0.05000
C39	C	-1.90559	0.00758	-0.93596	1.00000	0.05000
C40	C	-1.85656	0.06825	-0.94470	1.00000	0.05000
C41	C	-1.95257	-0.04536	-0.83688	1.00000	0.05000
C42	C	-1.98175	-0.10536	-0.86783	1.00000	0.05000
O6	O	-1.96481	-0.03968	-0.78108	1.00000	0.05000
N7	N	-1.93909	-0.12614	-0.92533	1.00000	0.05000
C43	C	-2.01236	-0.14115	-0.96897	1.00000	0.05000
C44	C	-2.09992	-0.14894	-0.93111	1.00000	0.05000

C45	C	-2.08641	-0.10104	-0.88221	1.00000	0.05000
C46	C	-1.72763	-0.17167	-0.84784	1.00000	0.05000
C47	C	-1.77006	-0.14013	-0.90316	1.00000	0.05000
N8	N	-1.70376	-0.15393	-0.95253	1.00000	0.05000
C48	C	-1.64865	-0.20721	-0.93739	1.00000	0.05000
C49	C	-1.67926	-0.22720	-0.87392	1.00000	0.05000
C50	C	-1.69881	-0.11838	-1.00441	1.00000	0.05000
C51	C	-1.62998	-0.13365	-1.05673	1.00000	0.05000
O7	O	-1.74210	-0.06993	-1.00751	1.00000	0.05000
N9	N	-1.62510	-0.19704	-1.07661	1.00000	0.05000
C52	C	-1.53032	-0.22017	-1.07330	1.00000	0.05000
C53	C	-1.47040	-0.16326	-1.07171	1.00000	0.05000
C54	C	-1.53029	-0.11703	-1.03825	1.00000	0.05000
C55	C	-1.86549	-0.16751	-0.92074	1.00000	0.05000
O8	O	-1.87501	-0.22161	-0.93389	1.00000	0.05000
C56	C	-1.64302	-0.30031	-1.17706	1.00000	0.05000
C57	C	-1.62846	-0.35838	-1.19338	1.00000	0.05000
C58	C	-1.66157	-0.40573	-1.15967	1.00000	0.05000
C59	C	-1.71360	-0.39265	-1.10861	1.00000	0.05000
C60	C	-1.72883	-0.33307	-1.09231	1.00000	0.05000
C61	C	-1.69094	-0.28767	-1.12590	1.00000	0.05000
C62	C	-1.75037	-0.43859	-1.07353	1.00000	0.05000
C63	C	-1.80298	-0.42299	-1.02415	1.00000	0.05000
C64	C	-1.74962	-0.60343	-1.07091	1.00000	0.05000
C65	C	-1.78192	-0.32020	-1.04294	1.00000	0.05000
C66	C	-1.64499	-0.46509	-1.17514	1.00000	0.05000
C67	C	-1.68092	-0.51078	-1.13955	1.00000	0.05000
C68	C	-1.73351	-0.49789	-1.08884	1.00000	0.05000
C69	C	-1.66408	-0.57018	-1.15483	1.00000	0.05000
C70	C	-1.69853	-0.61590	-1.12057	1.00000	0.05000
C71	C	-1.81887	-0.36481	-1.00927	1.00000	0.05000
C72	C	-1.76671	-0.54545	-1.05541	1.00000	0.05000
C73	C	-1.59321	-0.48128	-1.22482	1.00000	0.05000
C74	C	-1.57731	-0.53970	-1.23924	1.00000	0.05000
C75	C	-1.61262	-0.58414	-1.20444	1.00000	0.05000
C76	C	-1.69884	-0.22192	-1.10896	1.00000	0.05000
O9	O	-1.76940	-0.19319	-1.12202	1.00000	0.05000
C77	C	-1.48780	-0.03730	-0.85731	1.00000	0.05000
H1	H	-2.04528	-0.27703	-0.37054	1.00000	0.05000
H2	H	-2.09919	-0.17778	-0.40103	1.00000	0.05000
H3	H	-1.92093	-0.32651	-0.42516	1.00000	0.05000
H4	H	-1.73522	-0.27638	-0.58904	1.00000	0.05000
H5	H	-1.80820	-0.32565	-0.50608	1.00000	0.05000
H6	H	-2.08957	-0.09247	-0.46795	1.00000	0.05000
H7	H	-2.01969	-0.04231	-0.55337	1.00000	0.05000
H8	H	-1.91203	-0.04280	-0.63713	1.00000	0.05000

H9	H	-1.78944	-0.08957	-0.69148	1.00000	0.05000
H10	H	-1.70587	-0.23639	-0.67316	1.00000	0.05000
H11	H	-1.56211	-0.06606	-0.57527	1.00000	0.05000
H12	H	-1.55777	-0.14652	-0.57921	1.00000	0.05000
H13	H	-1.65767	-0.07104	-0.65765	1.00000	0.05000
H14	H	-1.44643	-0.00844	-0.66172	1.00000	0.05000
H15	H	-1.38798	-0.06302	-0.70864	1.00000	0.05000
H16	H	-1.39733	-0.08315	-0.58715	1.00000	0.05000
H17	H	-1.41740	-0.14405	-0.63905	1.00000	0.05000
H18	H	-1.57307	0.03373	-0.81945	1.00000	0.05000
H19	H	-1.32747	0.03765	-0.84181	1.00000	0.05000
H20	H	-1.30288	-0.00215	-0.77334	1.00000	0.05000
H21	H	-1.34478	-0.06451	-0.87916	1.00000	0.05000
H22	H	-1.38713	-0.09091	-0.80822	1.00000	0.05000
H23	H	-1.44375	0.20862	-0.79881	1.00000	0.05000
H24	H	-1.49433	0.15241	-0.84639	1.00000	0.05000
H25	H	-1.50188	0.15992	-0.70632	1.00000	0.05000
H26	H	-1.72448	0.17922	-0.76480	1.00000	0.05000
H27	H	-1.68358	0.13697	-0.82983	1.00000	0.05000
H28	H	-1.59600	0.24601	-0.76816	1.00000	0.05000
H29	H	-1.61117	0.23301	-0.84741	1.00000	0.05000
H30	H	-1.75305	0.01881	-0.69941	1.00000	0.05000
H31	H	-1.95496	0.07593	-0.70878	1.00000	0.05000
H32	H	-1.93606	0.14767	-0.74418	1.00000	0.05000
H33	H	-1.92226	0.14173	-0.62115	1.00000	0.05000
H34	H	-1.83465	0.18072	-0.66257	1.00000	0.05000
H35	H	-1.82856	0.05097	-0.61535	1.00000	0.05000
H36	H	-1.74398	0.10812	-0.60569	1.00000	0.05000
H37	H	-1.96446	0.12563	-0.90863	1.00000	0.05000
H38	H	-1.85186	0.14582	-0.88225	1.00000	0.05000
H39	H	-1.97234	0.07389	-0.81431	1.00000	0.05000
H40	H	-1.97126	0.00574	-0.96102	1.00000	0.05000
H41	H	-1.86214	-0.02994	-0.95017	1.00000	0.05000
H42	H	-1.86769	0.08718	-0.99033	1.00000	0.05000
H43	H	-1.78126	0.06296	-0.93815	1.00000	0.05000
H44	H	-1.97027	-0.14157	-0.83404	1.00000	0.05000
H45	H	-1.99655	-0.18095	-0.99695	1.00000	0.05000
H46	H	-2.01927	-0.10190	-0.99944	1.00000	0.05000
H47	H	-2.10192	-0.19409	-0.91017	1.00000	0.05000
H48	H	-2.16368	-0.14238	-0.95750	1.00000	0.05000
H49	H	-2.12961	-0.10901	-0.84176	1.00000	0.05000
H50	H	-2.10465	-0.05611	-0.90030	1.00000	0.05000
H51	H	-1.67362	-0.14270	-0.82845	1.00000	0.05000
H52	H	-1.77742	-0.18274	-0.81178	1.00000	0.05000
H53	H	-1.77542	-0.09127	-0.89598	1.00000	0.05000
H54	H	-1.57495	-0.19517	-0.93778	1.00000	0.05000

H55	H	-1.66106	-0.24317	-0.97057	1.00000	0.05000
H56	H	-1.62024	-0.24173	-0.84589	1.00000	0.05000
H57	H	-1.72748	-0.26548	-0.87694	1.00000	0.05000
H58	H	-1.65076	-0.10693	-1.09692	1.00000	0.05000
H59	H	-1.51228	-0.24913	-1.11190	1.00000	0.05000
H60	H	-1.52321	-0.24668	-1.03131	1.00000	0.05000
H61	H	-1.45744	-0.14722	-1.11841	1.00000	0.05000
H62	H	-1.40307	-0.17087	-1.04972	1.00000	0.05000
H63	H	-1.51295	-0.07040	-1.05143	1.00000	0.05000
H64	H	-1.51897	-0.12012	-0.98891	1.00000	0.05000
H65	H	-1.61488	-0.26388	-1.20509	1.00000	0.05000
H66	H	-1.58820	-0.36420	-1.23483	1.00000	0.05000
H67	H	-1.83572	-0.45572	-0.99412	1.00000	0.05000
H68	H	-1.77728	-0.64021	-1.04326	1.00000	0.05000
H69	H	-1.79795	-0.27393	-1.02902	1.00000	0.05000
H70	H	-1.68598	-0.66319	-1.13212	1.00000	0.05000
H71	H	-1.86184	-0.35335	-0.96998	1.00000	0.05000
H72	H	-1.80831	-0.53985	-1.01452	1.00000	0.05000
H73	H	-1.56160	-0.44876	-1.25561	1.00000	0.05000
H74	H	-1.53576	-0.55105	-1.27912	1.00000	0.05000
H75	H	-1.59859	-0.63096	-1.21714	1.00000	0.05000
H76	H	-1.48784	-0.00929	-0.89886	1.00000	0.05000
H77	H	-1.52958	-0.07770	-0.86687	1.00000	0.05000

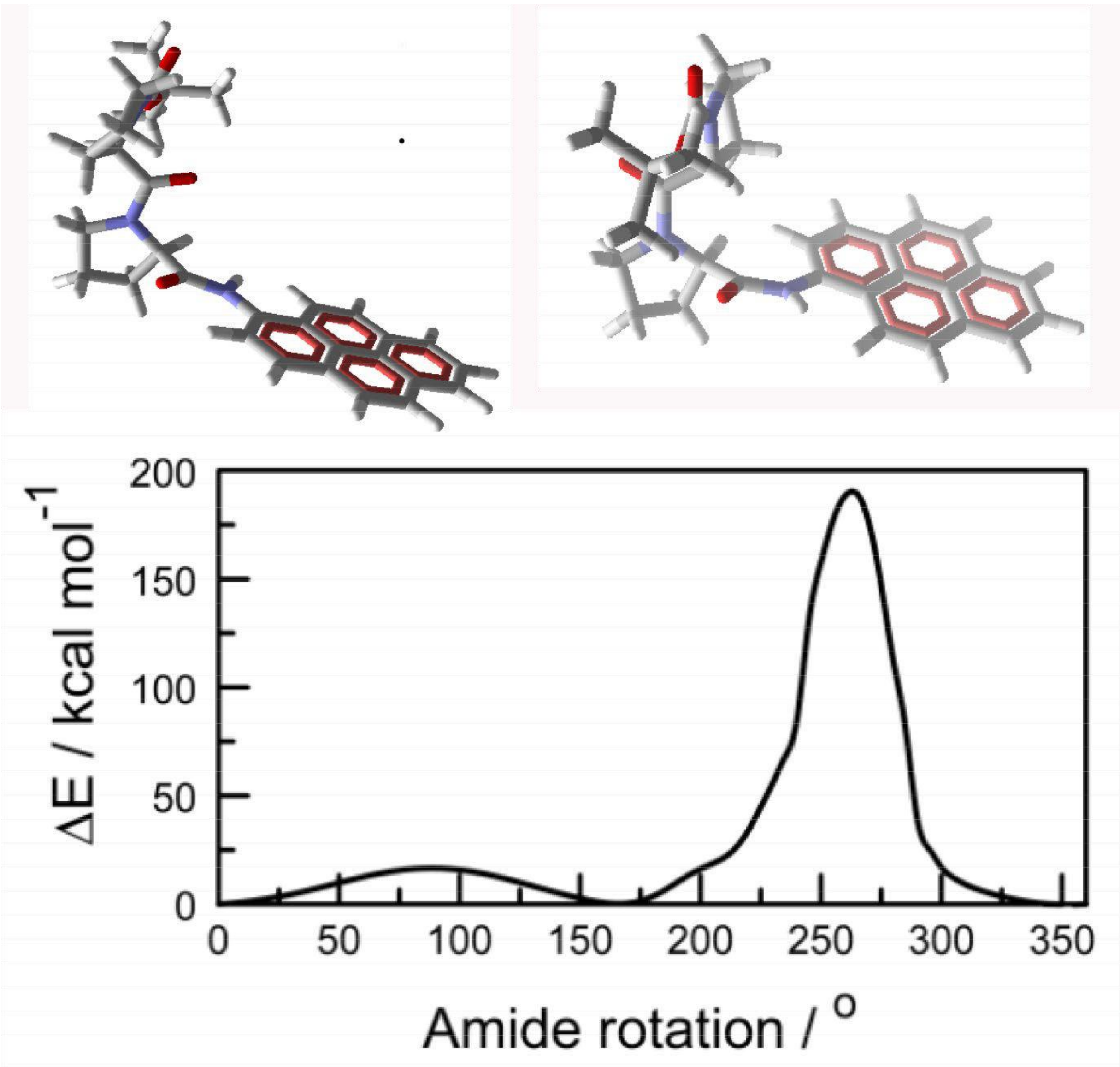

Figure S53. Result of the rotational energy barrier calculation made for **PY-P$_2$** in a reservoir of $CHCl_3$ molecules. The structures shown above the main panel are for the 0° and 18O^o rotations. Bond rotation was made systematically from 0° in both directions. Each structure along the trajectory was minimised in terms of energy.

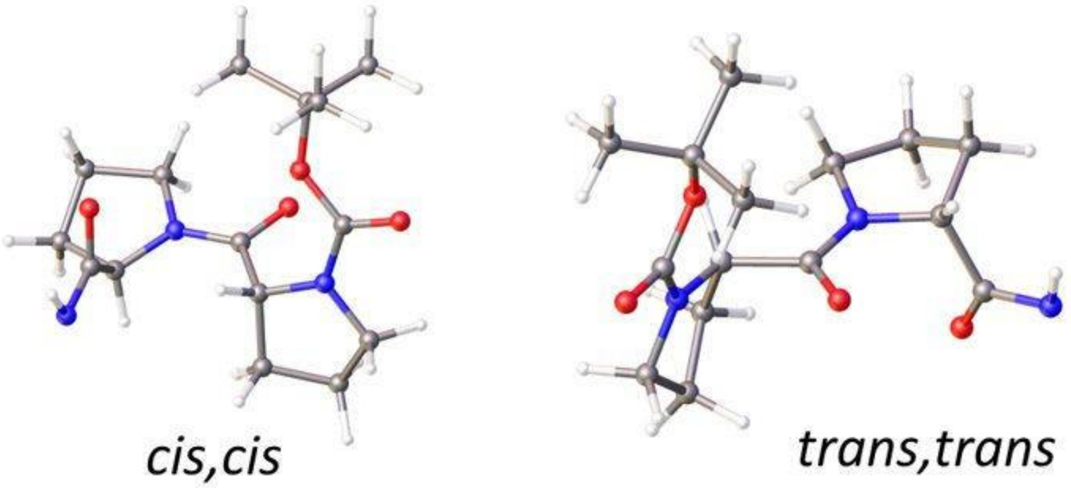

Figure S54. Cropped structures highlighting the geometry of the *bis*-proline chain determined by DFT calculations for the two isomers identified of **PY-P$_2$**.

Structures for the *cis*- and *trans*-isomers of **PY-P$_8$-PER** were calculated as for **PY-P$_2$** and fully optimised. Again, the solvent was $CHCl_3$ treated by the polarisation continuum model with the MT correction. In making these calculations, the terminal amide was constrained to be either *cis* or *trans*, with no other structural constraints. Cartesian coordinates for the two final structures are given below. Our nomenclature assigns *cis* or *trans* geometries only to the amide bond attached directly to pyrene and ignores all other possible isomers. More detailed examination of the final structures allows assignment of the remaining amide geometries associated with the proline units. All structures were interrogated for internal hydrogen bonds by importing the energy-minimised structure into Olex-2 but no realistic examples were found. Initial searches for stable conformers used the Monte Carlo random searching algorithm[S24] and the MMFF94 force field implemented in Spartan. This provides a cost effective process that samples the entire conformational space. The conformers obtained in this manner were used as input structures for subsequent DFT calculations using the GAMESS platform. All calculations were performed at the B3LYP level of theory using the standard 6-31+G(d,p) basis set. The use of diffuse and polarization functions are intended to ensure converged geometries in a timely manner. Full geometry optimisations in the gas phase was followed by frequency calculations to characterise the stationary points as true minima. No imaginary frequencies were observed. The gas-phase structures were further optimised in liquid phase using the PCM continuum solvation model and with chloroform as the solvent.

An abitary energy cut-off of 10 kcal mol^{-1} was applied to the conformers, meaning that many possible structures were deemed to be too unstable to include in subsequent EET calculations. The primary distinction between classes of conformer was made according to the geometry of the amide at the pyrene terminal. Rather than rely on the relative energies obtained by calculation, the fractional contributions of *cis* and *trans* isomers were established from the NMR studies made in $CHCl_3$ and CH_3OH. The secondary contributors, namely T2, C2 and C3, were assigned fractional contributions on the basis of their total energy relative to T1 or C1.

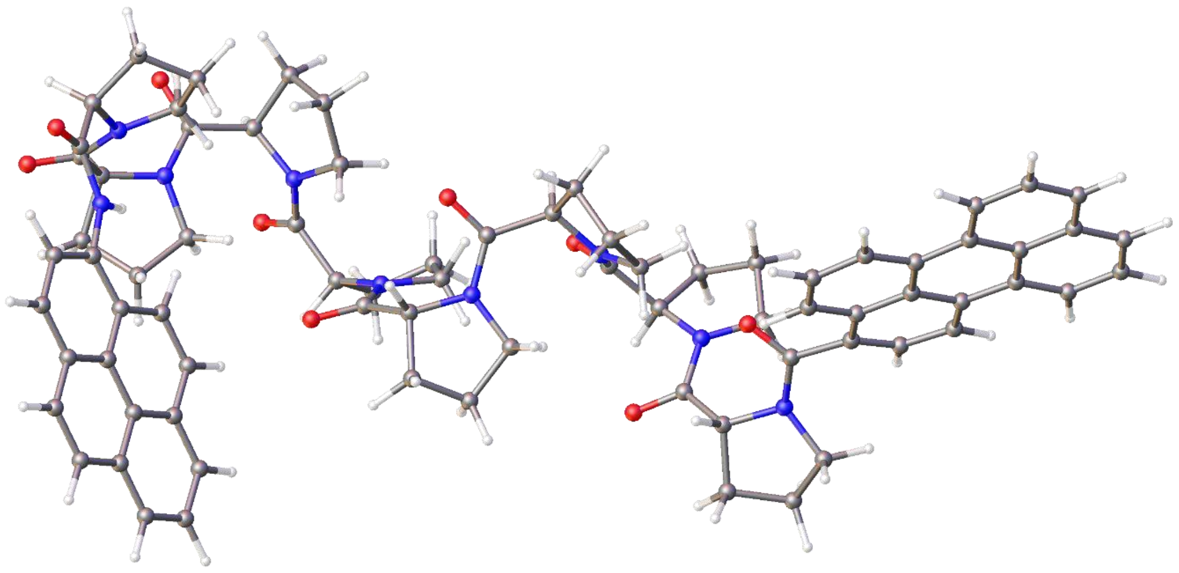

Figure S55. Representation of the 3D structure computed for the *all-trans* configuration of **PY-P$_8$-PER** in a reservoir of $CHCl_3$ molecules. This structure is referred to T1.

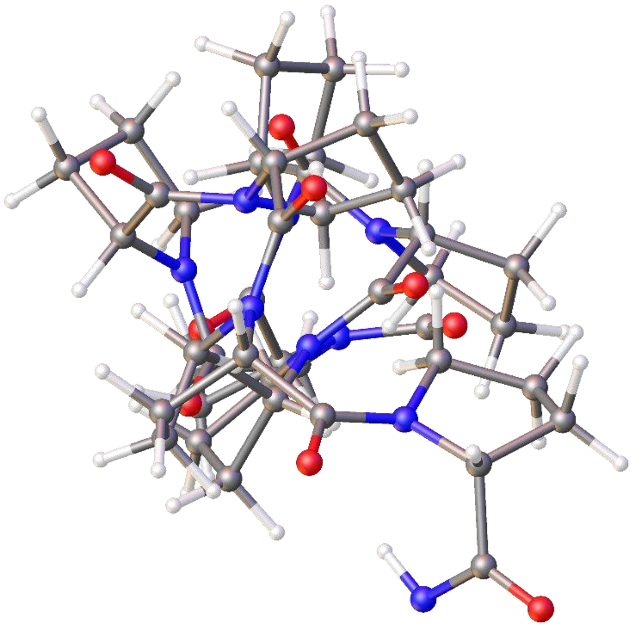

Figure S56. Illustration of the packing of the oligo-proline chain determined for the all-*trans* configuration of **PY-P$_8$-PER**. The polycyclic terminals have been deleted without affecting the geometry of the chain.

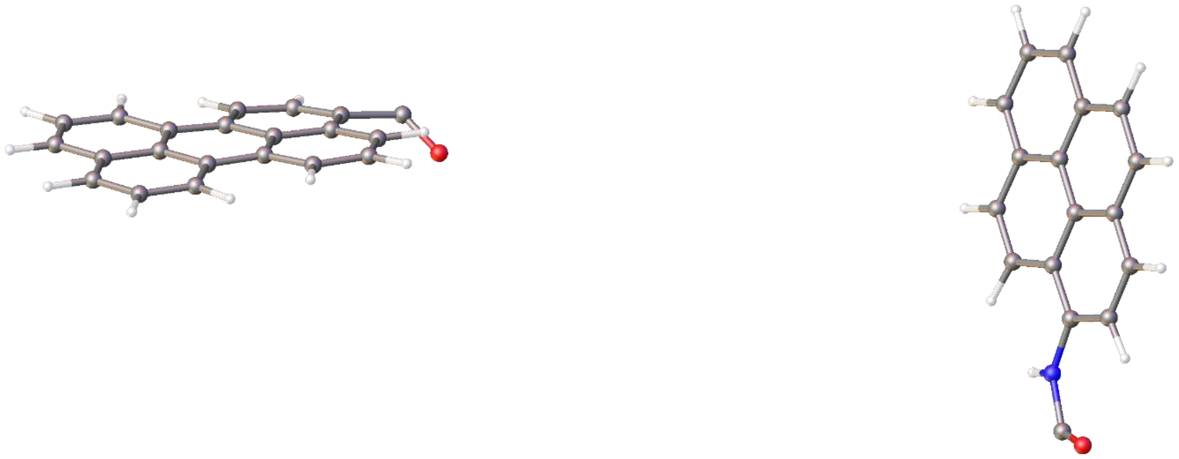

Figure S57. Illustration of the mutual orientation of the polycyclic terminals as found for the all-*trans* configuration of **PY-P$_8$-PER**. The oligo-proline chain has been deleted without affecting the orientation of the terminals.

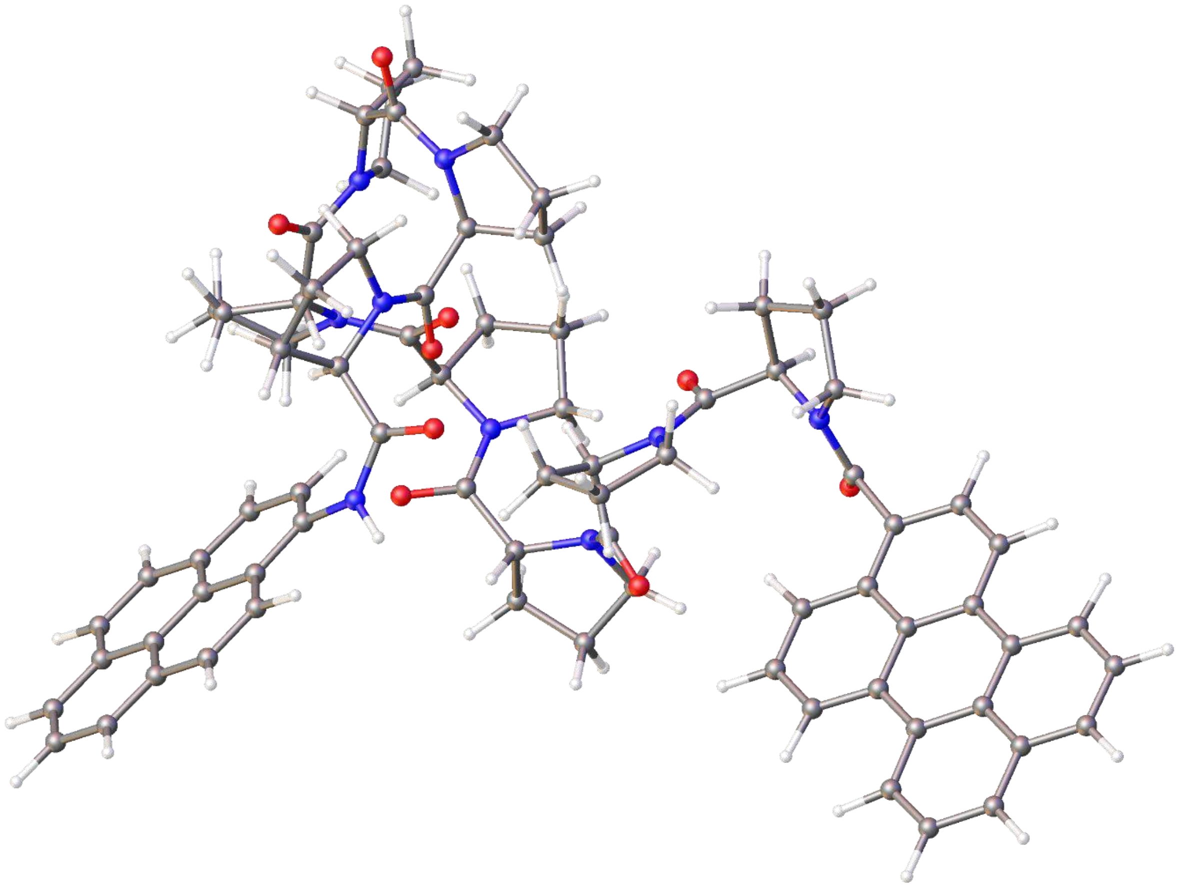

Figure S58. Representation of the 3D structure computed for the lowest-energy structure having a terminal *cis*-amide configuration of **PY-P$_8$-PER** in a reservoir of $CHCl_3$ molecules. This structure is referred to as C1. *Cis*-amides are located at 1, 3 and 8 sites.

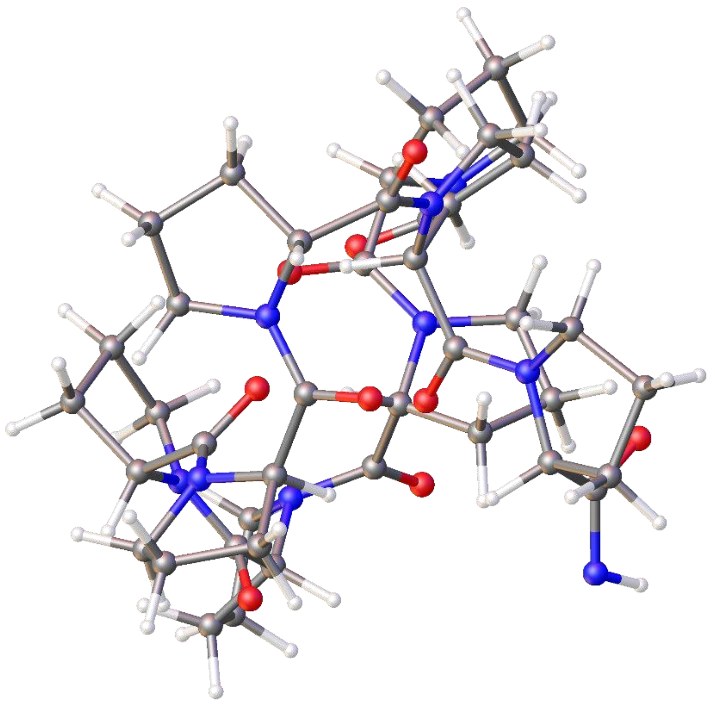

Figure S59. Illustration of the packing of the oligo-proline chain determined for the lowest-energy *cis* configuration of **PY-P$_8$-PER**, known as C1. The polycyclic terminals have been deleted without affecting the geometry of the chain.

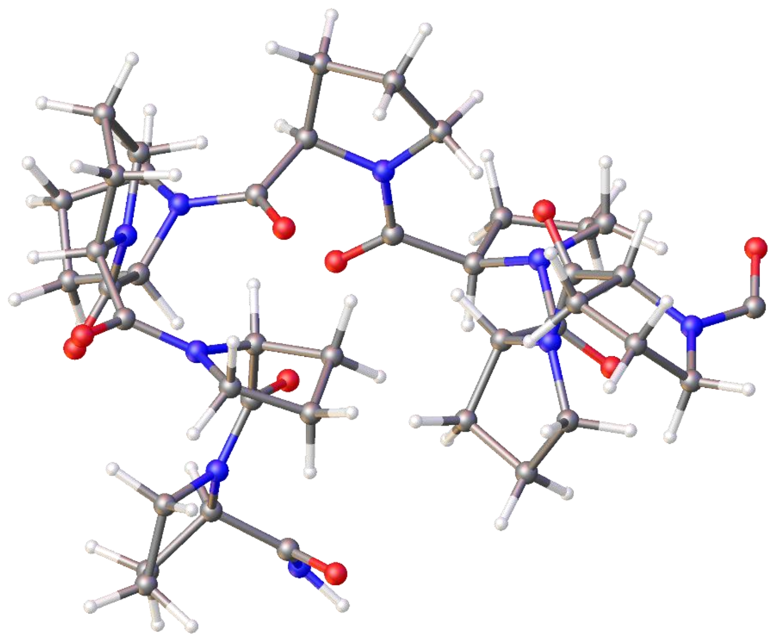

Figure S60. Further illustration of the packing of the oligo-proline chain determined for the lowest-energy *cis* configuration of **PY-P$_8$-PER**, known as C1.

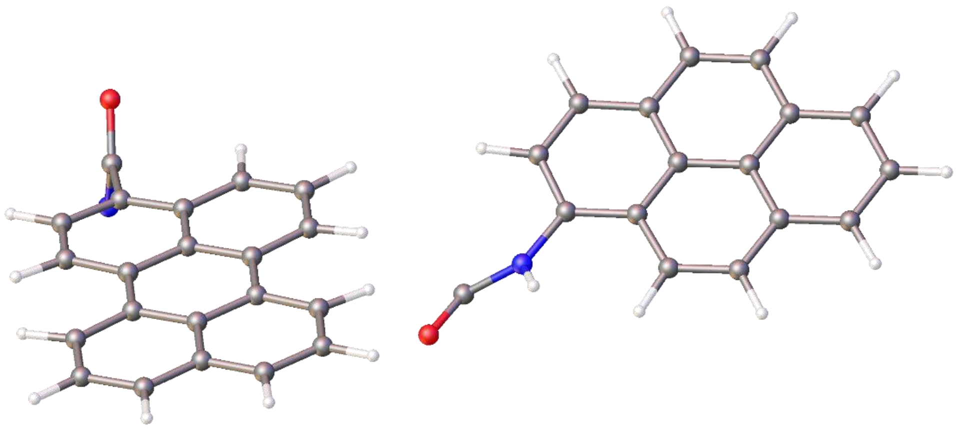

Figure S61. Illustration of the mutual orientation of the polycyclic terminals as found for the lowest-energy *cis* configuration of **PY-P$_8$-PER**. The oligo-proline chain has been deleted without affecting the orientation of the terminals.

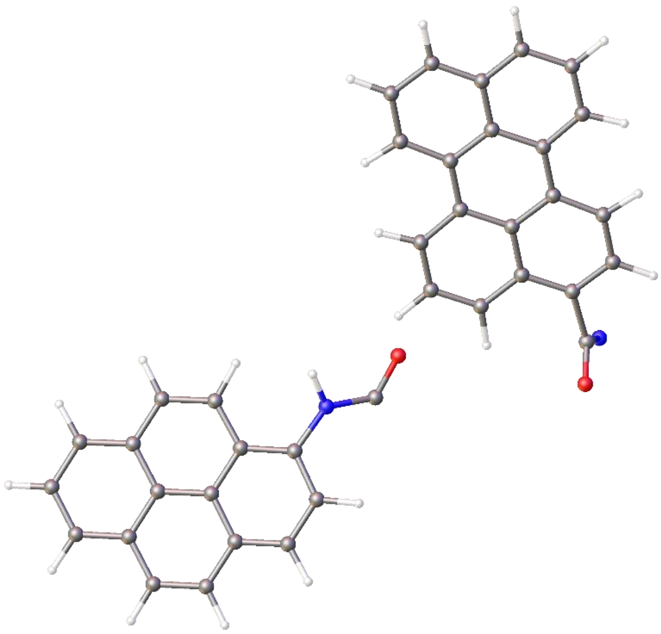

Figure S62. Further illustration of the mutual orientation of the polycyclic terminals as found for the lowest-energy *cis* configuration of **PY-P$_8$-PER**. The oligo-proline chain has been deleted without affecting the orientation of the terminals.

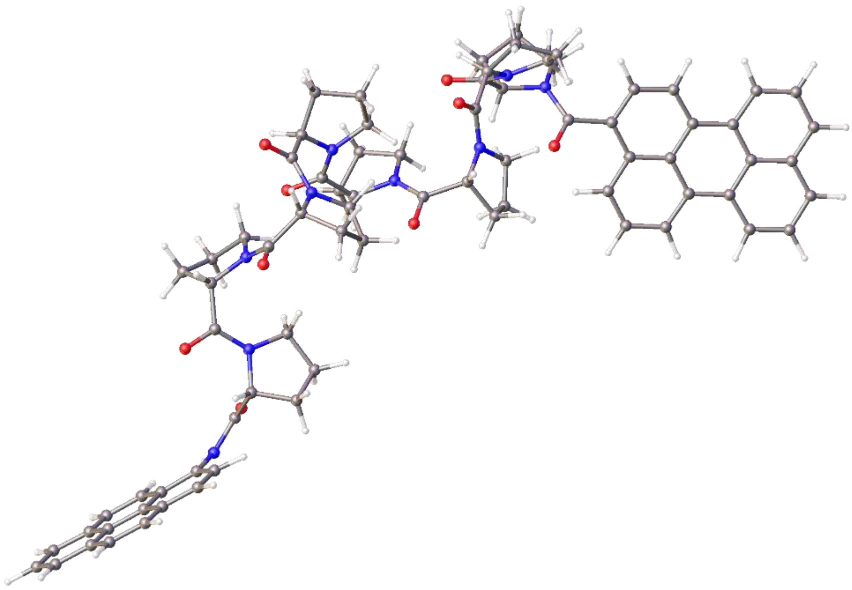

Figure S63. Illustration of the 3D structure computed for C2 in a reservoir of $CHCl_3$ molecules. This arrangement has a single *cis*-amide at the pyrene terminal. The fold angle is 35.0° and the twist angle is 85.3°. The plane-plane centre distance is 25.6Å.

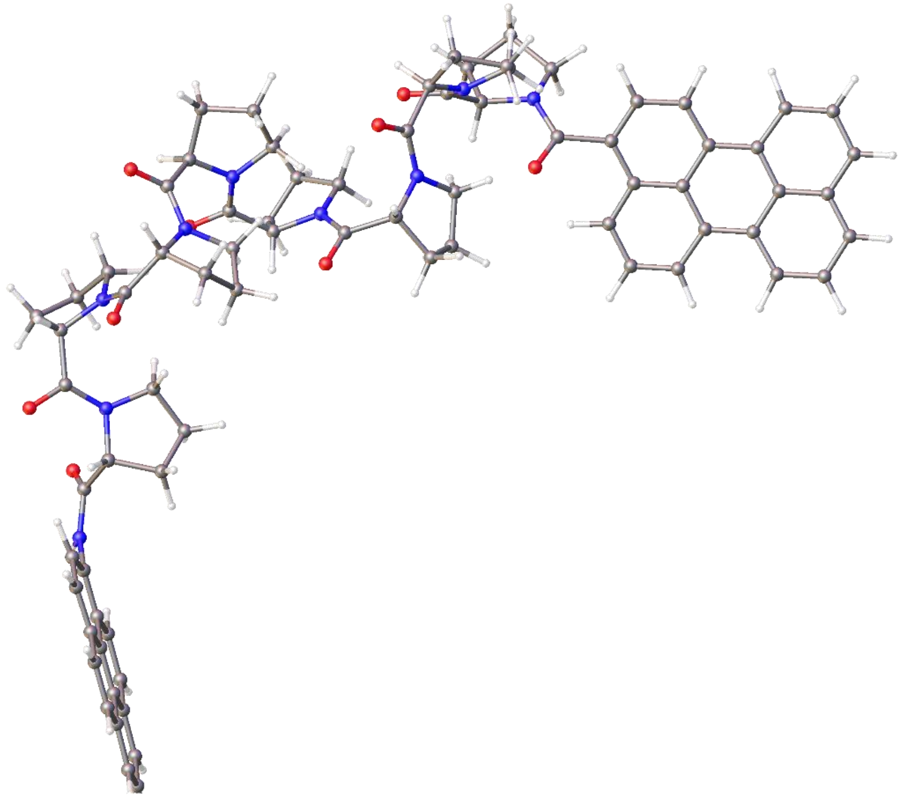

Figure S64. Illustration of the 3D structure computed for C3 in a reservoir of $CHCl_3$ molecules. This arrangement has a single *cis*-amide at the pyrene terminal. The fold angle is 94.6° and the twist angle is 132.8°. The plane-plane centre distance is 23.1Å.

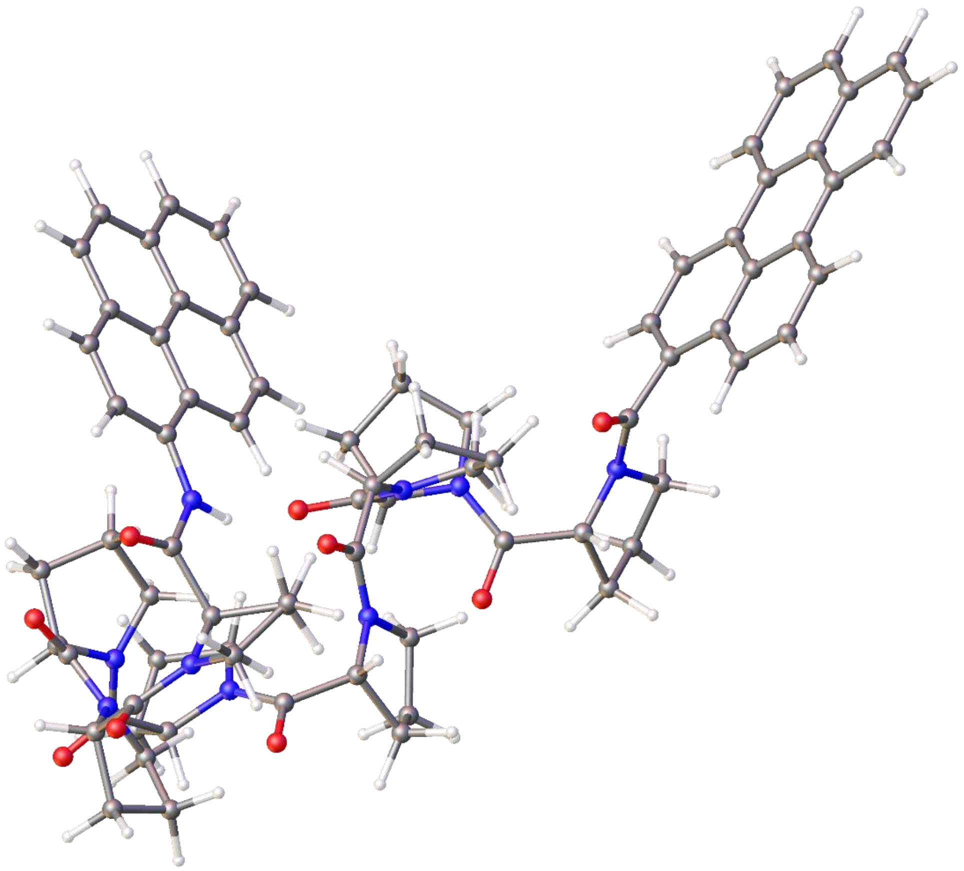

Figure S65. Illustration of the 3D structure computed for T2 in a reservoir of $CHCl_3$ molecules. This arrangement has a *trans*-amide at the pyrene terminal and a *cis*-amide at the 2-site. All other amides adopt the *trans*-geometry. The fold angle is 77.4^{o} and the twist angle is 63.1^{o}. The plane-plane centre distance is 14.68Å.

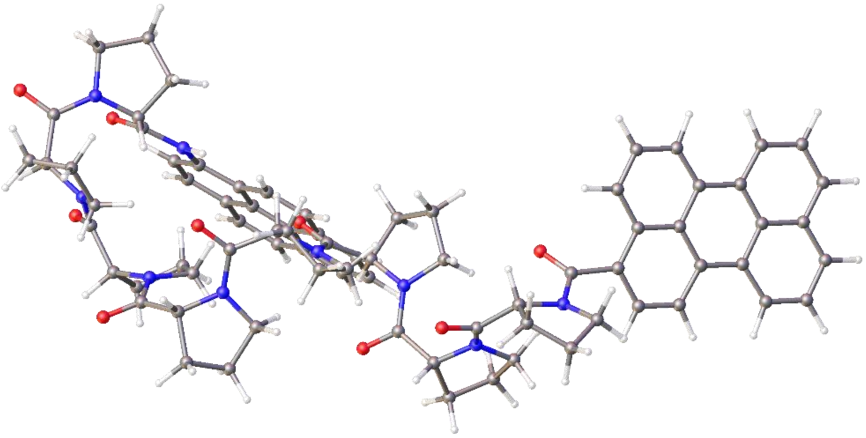

Figure S66. Further illustration of the 3D structure computed for T2 in a reservoir of $CHCl_3$ molecules. This arrangement is intended to highlight the *cis*-amide at the 2-site.

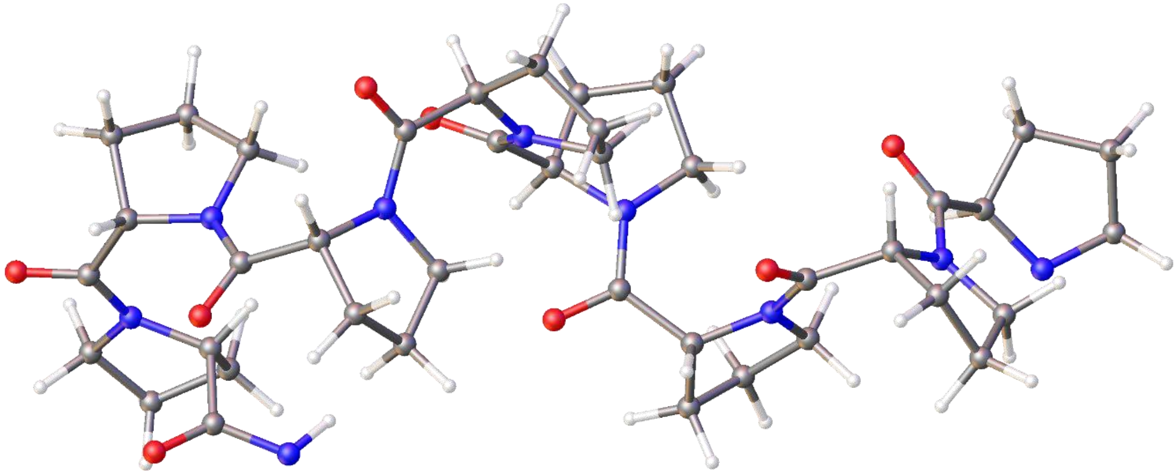

Figure S67. Illustration of the 3D structure computed for T2 in a reservoir of $CHCl_3$ molecules, emphasising the geometry of the ologo-proline chain. The polycyclic terminals have been deleted without affecting the structure of the chain. Note, this arrangement has a *cis*-amide at the 2-site. All other amides adopt the *trans*-geometry.

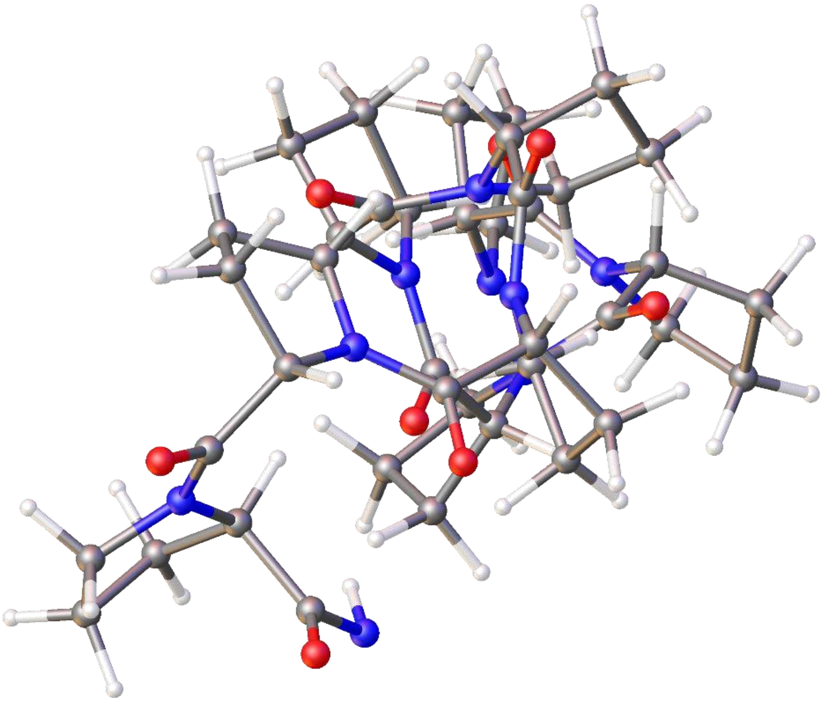

Figure S68. Further illustration of the 3D structure computed for T2 in a reservoir of $CHCl_3$ molecules, emphasising the geometry of the ologo-proline chain. The polycyclic terminals have been deleted without affecting the structure of the chain. Note, this arrangement has a *cis*-amide at the 2-site. All other amides adopt the *trans*-geometry.

S7. Time-resolved fluorescence studies with the dyad

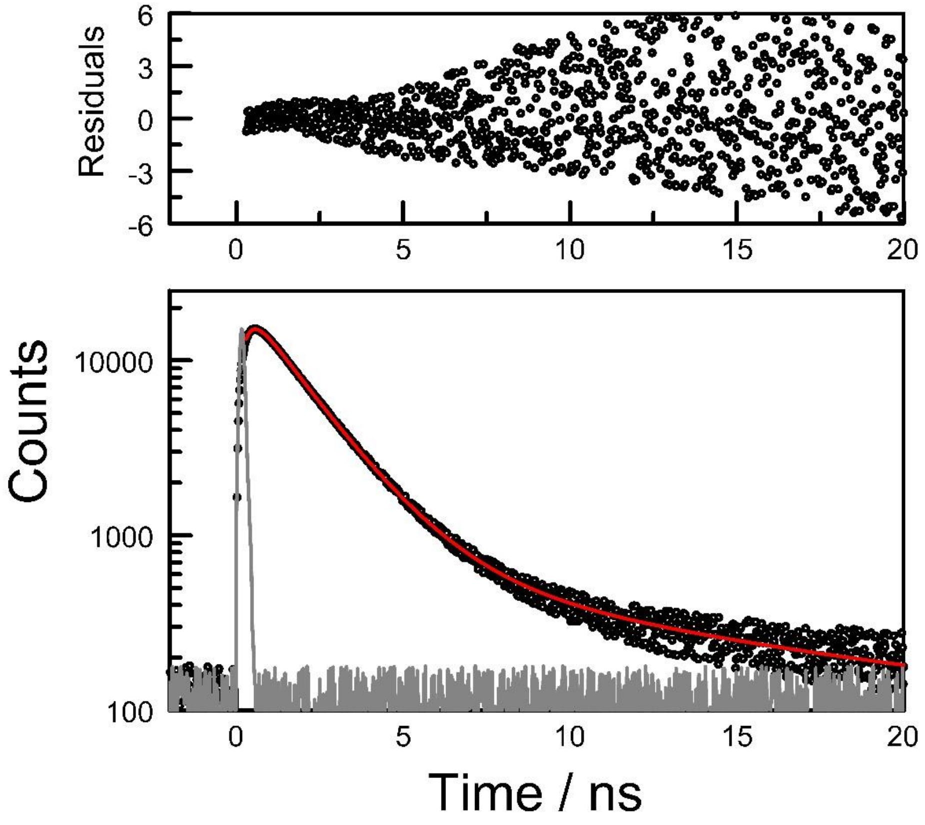

Figure S69. Example of a fluorescence decay curve recorded for pyrene emission from **PY-P$_8$-PER** in methanol with excitation at 330 nm. Detection was made at 385 nm. The instrument response function is shown as a grey curve while the red line running through the data points (open circles) corresponds to a non-linear least-squares fit to a dual-exponential process. The CPC has a maximum of *ca*. 15,000. The best fit gives lifetimes of 1.62 ns (93%) and 8.9 ns (7%). The upper panel shows the weighted reduced residuals, which indicates that the overall fit is poor, especially at longer times. The fit was made from just short of the CPC to 20 ns.

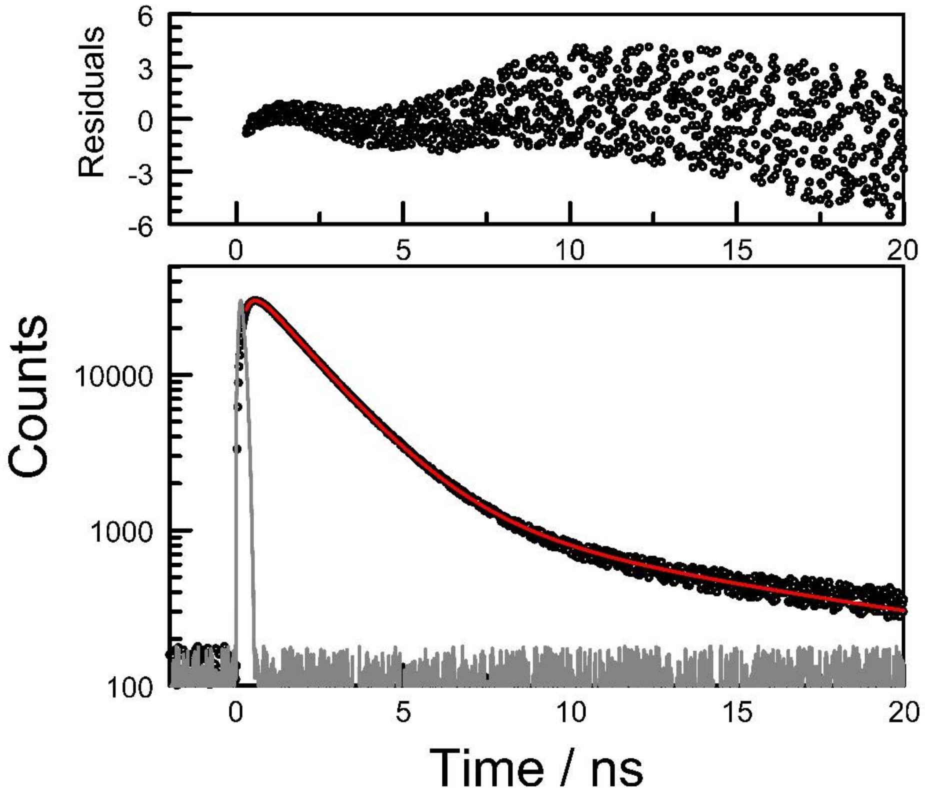

Figure S70. Example of a fluorescence decay curve recorded for pyrene emission from **PY-P_8-PER** in methanol with excitation at 330 nm. Detection was made at 390 nm. The instrument response function is shown as a grey curve while the red line running through the data points (open circles) corresponds to a non-linear least-squares fit to a dual-exponential process. The CPC has a maximum of *ca*. 30,000. The best fit gives lifetimes of 1.68 ns (94%) and 9.8 ns (6%). The upper panel shows the weighted reduced residuals, which indicates that the overall fit is poor, especially at longer times. The fit was made from just short of the CPC to 20 ns.

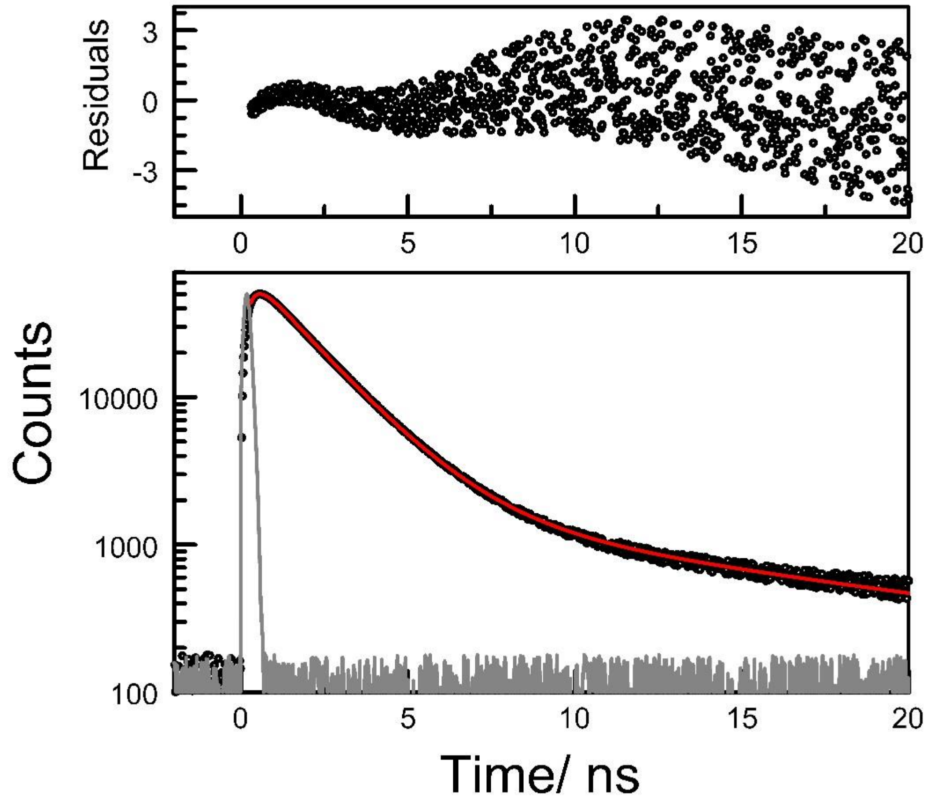

Figure S71. Example of a fluorescence decay curve recorded for pyrene emission from **PY-P_8-PER** in methanol with excitation at 330 nm. Detection was made at 380 nm. The instrument response function is shown as a grey curve while the red line running through the data points (open circles) corresponds to a non-linear least-squares fit to a dual-exponential process. The CPC has a maximum of *ca*. 50,000. The best fit gives lifetimes of 1.70 ns (96%) and 11.7 ns (4%). The upper panel shows the weighted reduced residuals, which indicates that the overall fit is poor, especially at longer times. The fit was made from just short of the CPC to 20 ns.

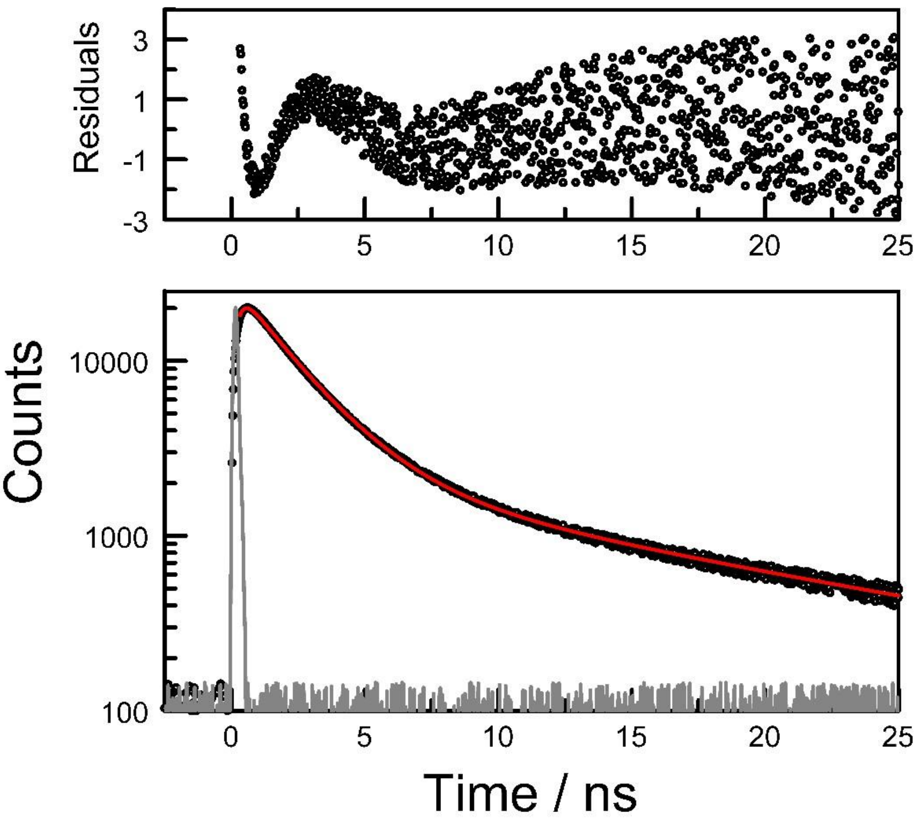

Figure S72. Example of a fluorescence decay curve recorded for pyrene emission from **PY-P$_8$-PER** in chloroform with excitation at 330 nm. Detection was made at 370 nm. The instrument response function is shown as a grey curve while the red line running through the data points (open circles) corresponds to a non-linear least-squares fit to a dual-exponential process. The CPC has a maximum of *ca*. 20,000. The best fit gives lifetimes of 2.03 ns (82%) and 13.4 (18%) ns. The upper panel shows the weighted reduced residuals, which indicates that the overall fit is very poor, especially at short times. The fit was made from just short of the CPC to 25 ns.

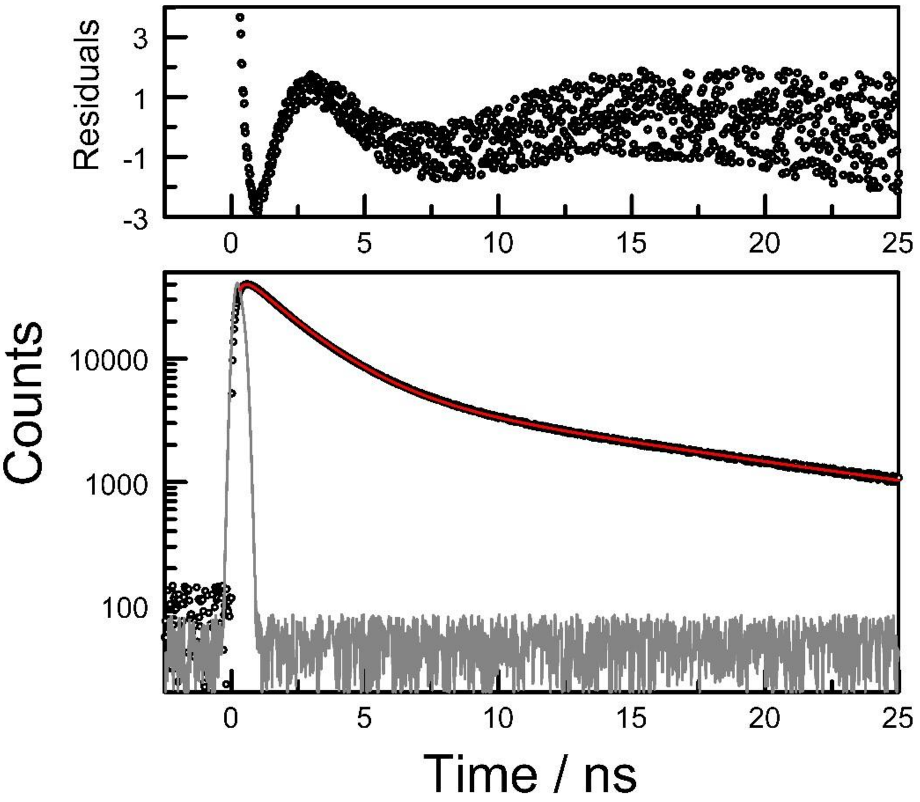

Figure S73. Example of a fluorescence decay curve recorded for pyrene emission from **PY-P$_8$-PER** in chloroform with excitation at 330 nm. Detection was made at 370 nm. The instrument response function is shown as a grey curve while the red line running through the data points (open circles) corresponds to a non-linear least-squares fit to a dual-exponential process. The CPC has a maximum of *ca*. 40,000. The best fit gives lifetimes of 2.01 ns (79%) and 11.5 ns (21%). The upper panel shows the weighted reduced residuals, which indicates that the overall fit is very poor, especially at short times. The fit was made from just short of the CPC to 25 ns.

S8. Simulation of TCSPC decays using a distributive model

Tradionally, time-correlated, single photon counting data have been analysed in terms of a series of exponential components, each characterised by a fractional contribution and a corresponding lifetime.[S25] This situation is conveyed by Equation 6. The quality of the fit increases with increasing number of exponentials but the interpretation becomes evermore cloudy. This is what we find for pyrene emission from **PY-P_8-PER**, where neither a single- or dual-exponential kinetics gives a satisfactory account of the decay process. Alternative analytical procedures are available. Thus, Ware and colleagues[S26] were among the first to question the validity of the multi-exponential fitting routine when dealing with complex decays. This work resulted in the introduction of a model whereby a Gaussian distribution of lifetimes was applied to the analysis of fluorophores attached to a surface. The advantage of using a fixed distribution is that the fractional amplitude is fixed by the shape of the distribution. A similar approach can be applied[S27] to analyse emission from a distribution of emitters undergoing FRET but only if the orientation factor is fixed (usually to 0.67). A related analytical protocol uses the maximum entropy method (MEM),[S28] which returns a distribution of conformers but their identity is obscured.[S25] The difficulty encountered when applying these methods to pyrene fluorescence from **PY-P_8-PER** is that a limited number of conformers is involved but these are non-interconverting and subject to disparate separation distances and mutual orientations. It does not seem appropriate to assume $\kappa^2 = 0.67$ for this set of conformers where the connecting spacer seems to be stiff. A well-known protocol for handling non-exponential decays involves analysis in the form of a stretched exponential (or Kohlrausch) function or in terms of a compressed hyperbola (or Becquerol) function. These latter methods allow fitting of non-linear data but offer little in the way of interpretation.

Our solution to this challenge is to put high (perhaps unduly so) confidence in the computed structures and to calculate fluorescence lifetimes for each structure. This approach is aided by the realisation that P_{EET} exceeds 80% in all cases so that the lifetime is effectively determined by EET. It is asking too much for the computational studies to accurately predict the effect of solvent on the distribution of conformers but the global *cis*/*trans* ratio is available from NMR studies. The computational studies indicate that we need to consider two *trans*-conformers (all-*trans* (T1)) and TCTTTTTTT (T2) and three *cis*-conformers. The lowest-energy *cis*-conformer (C1) has three *cis*-amides (CTCTTTCTT) in the chain. The complementary *cis*-

species have a terminal *cis*-amide and an all-*trans* chain. The computed total energies are used to establish the composition of the *cis* and *trans* families but not their relative significance, which is set by NMR. This gives an initial distribution of five lifetimes (τ_N) and their fractional contributions (A_N), as compiled in Table S6. The projected lifetimes for C2 ($\tau \approx$ 11 ns) and C3 ($\tau \approx$ 13 ns), considered in terms of their relatively low contributions, are too similar for meaningful distinction given their low amplitudes and an averaged lifetime (C2/3) was used in conjunction with their combined amplitudes.

Table S6. Conformer properties used as primary input for the distributive model used to analyse the time-resolved fluorescence decays.

Parameter	T1	T2	C1	C2/3$^{(d)}$
Composition	TTTTTTTTT	TCTTTTTTT	CTCTTTCTT	CTTTTTTTT
r / Å	19.1	14.7	14.6	24.3
κ^2	1.15	0.33	0.05	NA
τ / ns $^{(a)}$	1.7	0.83	2.6	11.4
A / % $^{(b)}$	85	5	8	2
A / % $^{(c)}$	52	8	33	7

(a) Inverse of the rate constant for intramolecular EET calculated using the structural information provided by the DFT calculations. (b) Methanol. (c) Chloroform. (d) Averaged values for C2 and C3.

The tabulated lifetimes (τ) and fractional contributions (A) of the four emitters were convoluted with an experimental IRF having the requisite CPC to match the measured trace. The simulated decays were not artifically doctored with Poisson scatter to mimic the expected noise inherent to all time-correlated, single photon counting experiments. This situation tends to amplify the difference between simulated and experimental records at long delay times, leading to higher than normal κ^2 values. Iteration was continued until the best global fit was obtained but this stepwise optimisation was carried out through many small steps, with each lifetime being varied in sequence. The reduced weighted residual and their

autocorrelation were studied at each step to aid recognition of which lifetime required attention. The protocol used a small Monte Carlo algorithm. The sum of the A values was constrained to unity in all cases.

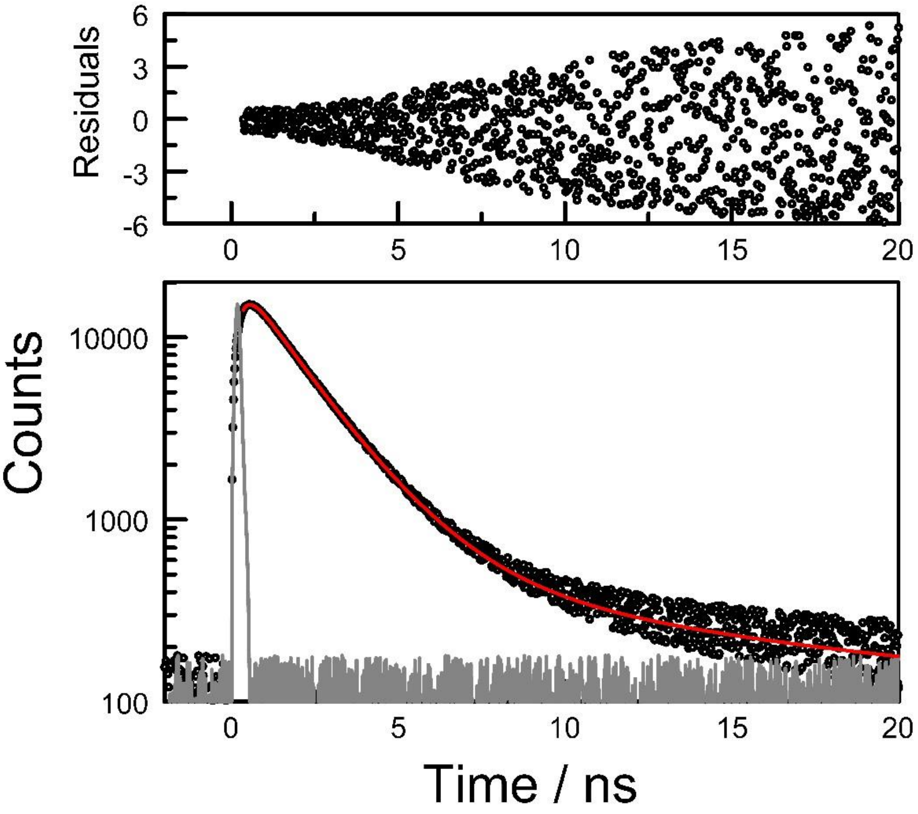

Figure S74. Example of a fluorescence decay curve recorded for pyrene emission from **PY-P_8-PER** in methanol with excitation at 330 nm. Detection was made at 370 nm. The instrument response function is shown as a grey curve while the red line running through the data points (open circles) corresponds to a non-linear least-squares fit to a distributive model with four components. The CPC has a maximum of *ca*. 15,000. The upper panel shows the weighted reduced residuals, which indicates that the overall fit is excellent. The fit was made from just short of the CPC to 20 ns.

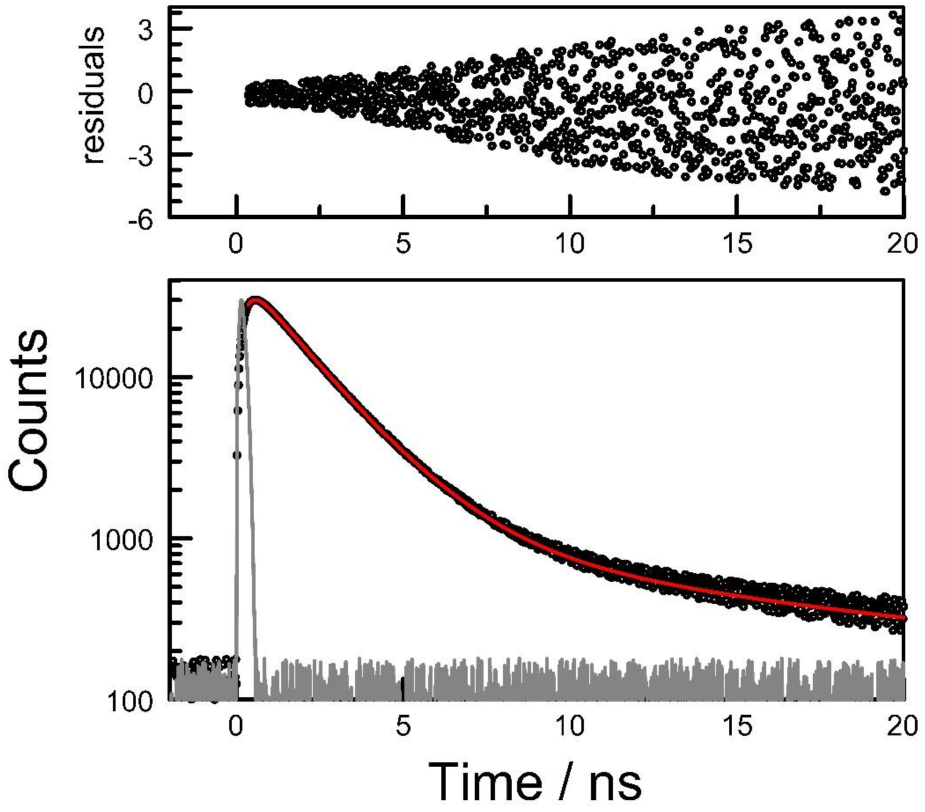

Figure S75. Example of a fluorescence decay curve recorded for pyrene emission from **PY-P_8-PER** in methanol with excitation at 330 nm. Detection was made at 370 nm. The instrument response function is shown as a grey curve while the red line running through the data points (open circles) corresponds to a non-linear least-squares fit to a distributive model with four components. The CPC has a maximum of *ca*. 30,000. The upper panel shows the weighted reduced residuals, which indicates that the overall fit is excellent. The fit was made from just short of the CPC to 20 ns.

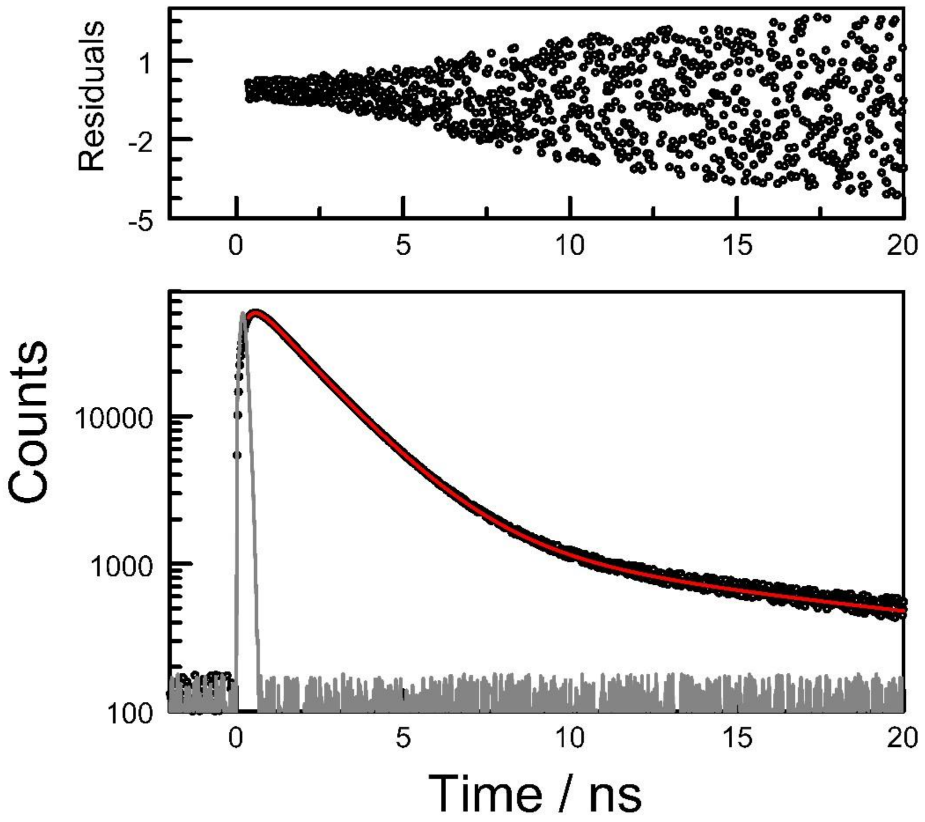

Figure S76. Example of a fluorescence decay curve recorded for pyrene emission from **PY-P_8-PER** in methanol with excitation at 330 nm. Detection was made at 370 nm. The instrument response function is shown as a grey curve while the red line running through the data points (open circles) corresponds to a non-linear least-squares fit to a distributive model with four components. The CPC has a maximum of *ca*. 50,000. The upper panel shows the weighted reduced residuals, which indicates that the overall fit is excellent. The fit was made from just short of the CPC to 20 ns.

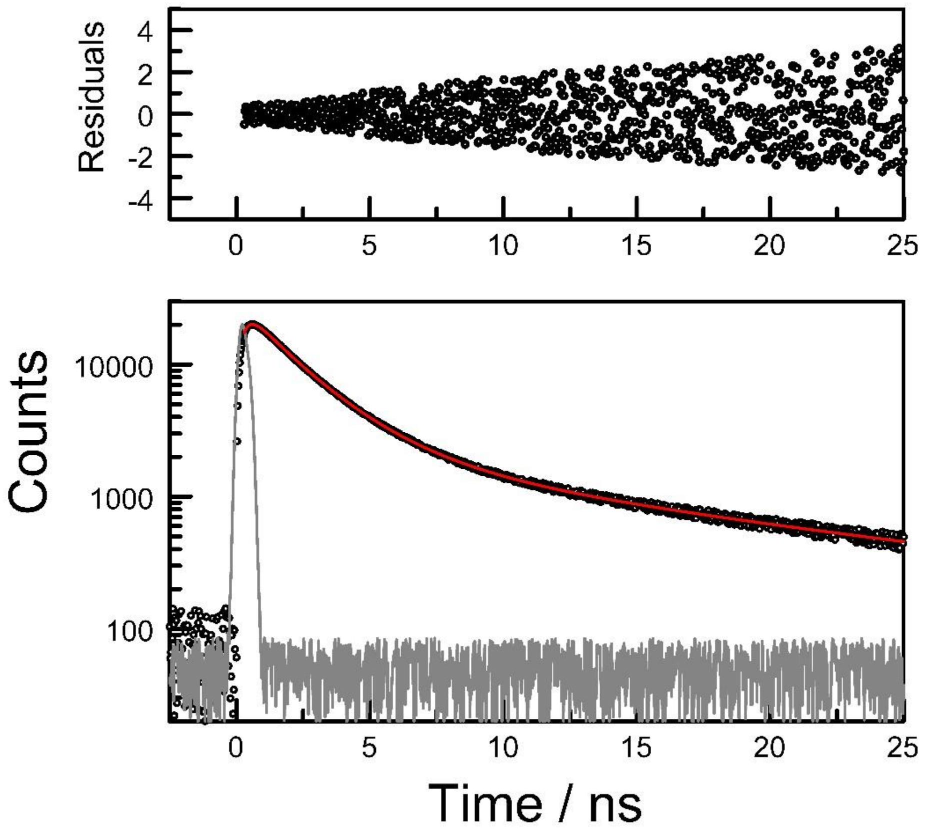

Figure S77. Example of a fluorescence decay curve recorded for pyrene emission from **PY-P$_8$-PER** in chloroform with excitation at 330 nm. Detection was made at 370 nm. The instrument response function is shown as a grey curve while the red line running through the data points (open circles) corresponds to a non-linear least-squares fit to a distributive model with four components. The CPC has a maximum of *ca*. 40,000. The upper panel shows the weighted reduced residuals, which indicates that the overall fit is excellent. The fit was made from just short of the CPC to 25 ns.

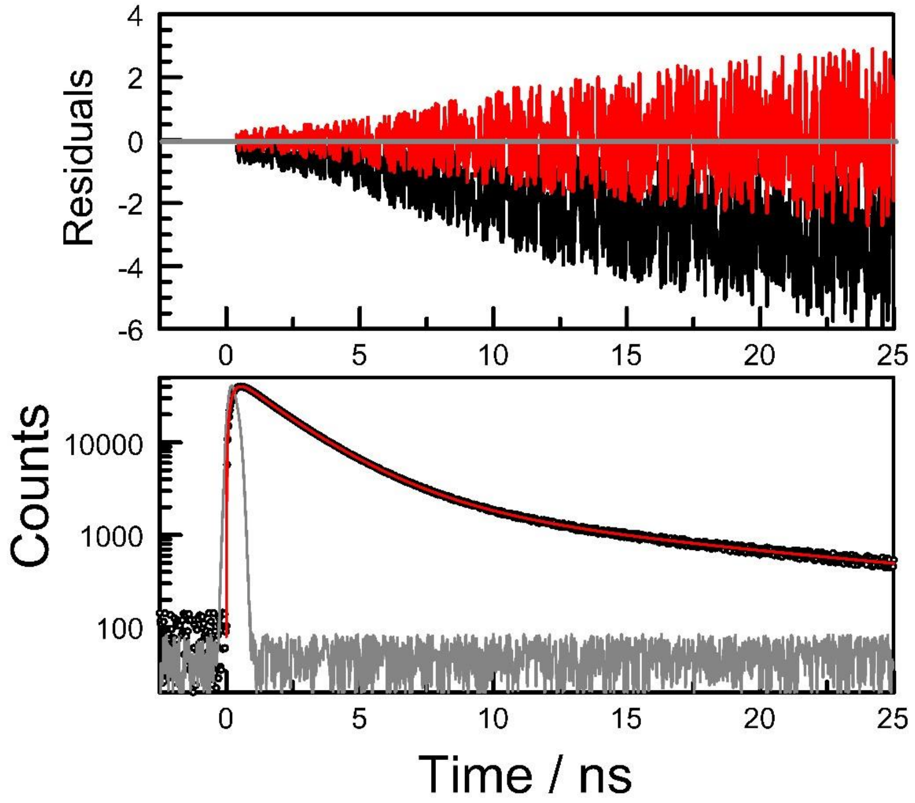

Figure S78. Example of a fluorescence decay curve recorded for pyrene emission from **PY-P_8-PER** in chloroform with excitation at 330 nm. Detection was made at 370 nm. The instrument response function is shown as a grey curve while the red line running through the data points (open circles) corresponds to a non-linear least-squares fit to a distributive model with four components. The CPC has a maximum of *ca*. 40,000. The upper panel shows the weighted reduced residuals. The black curve corresponds to the original input entries without subsequent optimisation while the red curve corresponds to the final optimised fit.

S9. References

S1. R. C. Clark and J. S. Reid, *Acta Cryst*. **1995**, *A51*, 887-897.

S2. O. V. Dolomanov, L. J. Bourhis, R. J. Gildea, J. A. K. Howard and H. Puschmann, *J. Appl. Cryst*. **2009**, *42*, 339-341.

S3. G. M. Sheldrick, *Acta Cryst*. **2008**, *A64*, 112-122.

S4. G. M. Sheldrick, *Acta Cryst*. **2015**, *A71*, 3-8.

S5. J. B. Birks, *Rep. Prog. Phys*. **1975**, *38*, 903-974.

S6. H. H. T. Al-Sharif, R. Ziessel, P. G. Waddell, C. Dixon and A. Harriman, *J. Phys. Chem. A* **2020**, *124*, 2160-2172.

S7. C. A. Parker and W. T. Rees, *Analyst* **1960**, *85*, 587−600.

S8. O. J. Woodford, R. Ziessel, A. Harriman, C. Wills, A. A. Alsimaree and J. G. *Knight, Spectrochim. Acta Part A Mol. Biomol. Spectrosc*. **2019**, *208*, 57-64.

S9. L. Porres, A. Holland, L.-O. Pålsson, A. P. Monkman, C. Kemp and A. Beeby, *J. Fluoresc*. **2006**, *16*, 267−272

S10. O. A. Bozdemir, D. D. Gultekin and A. Harriman, *J. Phys. Chem. A* **2020**, *124*, 10736-10747.

S11. K. H. Grellmann and H. G. Scholtz, *Chem. Phys. Lett*. **1979**, *62*. 64-71.

S12. C. A. Slate, R. A. Binstead, T. J. Meyer and B. W. Erickson, *Lett. Peptide Sci*. **1999**, *6*, 61-69.

S13. (a) S. Kakinoki, Y. Hirano and M. Oka, *Polym. Bull*. **2005**, *53*, 109-115. (b) M. Kümin, L.-S. Sonntag and H. Wennemers, *J. Am. Chem. Soc*. **2007**, *129*, 466-467. (c) D. S. Caswell and T. G. Spiro, *J. Am. Chem. Soc*. **1987**, *109*, 2796-2800.

S14. B. Valeur, *Molecular Fluorescence: Principles and Applications*; WILEY-VCH Verlag GmbH: Weinheim, Germany, 2002, p 29.

S15. R. M. Hochstrasser, *J. Chem. Phys*. **1960**,*33*,459-463.

S16. T. Förster, Zwischenmolekulare energiewanderung und fluoreszenz. *Ann. Phys.* 1948, **437**, 55–75.

S17. T. A. Halgren, *J. Comput. Chem.* **1996**, *17*, 490-519.

S18. M. W. Schmidt et al., *J. Comput. Chem.* **1993**, *14*, 1347-1363.

S19. D. Jacquemin, V. Wathelet, E. A. Perpete and C. Adamo, *J. Chem. Theor. Comput*. **2009**, *5*, 2420-2435.

S20. J. P. Merrick, D. Moran and L. Radom, *J. Phys. Chem. A* **2007**, *111*, 11683-11700.

S21. A. V. Marenich, C. J. Cramer and D. G. Truhlar, *J. Phys. Chem. B* **2009**, *113*, 6378-6396.

S22. J. Tomasi, B. Mennucci and R. Cammi, *Chem. Rev*. **2005**, *105*, 2999-3093.

S23. S. Grimme, *J. Comput. Chem*. **2006**, *27*, 1787-1799.

S24. S. B. Ozkan and H. Meirovitch, *J. Phys. Chem. B* **2003**, *107*, 9128-9131.

S25. D. V. O'Connor and D. Phillips, *Time-Correlated Single Photon Counting*, Elsevier, London, 1984. ISBN 978-0-12-524140-3

S26. (a) D. R. James, Y.-S. Liu, P. De Mayo and W. R. Ware, *Chem. Phys. Lett*. **1985**, *120*, 460-465. (b) D. R. James and W. R. Ware, *Chem. Phys. Lett*. **1986**, *126*, 7-11.

S27. H. Lemmetyinen, N. V. Tkachenko, B. Valeur, J.-I. Hotta, M. Ameloot, N. P. Ernsting, T. Gustavsson and N. Boens, "Time-resolved fluorescence methods (IUPAC Technical Report)" *Pure Appl. Chem.* **2014**, *86*, 1969-1998.

S28. D. F. Eaton. *Pure Appl. Chem.* **1990***, 62*, 1631-1644.
